# Temporal association between human upper respiratory and gut bacterial microbiomes during the course of COVID-19 in adults

**DOI:** 10.1038/s42003-021-01796-w

**Published:** 2021-02-18

**Authors:** Rong Xu, Renfei Lu, Tao Zhang, Qunfu Wu, Weihua Cai, Xudong Han, Zhenzhou Wan, Xia Jin, Zhigang Zhang, Chiyu Zhang

**Affiliations:** 1 Shanghai Public Health Clinical Center, Fudan University, Shanghai, China; 2 Pathogen Discovery and Evolution Unit, Institut Pasteur of Shanghai, Chinese Academy of Sciences, Shanghai, China; 3 Clinical Laboratory, Nantong Third Hospital Affiliated to Nantong University, Nantong, China; 4 State Key Laboratory for Conservation and Utilization of Bio-Resources in Yunnan, School of Life Sciences, Yunnan University, Kunming, Yunnan China; 5 Medical Laboratory of Taizhou Fourth People’s Hospital, Taizhou, China

**Keywords:** Viral infection, Microbiome, Prognostic markers, SARS-CoV-2

## Abstract

SARS-CoV-2 is the cause of COVID-19. It infects multiple organs including the respiratory tract and gut. Dynamic changes of regional microbiomes in infected adults are largely unknown. Here, we performed longitudinal analyses of throat and anal swabs from 35 COVID-19 and 19 healthy adult controls, as well as 10 non-COVID-19 patients with other diseases, by 16 S rRNA gene sequencing. The results showed a partitioning of the patients into 3-4 categories based on microbial community types (I-IV) in both sites. The bacterial diversity was lower in COVID-19 patients than healthy controls and decreased gradually from community type I to III/IV. Although the dynamic change of microbiome was complex during COVID-19, a synchronous restoration of both the upper respiratory and gut microbiomes from early dysbiosis towards late more diverse status was observed in 6/8 mild COVID-19 adult patients. These findings reveal previously unknown interactions between upper respiratory and gut microbiomes during COVID-19.

## Abstract (web summary)

Rong Xu, Renfei Lu, Tao Zhang, Qunfu Wu, and colleagues perform a longitudinal analysis of throat and gut microbiomes on adult COVID-19 patients. They find initially lower bacterial diversity in COVID-19 patients, but demonstrate a restoration of microbiome diversity throughout disease progression for patients with mild forms of the disease.

## Introduction

COVID-19, a severe respiratory disease caused by a novel virus SARS-CoV-2^1,2^, has led to a devastating global pandemic. It typically presents as an asymptomatic infection or manifests mild respiratory symptoms, but more likely develops into severe pneumonia and cause death in elderly over 60 years of age or those having comorbidities^3,4^. The biological mechanisms behind the varied clinical presentations are not fully understood.

The microbiota plays a major role in modulating human health status by shaping the immune system and maintaining homeostasis^5^. In several respiratory viral infections (RVs), the microbial composition in the respiratory tract and the gut have been linked to the occurrence and severity of disease and affect subsequent respiratory health^6,7^, through increasing airway susceptibility to infection by other RVs and/or the colonization of pathogenic bacteria^8–10^. It is therefore reasonable to posit that the new respiratory infection COVID-19 may also interact with microbiota.

Indeed, some recent studies have shown that SARS-CoV-2 infects human gut enterocytes and causes diarrhea^11,12^. Altered gut microbiota has been observed in COVID-19 patients leading to an enrichment of opportunistic pathogens and a depletion of beneficial bacteria^13,14^. However, changes in the respiratory microbiome have not been evaluated in COVID-19. Furthermore, despite persistent alterations in the gut microbiota has been reported using longitudinal stool samples collected in COVID-19 patients^13^, no study has examined whether there is any association between the respiratory and gut microbiota during the cause of disease. In this study, we investigated for the first time the dynamics of both the upper respiratory and gut microbiomes in a cohort of COVID-19 patients and controls, and discovered a pattern of synchronous changes in these two microbiomes.

## Results

### Study cohort

The study subjects included 35 adult COVID-19 patients from 17 to 68 years of age, 19 healthy adults, and 10 non-COVID-19 patients (NP) with other diseases. Except patient p09 who had severe clinical symptoms, all other 34 COVID-19 patients had mild clinical symptoms. All confirmed COVID-19 cases were hospitalized in China, even if they had no symptoms. A total of 146 specimens including 37 pairs of both throat and anal swabs were collected from these COVID-19 patients (Supplementary Fig. S1). High-throughput sequencing of the V4-region of bacterial 16 S rRNA gene was performed for all samples.

### Respiratory microbiome dynamics in COVID-19

**Fig. 1:**
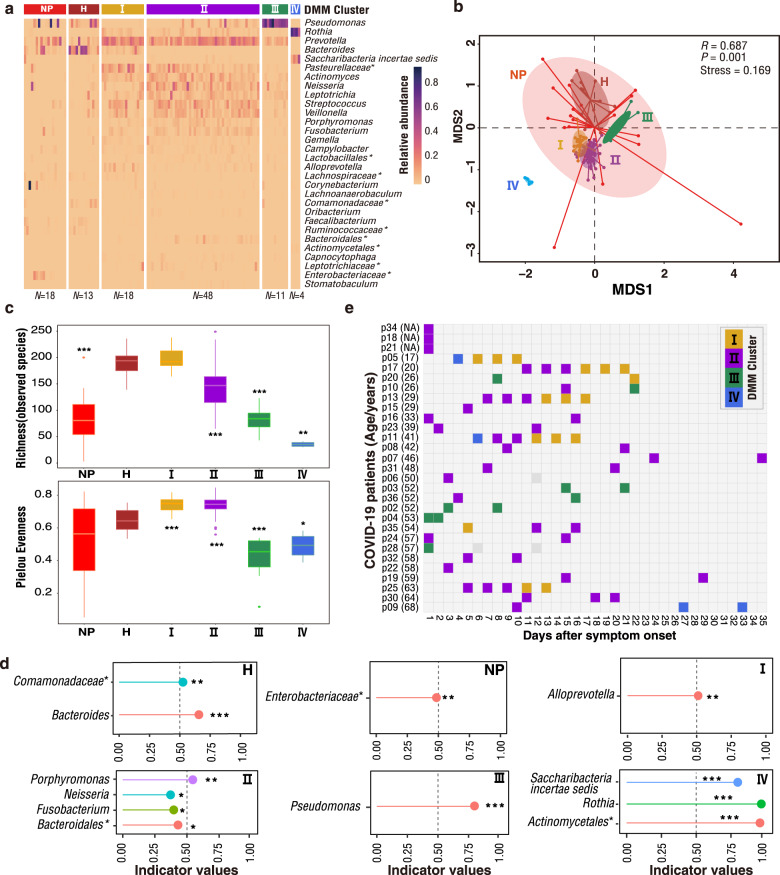
DMM clustering of 16 S rRNA gene-sequencing data of throat microbiota (*N* = 112). Dirichlet multinomial mixtures (DMM) modelling was applied to 16 S rRNA gene sequencing. The entire dataset formed six distinct clusters based on lowest Laplace approximation. Bacterial taxa marked by the stars represent unclassified bacteria genera. **a** Heatmap showing the relative abundance of the 30 most dominant bacterial genera per DMM cluster. The stars represent unclassified genera. NP, enriched in Non-COVID-19 patients. H, enriched in Healthy individuals. I–IV enriched in COVID-19 patients. **b** Nonmetric multidimensional scaling (NMDS) visualization of DMM clusters using Bray–Curtis distance of throat bacterial genera. The ANOSIM statistic R closer to 1 with <0.05 *P-*value suggest significant separation of microbial community structures. The stress value that was lower than 0.2 provides a good representation in reduced dimensions. **c** Boxplots showing the alpha-diversity (richness and evenness) per each DMM cluster. **d** Indicators of airway microbial community types (DMM clusters) identified from top 30 genus contributing to throat microbial community typing (DMM clustering) in **a**. The length of lines represents the indicator value. *P < 0.05, **P < 0.01, and ***P < 0.001. **e** Dynamic shift of four throat microbial community types (DMM clusters) in different COVID-19 stages. Empty boxes indicate samples were unavailable in COVID-19 patients. Ages (years) were shown in parenthesis. NA unavailable.

The 16S-rRNA gene sequences of all throat swabs were resolved into 3126 amplicon sequence variants (ASVs) representing 17 known phyla including 209 known genera (Supplementary Data 1). Six throat microbial community types (or clusters) were identified using the Dirichlet Multinomial Mixtures (DMM) modelling based on the lowest Laplace approximation (Fig. 1a) and visualized by Nonmetric Multidimensional Scaling (NMDS) based on Bray–Curtis distance (Fig. 1b). Thirteen of 19 specimens of healthy adults (H) formed an independent cluster defined as community type H. The vast majority of the specimens of COVID-19 patients were divided into four clusters, herein named community types I–IV (Fig. 1a). Other specimens from six COVID-19 patients were clustered with those from 10 non-COVID-19 patients and two healthy controls. Because this cluster has a significantly higher proportion of NP patients (55.6%, *P* < 0.01) than COVID-19 patients (33.3%) and healthy controls (11.1%) (Supplementary Fig. S2), it was designated as community type NP. All COVID-19-related community types, as well as the NP type, were significantly distant from the H type. Community types III and IV were not only separated from the types I and II, but also from each other (Fig. 1b). A decrease in alpha-diversity of the microbiome was observed from type I to IV, and significantly lower richness and evenness were observed in community types III and IV, compared with the H type (Fig. 1c). Similar decreasing trends of alpha-diversity were also observed when the Margalef’s indexes were used to control the effect of sample size (Supplementary Fig. S3)^15^. To more directly demonstrate that the variation of throat microbial composition is an indicator of COVID-19 disease stages, the community type-specific indicator taxa were identified based on the top 30 microbial genera (Fig. 1d). The type H was characterized by bacterial genus *Bacteroides* (predominant taxa in the lung of healthy individuals) and unclassified *Comamonadaceae*, whereas the NP type was marked by proinflammatory *Enterobacteriaceae* members. In contrast, the indicator bacteria of four COVID-19-related community types were *Alloprevotella* in type I, *Porphyromonas*, *Neisseria*, *Fusobacterium* and unclassified *Bacteroidales* in type II, *Pseudomonas* in type III, and *Saccharibacteria incertae sedis*, *Rothia* and unclassified *Actinomycetales* in type IV (Fig. 1d). Community type I contained *Alloprevotella* genus, as well as abundant *Bacteroides* and *Prevotella* that typically present in the H type (Fig. 1a). Some indicator bacteria substantially enriched in types II and IV belong to opportunistic pathogenic bacteria that may be associated with human diseases such as pneumonia, chronic periodontitis, and bacteremia^16–21^. For example, the identified *Rothia* species in type IV have the highest sequence similarity with *Rothia mucilaginosa* that is often associated with cancer and bacteremia^22^. *Porphyromonas*, *Fusobacterium*, and *Neisseria* enriched in type II typically exist in the nasopharynx, and they are associated with pneumonia or chronic periodontitis. Besides opportunistic pathogenic bacteria, commensals (e.g., *Bacteroidales*) were also enriched in type II. In type III, the identified *Pseudomonas* sequences have the highest sequence similarity (100%) with multiple known nonpathogenic species such as *P. lactis*, *P. paralactis*, *P. canadensis*, *P. tolaasii*, and *P. fluorescens*. For example, *P. lactis* was initially isolated from bovine raw milk, and rarely found in human^23^. Compared with the community type H, a decreased alpha-diversity with high abundance of opportunistic pathogenic and environmental bacteria (nonpathogenic *Pseudomonas* species) in community types II–IV might imply a disruption of microbiome homeostasis (dysbiosis) in the respiratory tract (Supplementary Data 1 and Fig. S4). Lower alpha-diversity with enrichment of proinflammatory *Enterobacteriaceae* indicates that the type NP represents another status of microbial dysbiosis.

According to indicator bacteria and alpha-diversity characteristics, the microbial community types from I to IV may represent a progressive imbalance of the respiratory microbiome (Fig. 1c-d). Among all throat specimens from COVID-19 patients, 47 (56.6%) belong to community type II (Supplementary Fig. S2), indicating that altered upper respiratory microbiome by COVID-19 was mainly characterized by community type II. Longitudinal analysis showed that community types with relatively lower alpha-diversity are more likely to have appeared in early specimens (Fig. 1e), but the diversity did not significantly correlate with the time after symptom onset regardless of being analyzed at the overall and individual levels (Supplementary Fig. S5-S6). Among 22 COVID-19 adults who had specimens at two or more timepoints, over half (12, 54.5%) maintained a relatively stable microbiome community types, and the others had community types altered over time. An obvious throat microbiome recovery from types IV or II in early specimens to type I in late specimens was observed in five patients (p17, p25, p13, p11, and p05) with four or more consecutive specimens (Fig. 1e), accompanied with the restoration of throat microbiota, appearance of beneficial commensals, and increased bacterial diversity (Supplementary Fig. S4). An opposite pattern was observed in four patients who had microbiome composition shift from early higher-diversity types (I or II) to later lower-diversity type (II–IV), implying a worsening of the throat microbiome. In particular, the only one severe case (p09) experienced a community type shift from type I on day 10 to type IV on day 27, and sustained type IV to at least day 33 after symptom onset (Fig. 1e). Accompanied with this shift, opportunistic pathogenic bacteria *Saccharibacteria incertae sedis* and *Rothia* were substantially enriched at late stage (Supplementary Fig. S4). These indicate that the dynamic changes of upper respiratory microbiome caused by COVID-19 was heterogenous among different individuals.

### Gut microbiome dynamics in COVID-19

**Fig. 2:**
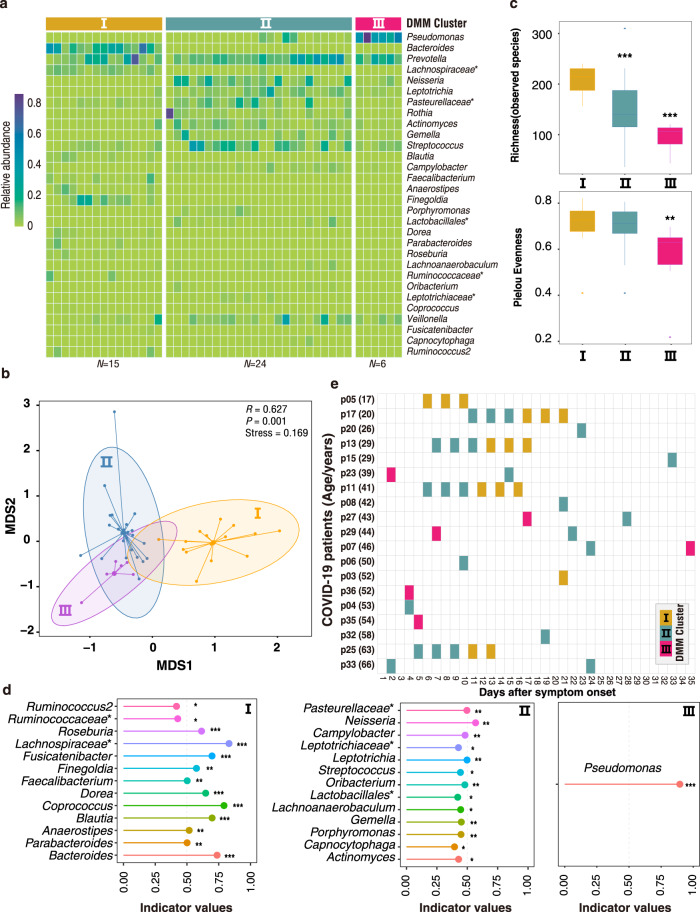
DMM clustering of 16 S rRNA gene-sequencing data of gut microbiota (*N* = 45). Dirichlet multinomial mixtures (DMM) modelling was applied to 16 S rRNA gene sequencing. The entire dataset formed three distinct clusters based on lowest Laplace approximation. All samples were collected from COVID-19 patients. Bacterial taxa marked by the stars represent unclassified bacteria genera. **a** Heatmap showing the relative abundance of the 30 most dominant bacterial genera per DMM cluster. **b** Nonmetric multidimensional scaling (NMDS) visualization of DMM clusters using Bray–Curtis distance of gut bacterial genera. The ANOSIM statistic R closer to 1 with <0.05 *P-*value suggest significant separation of microbial community structures. The stress value that was lower than 0.2 provides a good representation in reduced dimensions. **c** Boxplots showing the alpha-diversity (richness and evenness) per each DMM cluster. **d** Indicators of gut microbial community types (DMM clusters) identified from top 30 genus contributing to gut microbial community typing (DMM clustering) in **a**. *P < 0.05, **P < 0.01, and ***P < 0.001. **e** Dynamic shift of gut microbial community types (DMM clusters) in different COVID-19 stages. Empty boxes indicate samples were unavailable in COVID-19 patients. Ages (years) were shown in parenthesis.

To expand the scope of this research, a total of 1,940 ASVs were recovered from the 16S-rRNA gene sequences of all anal swabs, representing 13 known phyla including 182 known genera (Supplementary Data 1). The gut microbial communities of COVID-19 patients formed three distinct community types I–III (Fig. 2a-b). The richness and evenness of the gut microbiome decreased from type I to III (Fig. 2c). Indicator analyses showed that type I was primarily characterized by healthy gut genera including *Bacteroides* genus and several known butyrate-producing bacteria (e.g., *Faecalibacterium*, *Roseburia*, *Blautia*, and *Coprococcus*) and one opportunistic pathogenic bacterium (*Finegoldia*) (Fig. 2d)^24–29^. The indicators of type II mainly contain various pathogenic or opportunistic pathogenic bacteria (e.g., *Neisseria* and *Actinomyces*). In community type III, the gut microbiota was dominated by *Pseudomonas*, implying a severe dysbiosis. We also used the community types I–III to examine the dysbiosis status of the gut microbiome.

A shift of the gut microbiome from the lower-diversity community type (II or III) towards a higher-diversity type (I or II) was observed over time in 7/10 patients who had anal swabs at different timepoints (Fig. 2e). Accompanied with the shift, a clear trend of increased bacterial diversity and the relative abundance of beneficial commensals (e.g., *Bacteroides* and *Faecalibacterium*) was observed in the gut microbiota from early to late stages of COVID-19 (Supplementary Fig. S7), suggesting a restoration of gut microbiota. Two patients maintained a stable microbiome community types, and only one patient had an opposite shift of community type from higher-diversity type II to lower-diversity community type III.

### Association between the respiratory and gut microbiomes in COVID-19

**Fig. 3:**
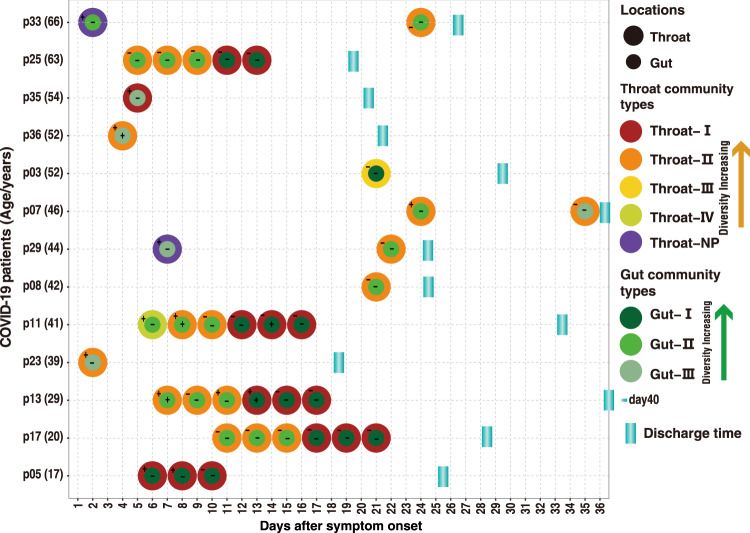
Dynamic change of bacterial community types (DMM clusters) in respiratory tract and gut of patients with mild COVID-19. Covariation dynamics of throat and gut microbial communities of 13 COVID-19 patients. Filled circles indicate the presence of microbial community types. Positive or Negative detections of SARS–CoV-2 in gut or throat are implicated by + or − symbols, respectively. Age (years) of each COVID-19 adult was shown in brackets.

Most paired throat and anal swabs showed the same or similar community type levels (Fig. 3). In particular, the shift of microbiome community types over time appeared to match between the throat and the gut in 6/8 patients who had two or more paired specimens at different timepoints (Fig. 3). Synchronous improvement of both the respiratory and gut microbiomes from early lower-diversity community type towards late higher-diversity type occurred in six patients (p05, p17, p13, p11, p25, and p29). One patient (p33) experienced an improved respiratory microbiome but maintained an unchanged gut community type up to day 24. One case (p07) had a worsen gut microbiome from day 24 to day 35 but maintained an unchanged respiratory community type. Because of no available anal specimens, we were unable to assess whether the gut microbiota, like the respiratory microbiota, shifted from higher-diversity type to lower-diversity type over time in the severe case (p09) (Fig. 1e). Except for the duration of COVID-19, the upper respiratory and gut microbial community divergence seemed not to be significantly associated with age, gender, antibiotics use, and detection of SARS-CoV-2 RNA (Supplementary Figs. S8-S9). The alpha-diversity of the microbiome was also not significantly associated with the time after symptom onset (Supplementary Fig. S5-S6), and clinical parameters, except for a weak association between the upper respiratory microbiome richness and NK cell counts (Supplementary Fig. S10). Furthermore, the richness of both upper respiratory and gut microbiome appeared to be negatively correlated with the serum levels of lipopolysaccharides (LPS) (Supplementary Fig. S11 and Table S1).

**Fig. 4:**
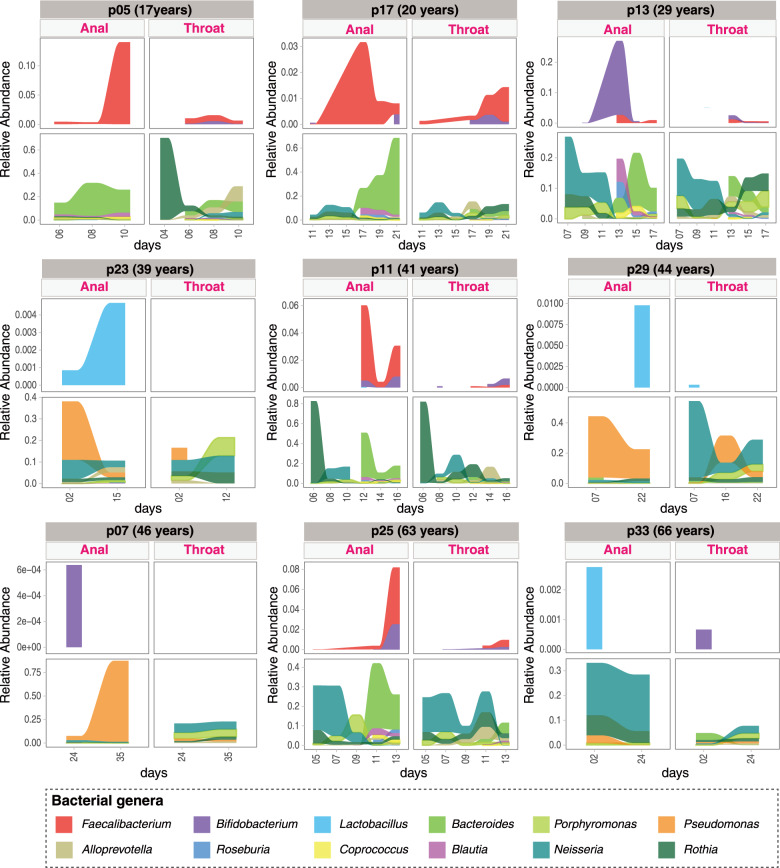
Dynamic change of 12 key taxa in respiratory tract and gut of patients with mild COVID-19. Key taxa of DMM clusters and several core functional gut bacteria were shown in nine mild COVID-19 adults with at least two timepoints of sampling. Linked to Fig. 1a, Fig.2a, and Supplementary Figs. S4 and S7.

We further selected the top indicator bacteria with >0.5 indicator values from each community type (Figs. 1d and 2d) and several major core functional bacteria (e.g., *Faecalibacterium*, *Lactobacillus*, and *Bifidobacterium*) in gut as the representative bacteria to assess their dynamic changes in relative abundance over time (Fig. 4). In general, the relative abundance of *Bifidobacterium, Lactobacillus* and/or *Faecalibacterium* appeared to be negatively associated with the relative abundance of the opportunistic pathogens (e.g., *Rothia* and *Neisseria*), especially in the gut microbiome. An obvious decrease in the relative abundance of opportunistic pathogenic bacteria was accompanied by an increase in the relative abundance of resident commensals *Bacteroides* in gut microbiome over time in five patients having three or more longitudinal samples (Fig. 4 and Supplementary Figs. S4 and S7). Moreover, a substantially decreased abundance of *Pseudomonas* was observed in both organs in another two patients (p23 and p29). The relative abundance of *Pseudomonas* increased only in the gut of patient p07 accompanied by a decreasing bacterial diversity (Fig. 4 and Supplementary Fig. S6).

### Bacteria–bacteria co-occurrence networks

There were four indicator bacteria genera (*Porphyromonas*, *Neisseria*, and *Fusobacterium* in type II and *Pseudomonas* in type III) in the throat microbiome that had been identified as the indicators of gut microbial community types II and III in COVID-19 patients (Supplementary Fig. S12). Apart from the shared indicators, oropharyngeal pathogenic bacteria *Capnocytophaga* and *Actinomyces* were also identified as indicators of the gut microbial community type II (Figs. 1d and 2d)^30,31^. Because community types II and III often appeared in the early stage of COVID-19 (Figs. 1e and 2e), the appearance of these oropharyngeal bacteria in the gut suggested that a crosstalk between the respiratory and gut microbiomes occurred by frequent bacterial translocation during the early stage. High-serum LPS is due to microbial translocation, and was often associated with virus infection. High-serum LPS levels were also detected in some COVID-19 patients (Supplementary Table S1), suggesting that bacteria translocation might play a role in the crosstalk between the respiratory and gut microbiomes.

**Fig. 5:**
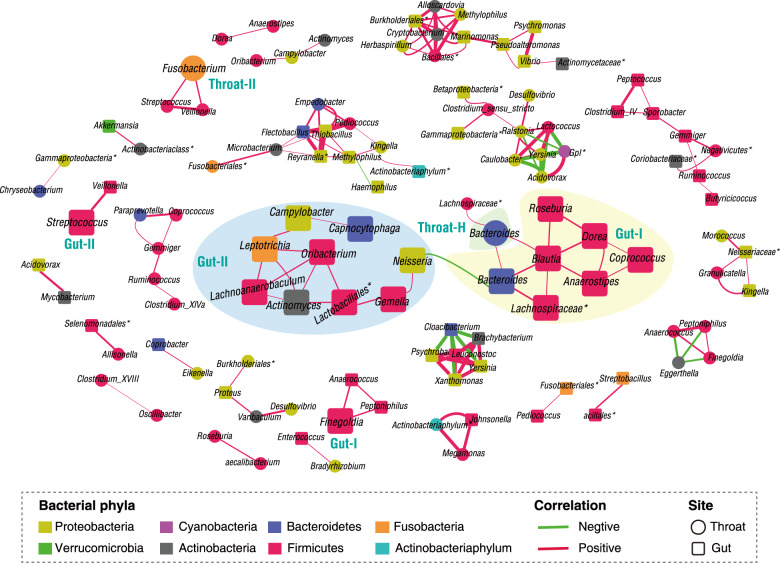
Co-occurrence networks of gut and throat microbiota within 13 COVID-19 patients. Pearson correlation was employed to calculate correlation coefficient (r) between bacterial genus pairs based on their relative abundances. Co-occurred pairs with *r* > 0.7 under FDR-adjusted *P* < 0.05 were shown and visualized by Cytoscape version 3.8.0. Edges were sized based on r values. The bigger squares or circles were indicators in Figs. 1d and 2d.

To further investigate the association between the respiratory and gut microbiomes, we performed co-occurrence network analysis using paired specimens from 13 patients. We constructed a co-occurrence network consisting of a total of 153 co-occurred pairs with Pearson correlation |r | > 0.7 under FDR-adjusted *P* < 0.05 (Fig. 5). Bacteria in the same niche tended to have close co-occurrence relationship, and the cross-talks of microbial compositions between the upper respiratory tract and the gut were also observed. In particular, a competitive relationship between Gut-type-II and Gut-type-I was mediated by a significantly negative interaction between gut bacterial genera *Neisseria* and *Bacteroides* (Fig. 5), which might determine the microbiome shift from Gut-type-II to Gut-type-I during the COVID-19 disease progression. Furthermore, core resident commensals *Bacteroides* appeared to mediate the crosstalk between Throat-type H and Gut-type-I, which might modulate the restoration of throat and gut microbiota during course of COVID-19.

## Discussion

Whether SARS-CoV-2 infection alters microbiota to affect COVID-19 disease progression is an important question that needs answers. In this study, we made three major observations. First, the upper respiratory and gut microbiota compositions of COVID-19 adults can be characterized by four (I–IV) and three (I–III) community types, respectively, and these types possibly reflect different levels of balance between the more diverse microbiota (type I) and dysbiosis (type II–IV). Second, the microbiome community types with lower alpha-diversity more likely appears in the early phase of COVID-19, and upper respiratory and gut microbiomes altered by COVID-19 are mainly characterized by community type II with predominance of *Bacteroidales*, *Fusobacterium*, *Porphyromonas*, *Prevotella*, *Neisseria* and some opportunistic pathogens in the former, and *Neisseria* in the latter. Third, the dynamic change of community types is synchronous in the upper respiratory tract and gut.

SARS-CoV-2 infects cells through ACE2 receptor^2^, which is highly expressed in respiratory and intestinal epithelial cells^32^. The infection can trigger the cytokine storm to cause local pathological damage^33,34^. As an open system with direct contact with environment and the primary site for respiratory infections, the upper respiratory tract microbiota is more easily affected by respiratory virus infections, but the effect of SARS-CoV-2 infection has not been examined yet. In this study, we observed alterations of the upper respiratory microbiota in COVID-19 adults, and presented data on the dynamic change of the respiratory microbiome composition over time. The upper respiratory microbiome of the COVID-19 adults was characterized by four bacterial community types I–IV, which reflect the different levels of the normal microbiome to dysbiosis. The community types with lower alpha-diversity and high enrichment of opportunistic pathogenic bacteria and *Pseudomonas* often appeared in early throat specimens (e.g., first several days after symptom onset), indicating that SARS-CoV-2 infection results in a very rapid dysbiosis in upper respiratory tract. A restoration of the upper respiratory microbiome from dysbiosis towards more diverse types was observed over time in some with mild disease, whereas prolonged or worsening microbiome appeared in a few others including the only one severe case (p09).

Intestinal enterocytes that express ACE2 are also the target of SARS-CoV-2, which further upregulates the expression of ACE2, leading to a longer viral RNA shedding time in the gut than respiratory tract^11,32^. The early infection microbiome composition with abundant pathogenic bacteria (e.g., *Coprobacillus*, *Clostridium ramoaum*, and *Clostridium hathewayi*) had been associated with the fecal levels of SARS-CoV-2 and COVID-19 severity in a previous study^32^. However, the sampling time was relatively late in that study (about 14 days after symptom onset), therefore unable to determine whether the microbiome status is a consequence of early SARS-CoV-2 infection, or a cause of disease severity. We also observed alterations of the gut microbiota during COVID-19 in adults, and found some opportunistic pathogenic bacteria (e.g. *Streptococcus*, *Rothia*, *Veillonella*, *Actinomyces* and *Actinomyces*) reported in the previous observations^13,14^. However, distinct from the previous studies, we identified three community types (I–III) that can characterize the changes of gut microbiome over time. Similar to the observation in the upper respiratory microbiome, community types (i.e., II and III) with lower alpha-diversity often appeared in early specimens, supporting the early effect of SARS-CoV-2 on the gut microbiome. A restoration with the community type shifted from low-diversity type II to high-diversity type I over time was observed in at least four patients. However, *Pseudomonas-*dominated community type III showed a slow improvement towards community type II in three patients. In particular, the temporal dynamic changes of the microbiomes matched between the upper respiratory tract and the gut, indicating a close association in microbiota between both body sites, possibly via the “airway-gut axis”^35^.

The reason for the fast dysbiosis in both the upper respiratory tract and the gut of COVID-19 patients might be associated with the early-stage inflammation induced by SARS-CoV-2 infection, which leads to a fast loss of beneficial commensals and the colonization and growth of opportunistic pathogenic bacteria (Supplementary Fig. S13). The use of empirical antibiotics in some patient during the early stages of the pandemic may exacerbate the dysbiosis in the upper respiratory tract and gut. Therefore, the microbiome composition with enrichment of opportunistic pathogenic bacteria (e.g., *Rothia* and *Neisseria*) was observed in both throat and gut microbiomes during the first several days after symptom onsets. Because the upper respiratory tract is more receptive to both exogenous and indigenous microbes than the gut^7,36^, the dysbiosis of upper respiratory microbiome appeared to be worse and occurred earlier than that of the gut microbiota, as manifested by lower diversity and richness and more indicators of opportunistic pathogenic bacteria in the former than in the latter. The damaged upper respiratory tract mucosa enables some oral taxa to be translocated to the gut, worsening the gut bacterial community (Supplementary Fig. S13). There are several possible mechanisms to explain the oropharyngeal bacterial translocation to the gut. First, inflammation induced by SARS-CoV-2 infection damaged the mucosal tissues and increased mucosal permeability of the airway, lung, and gut^7,37^, which then enables bacterial translocation. Second, bacteria migrated from the oropharyngeal site to the gut via swallowing and passage through the gastrointestinal tract. Third, immune responses induced by infection applied similar selective pressures to the microbiota at both sites.

Gut microbiota plays an important role in human health by shaping local immunity and remodeling mucosal tissues^38^. It is relatively more stable and resilient than the respiratory microbiota, and it may affect the latter by crosstalk between these two organs along the airway-gut axis^35,36^. In spite of longer duration of SARS-CoV-2 shedding in the gut than in the respiratory tract, gut microbiota appeared to have a synchronous change with the respiratory microbiota (Fig. 3). Although the dynamic change of the microbiome was relatively divergent and independent of early microbiome community types, synchronous restoration of both the respiratory and gut microbiomes from early low diverse status towards late more diverse status was observed in six (75%) mild COVID-19 adult patients who had two or more paired specimens at different timepoints. Age, gender and antibiotics use seemed not to be linked to restoration of the microbiome, implying potential contributions from other factors such as diet and genetic background. The identification of some opportunistic pathogenic bacteria (*Neisseria, Porphyromonas, Rothia*, *Actinomycetales*, and *Saccharibacteria*) in more dysbiosis community types II and IV might imply a need for microbiota-based personalized antibiotics treatment against these specific pathogens. As the most common microbiome status in COVID-19 patients, the community type II represents a crucial intermediate stage during the restoration of the microbiome from dysbiosis towards more diverse microbiome. It was characterized by *Neisseria, Fusobacterium*, and *Porphyromonas*. *Fusobacterium* and *Porphyromonas* are the common commensals in the oropharynx and the gut^19,21^, while *Neisseria* generally presents in the lung. The appearance of lung *Neisseria* in both the upper respiratory tract and the gut, implying bacteria translocations along the “airway-lung-gut axis”^39^. The bacteria translocations may be the consequence of increased permeability among these organs caused by local inflammation^40^, as evidenced by high levels of serum LPS. The *Bifidobacterium* and some butyrate-producing bacteria (e.g., *Faecalibacterium*) can improve the inflammatory conditions and regulate innate immunity by down-regulating ACE2 expression, and activating the corresponding signaling pathways^27,41^. During the restoration of the microbiota, these beneficial bacteria gradually occupied the ecological niches in the gut and respiratory tract, and governed the microbial communities in both organs by replacement of opportunistic pathogenic bacteria (e.g., *Rothia* and *Neisseria*) over time. However, a progressively worsening in the upper respiratory and gut microbiome might be associated with severe cases of COVID-19.

One noted limitation of this study is the relatively small patient number. Our results may not be representative of all patient groups, and the observed dynamic changes of the microbiome in both the upper respiratory tract and gut may be further validated in a larger cohort. Another limitation of the study is that the dynamic changes of the microbiome were only followed up to 35 days after symptom onset. Whether COVID-19 exerts long-term effect of on the microbiomes is an interesting question for further investigation. Technically, the use of only 16 S data may restrict our ability to identify specific bacteria species and infer their functions.

In summary, we revealed for the first time an association between the upper respiratory and gut microbiota during COVID-19 disease progression, and observed synchronous changes of microbiota in both organs mainly from early dysbiosis towards later more diverse status in a proportion of adults with mild COVID-19 (Supplementary Fig. S13). In the absence of specific antiviral drugs and vaccines for COVID-19, our findings may have clinical implications. For instance, some indicator bacteria (e.g., opportunistic pathogenic and beneficial butyrate-producing bacteria) may potentially be crucial biomarkers for clinical treatment decision making and prognostic evaluation. The measurement of predominant short-chain fatty acid (especially butyrate) concentration in fecal samples may be useful in early clinical diagnosis. Apart from the routine treatment efforts (e.g., nonspecific antiviral and supportive treatments)^42^, precision intervention and modulation of the gut and respiratory microbiota may offer novel therapeutic alternatives, such as personalized antibiotics therapy to inhibit certain opportunistic pathogenic bacteria. Moreover, COVID-19 tailored probiotics (e.g., *Bifidobacterium* and *Faecalibacterium*), prebiotics (e.g., xylooligosaccharide) treatment, or symbiotic treatments might be applied to modulate the gut and respiratory microbiota to facilitate the recovery of COVID-19 patients.

## Methods

### Study population

A total of 64 subjects, including 35 laboratory-confirmed COVID-19 patients, 10 SARS-CoV-2 negative patients with various diseases (non-COVID-19) and 19 healthy adults were enrolled in this study. COVID-19 was diagnosed in adult patients according to the National Guidelines for Diagnosis and Treatment of COVID-19. The virus RNA was extracted from all samples using a Mag-Bind RNA Extraction Kit (MACCURA, Sichuan, China) according to the manufacturer’s instructions. Then the *ORFlab* and *N* genes of SARS-CoV-2 was detected using a Novel Coronavirus (2019-nCoV) Real Time RT-PCR Kit (Liferiver, Shanghai, China) according to the manufacturer’s instructions. Only the individuals who had at least two consecutive throat swabs been positive for both *ORFlab* and *N* genes of SARS-CoV-2 were defined as COVID-19 patients. All positive specimens of COVID-19 patients were confirmed by Nantong Center for Disease Control and Prevention (CDC) using recommended real-time RT-PCR assay by China CDC. Mild and moderate cases are defined as having clinical symptoms (e.g., fever, cough, etc.) with and without the pneumonia on lung imaging. Severe COVID-19 (adult) is defined as the presence of any one of the following: respiratory rate ≥30 breaths/minute, arterial oxygen saturation ≤93% at rest; PaO_2_/FiO_2_ ≤ 300 mm Hg. The COVID-19 patients were hospitalized at Nantong Third Hospital Affiliated to Nantong University. Among 35 COVID-19 patients, 34 were mild or moderate cases, and only one (P09) was severe case.

Demographic and clinical characteristics of the COVID-19 patients were provided in Supplementary Data 2 and 3^43^. Specimens including throat swabs and anal swabs were collected from the COVID-19 patients at different timepoints during their hospitalization (10–40 days). Sampling was performed using flexible, sterile, dry swabs, which can reach the posterior oropharynx and anus easily (~2 inches) by the professionals at the hospital. At least two throat swabs at different days were available for 32 of 35 COVID-19 patients (Supplementary Fig S1).

Non-COVID-19 control patients were selected from patients hospitalized at the same hospital during the COVID-19 pandemic due to other diseases, and healthy controls were selected from adults who came for routine physical examination and showed no symptoms. Throat swabs of non-COVID-19 patients and healthy controls were collected during their hospital visit.

The study was approved by Nantong Third Hospital Ethics Committee (EL2020006: 28 February 2020). Written informed consents were obtained from each of the involved individuals. All experiments were performed in accordance with relevant guidelines and regulations.

### 16S-rRNA gene sequencing

Bacterial DNA was extracted from the swabs using a QIAamp DNA Microbiome Kit (QIAGEN, Düsseldorf, Germany) according to the manufacturer’s instructions, and eluted with Nuclease-free water and stored at −80 °C until use. The V4 hypervariable region (515–806 nt) of the 16 S rRNA gene was amplified universal bacterial primers^44^. To pool and sort multiple samples in a single tube of reactions, two rounds of PCR amplifications were performed using a novel triple-index amplicon sequencing strategy as described previously^45^. The first round of the PCR (PCR1) amplification was performed with a reaction mixture containing 8 μL Nuclease-free water, 0.5 μL KOD-Plus-Neo (TOYOBO, Osaka Boseki, Japan), 2.5 μL of 1 μM PCR1 forward primer, 2.5 μL of 1 μM PCR1 reverse primer, and 5 μL DNA template. The products of the PCR1 reactions were verified using a 1.5% agarose gel, purified using Monarch DNA Gel Extraction Kit (New England Biolabs, Ipswich, MA, USA), and quantified by a Qubit® 4.0 Fluorometer (Invitrogen, Carlsbad, CA, USA). Equal amounts of purified PXR1 products were pooled, and subjected to the secondary round of PCR (PCR2) amplification. The PCR2 was performed with a reaction mix containing 21 μL Nuclease-free water, 1 μL KOD-Plus-Neo (TOYOBO, Osaka Boseki, Japan), 5 μL of 1 μM PCR2 forward primer, 5 μL of 1 μM PCR2 reverse primer, and 5 μL pooled PCR1 products. The PCR2 products were verified using a 2% agarose gel, purified using the same Gel Extraction Kit and qualified using the Qubit® 4.0 Fluorometer. The amounts of the specific product bands were further qualified by Agilent 2100 Bioanalyzer (Agilent, Santa Clara, CA, USA). Equal molars of specific products were pooled and purified after mixing with AMPure XP beads (Beckman Coulter, Pasadena, CA, USA) in a ratio of 0.8:1. Purified amplicons were paired-end sequenced (2 × 250) using Illumina-P250 sequencer.

### Bioinformatic analysis of 16S-rRNA gene sequence data

Sequenced forward and reverse reads were merged using USEARCH11 software^46^, then demultiplexed according to known barcodes using FASTX-Toolkit^47^. After trimming barcode, adapter and primer sequences using USEARCH11, 19,096,003 sequences were retained with an average of 105508 sequences per sample. After excluding the samples with sequences <1000, 157 samples from 35 COVID-19 patients, 10 non-COVID-19 patients and 13 healthy individuals were subjected to the following analysis.

Because traditional OTU (operational taxonomic units) picking based on a 97% sequence similarity threshold may miss subtle and real biological sequence variation^48^, several novel methods such as DADA2^49^ and Deblur^50^ were developed to resolve sequence data into single-sequence variants. Here, the DADA2 was employed to perform quality control, dereplicate, chimeras remove on Qiime2 platform^51^ with default settings except for truncating sequence length to 250 bp. Finally, an amplicon sequence variant (ASV) table, equivalent to OTU table, was generated and then spitted into gut ASV table (2348 ASVs) and throat ASV table (4050 ASVs). The taxonomic classification of ASV representative sequences was conducted by using the RDP Naive Bayesian Classifier algorithm^52^ based on the Ribosomal Database project (RDP) 16 S rRNA training set (v16) database^53^. To eliminate sequencing bias across all samples, both the gut ASV table and throat ASV table were subsampled at an even depth of 4700 and 3000 sequences per sample, respectively. The ASV coverage of 82.6% (gut) and 77.2% (throat) were sufficient to capture microbial diversity of both sites.

### Identification and characterization of microbial community types

Dirichlet multinomial mixtures (DMM)^54^ is an algorithm that can efficiently cluster samples based on microbial composition, its sensitivity, reliability, and accuracy had been confirmed in many microbiome studies^55–57^. DMM clustering were conducted with bacterial genus abundance from throat and gut microbiota using the command “get.communitytype” introduced by v1.44.1 of mothur^58^. The appropriate microbial community type numbers (DMM clusters) were determined based on the lowest Laplace approximation index. According to sample counts per cluster, the fisher exact test was applied to discover significant associations between each cluster and host conditions (such as healthy controls, COVID-19 patients, and Non-COVID-19 patients) under *P* values that are below 0.05 adjusted by the False Discovery Rate (FDR). Conjugated with the Analysis of Similarities (ANOSIM), the reliability of DMM clustering was further validated and then visualized by the Nonmetric multidimensional scaling (NMDS) based on the Bray–Curtis distance under bacterial genus level. The ANOSIM statistic “R” compares the mean of ranked dissimilarities between groups to the mean of ranked dissimilarities within groups. An R value close to “1.0” indicates dissimilarity between groups, whereas an R value close to “0” indicates an even distribution of high and low ranks within and between groups”. The ANOSIM statistic R always ranges between −1 to 1. The positive R values closer to 1 suggest more similarity within sites than between sites, and that close to 0 represent no difference between sites or within sites^59^. ANOSIM *p* values that are lower than 0.05 imply a higher similarity within sites. Richness (Observed OTUs/ASVs) and Pielou’s evenness for each community type were calculated for estimating the difference of alpha-diversity. The analyses of alpha-diversity, NMDS and ANOSIM were performed using R package “vegan” v2.5-6. Dynamic change of community types was showed according to collected dates of specimens with ‘pheatmap’ package in R. Furthermore, to compensate for the effects of sample size, the Margalef’s index was calculated by dividing the number of species in a sample by the natural log of the number of organisms collected^15^. For association between community types and potential confounding factors such as sex, age, virus existence, and antibiotic use, the fisher exact test based on sample count was performed and the association with FDR-corrected *p* value < 0.05 was considered significant.

### Indicator analysis in throat and gut community types

According to the definition given by the United Nations Environment Programme (1996), the indicator species are a group of species whose status provides information on the overall condition of the ecosystem and of other species in that ecosystem, reflecting the quality and changes in environmental conditions as well as aspects of community composition. To obtain the reliable indicator genus that is specific to each community type, we performed the Indicator Species Analysis using the indicspecies package (ver.1.7.8)^60^ in R platform with top 30 genus contributing to DMM clustering in both throat (accounting for 66% cumulative difference) and gut (68% cumulative difference). Dynamic changes of indicator genera corresponding to each throat community type were showed in all COVID-19 patients using the pheatmap package in R and only gut indicator genera with indicator values that were above 0.05 were presented in the patients.

### Co-occurrence network analysis of a crosstalk between throat and gut microbiota

Based on microbial genus abundances normalized by the centered log ratio transformation of both throat and gut samples collected from 13 COVID-19 patients at the same time point, we calculated the Pearson Correlation Coefficient (Pearson’s r) among the throat and gut microbial genera. The Pearson’s r with *P* values that were below 0.05 after the FDR adjustment were considered significant correlations. Co-occurrence network of significantly correlated microbial genus pairs was visualized using Cytoscape v3.8.0^61^.

### Statistics and reproducibility

Raw sequences were analyzed on Linux (Red Hat 4.8.5-36) and Windows10 environment. Software under Linux environment include USEARCH11, FASTX-Toolkit, DADA2 and Deblur, both of which were integrated in Qiime2 (v2019.10) and RDP Naive Bayesian Classifier algorithm. Software under Windows10 including Dirichlet multinomial mixtures integrated in Mothur v1.44.1, RStudio v1.2.1335. Data analysis and plotting were performed in RStudio with R v3.6.1 and R packages including pheatmap (v1.0.12), vegan (v2.5-6), permute (v0.9-5), lattice (v0.20-38), ggplot2 (v3.3.0), RColorBrewer (v1.1-2), viridis (v0.5.1), indicspecies (v 1.7.9), ade4 (v 1.7-15), ggalluvial (v 0.11.3), and grid. To promote reproducibility, we provided the analyses scripts/code of the correlation analysis R package as supplemental file 1. Detailed information on statistics and comparisons are provided in Method and/or figure legends.

### Reporting summary

Further information on research design is available in the Nature Research Reporting Summary linked to this article.

## Supplementary information

Supplementary Information

Supplementary Data 1

Supplemental Data 2

Supplementary Data 3

Supplementary Data 4

Supplementary Code

Reporting Summary

Description of Additional Supplementary Files

Peer Review File

## Data Availability

The raw data of 16 S rRNA gene sequences are available at NCBI Sequence Read Archive (SRA) (https://www.ncbi.nlm.nih.gov/sra/) at BioProject ID PRJNA639286.

## References

[1] Zhu N (2020). A novel coronavirus from patients with pneumonia in China, 2019. N. Engl. J. Med..

[2] Zhou P (2020). A pneumonia outbreak associated with a new coronavirus of probable bat origin. Nature.

[3] Wu ZY, McGoogan JM (2020). Characteristics of and important lessons from the Coronavirus Disease 2019 (COVID-19) outbreak in China smmary of a report of 72 314 cases from the Chinese Center for Disease Control and Prevention. JAMA.

[4] Guan W (2020). Clinical characteristics of Coronavirus Disease 2019 in China. N. Engl. J. Med..

[5] Honda K, Littman DR (2016). The microbiota in adaptive immune homeostasis and disease. Nature.

[6] Dubourg G, Edouard S, Raoult D (2019). Relationship between nasopharyngeal microbiota and patient’s susceptibility to viral infection. Expert. Rev. Anti Infect. Ther..

[7] Man WH, Piters WAAD, Bogaert D (2017). The microbiota of the respiratory tract: gatekeeper to respiratory health. Nat. Rev. Microbiol..

[8] Wen Z (2018). Distinct nasopharyngeal and oropharyngeal microbiota of children with Influenza A virus compared with healthy children. Biomed. Res. Int..

[9] Allen EK (2014). Characterization of the nasopharyngeal microbiota in health and during rhinovirus challenge. Microbiome.

[10] de Steenhuijsen Piters WA (2016). Nasopharyngeal microbiota, host transcriptome, and disease severity in children with respiratory syncytial virus infection. Am. J. Respir. Crit. Care Med..

[11] Xu Y (2020). Characteristics of pediatric SARS-CoV-2 infection and potential evidence for persistent fecal viral shedding. Nat. Med..

[12] Wu YJ (2020). Prolonged presence of SARS-CoV-2 viral RNA in faecal samples. Lancet Gastroenterol. Hepatol.

[13] Zuo T (2020). Alterations in gut microbiota of patients with COVID-19 during time of hospitalization. Gastroenterology.

[14] Gu, S. et al. Alterations of the gut microbiota in patients with COVID-19 or H1N1 influenza. *Clin. Infect. Dis.* **4**, ciaa709 (2020). 10.1093/cid/ciaa709 PMC7314193 32497191

[15] Clifford, H. T. & Stephenson, W. in *An Introduction to Numerical Classification* (eds Clifford, H. T. & Stephenson, W.) 158–168 (Academic Press, 1975).

[16] Burgener EB (2019). Filamentous bacteriophages are associated with chronic *Pseudomonas* lung infections and antibiotic resistance in cystic fibrosis. Sci. Transl. Med..

[17] Curran CS, Bolig T, Torabi-Parizi P (2018). Mechanisms and targeted therapies for *Pseudomonas aeruginosa* lung infection.. Am. J. Respir. Crit. Care Med..

[18] Abidi MZ, Ledeboer N, Banerjee A, Hari P (2016). Morbidity and mortality attributable to Rothia bacteremia in neutropenic and nonneutropenic patients. Diagn. Microbiol. Infect. Dis..

[19] Lee JDS, Chowdhury N, Roberts JS, Yilmaz O (2020). Host surface ectonucleotidase-CD73 and the opportunistic pathogen, Porphyromonas gingivalis, cross-modulation underlies a new homeostatic mechanism for chronic bacterial survival in human epithelial cells. Virulence.

[20] Donati C (2016). Uncovering oral Neisseria tropism and persistence using metagenomic sequencing. Nat. Microbiol..

[21] Brennan CA, Garrett WS (2019). *Fusobacterium nucleatum*—symbiont, opportunist and oncobacterium. Nat. Rev. Microbiol..

[22] Ramanan P, Barreto JN, Osmon DR, Tosh PK (2014). Rothia Bacteremia: a 10-year experience at Mayo Clinic, Rochester, Minnesota. J. Clin. Microbiol..

[23] Tanaka C (2018). A lytic bacteriophage for controlling Pseudomonas lactis in raw cow’s milk. Appl. Environ. Microbiol.

[24] La Rosa SL (2019). The human gut *Firmicute Roseburia* intestinalis is a primary degrader of dietary beta-mannans. Nat. Commun..

[25] Ozato N (2019). Blautia genus associated with visceral fat accumulation in adults 20-76 years of age. npj Biofilms Microme.

[26] Valles-Colomer M (2019). The neuroactive potential of the human gut microbiota in quality of life and depression. Nat. Microbiol.

[27] Lopez-Siles M, Duncan SH, Garcia-Gil LJ, Martinez-Medina M (2017). Faecalibacterium prausnitzii: from microbiology to diagnostics and prognostics. ISME J..

[28] Neumann A, Bjorck L, Frick IM (2020). *Finegoldia magna*, an anaerobic Gram-positive bacterium of the normal human microbiota, induces inflammation by activating neutrophils. Front. Microbiol..

[29] Patterson AM (2017). Human gut symbiont *Roseburia hominis* promotes and regulates innate immunity. Front. Immunol..

[30] Hess E (2018). *Capnocytophaga canimorsus* capsular serovar and disease severity, Helsinki Hospital District, Finland, 2000-2017. Emerg. Infect. Dis..

[31] Kononen E, Wade WG (2015). Actinomyces and related organisms in human infections. Clin. Microbiol. Rev..

[32] Perlot T, Penninger JM (2013). ACE2-from the renin-angiotensin system to gut microbiota and malnutrition. Microbes Infect..

[33] Huang C (2020). Clinical features of patients infected with 2019 novel coronavirus in Wuhan, China. Lancet.

[34] Dhar D, Mohanty A (2020). Gut microbiota and Covid-19- possible link and implications. Virus Res..

[35] Wypych TP, Wickramasinghe LC, Marsland BJ (2019). The influence of the microbiome on respiratory health. Nat. Immunol..

[36] Dang AT, Marsland BJ (2019). Microbes, metabolites, and the gut-lung axis. Mucosal Immunol..

[37] Cao W, Li TS (2020). COVID-19: towards understanding of pathogenesis. Cell Res..

[38] Rooks MG, Garrett WS (2016). Gut microbiota, metabolites and host immunity. Nat. Rev. Immunol..

[39] Seifert HS (2019). Location, location, location-commensalism, damage and evolution of the pathogenic Neisseria. J. Mol. Biol..

[40] Zhang D, Frenette PS (2019). Cross talk between neutrophils and the microbiota. Blood.

[41] Esaiassen E (2017). *Bifidobacterium Bacteremia*: clinical characteristics and a genomic approach to assess pathogenicity. J. Clin. Microbiol..

[42] Ai JW, Li Y, Zhou X, Zhang WH (2020). COVID-19: treating and managing severe cases. Cell Res..

[43] Lu R (2020). Epidemiological and clinical characteristics of COVID-19 patients in Nantong, China. J. Infect. Dev. Ctries.

[44] D’Amore R (2016). A comprehensive benchmarking study of protocols and sequencing platforms for 16S rRNA community profiling. BMC Genomics.

[45] de Muinck EJ, Trosvik P, Gilfillan GD, Hov JR, Sundaram AYM (2017). A novel ultra high-throughput 16S rRNA gene amplicon sequencing library preparation method for the Illumina HiSeq platform. Microbiome.

[46] Edgar RC (2013). UPARSE: highly accurate OTU sequences from microbial amplicon reads. Nat. Methods.

[47] Hannon, G. J. *FASTX-Toolkit*. http://hannonlab.cshl.edu/fastx_toolkit (2010).

[48] Knight R (2018). Best practices for analysing microbiomes. Nat. Rev. Microbiol..

[49] Callahan BJ (2016). DADA2: High-resolution sample inference from Illumina amplicon data. Nat. Methods.

[50] Amir A (2017). Deblur rapidly resolves single-nucleotide community sequence patterns. Msystems.

[51] Caporaso JG (2010). QIIME allows analysis of high-throughput community sequencing data. Nat. Methods.

[52] Wang Q, Garrity GM, Tiedje JM, Cole JR (2007). Naïve Bayesian classifier for rapid assignment of rRNA sequences into the new bacterial taxonomy. Appl. Environ. Microbiol..

[53] Cole JR (2013). Ribosomal Database Project: data and tools for high throughput rRNA analysis. Nucleic Acids Res..

[54] Holmes I, Harris K, Quince C (2012). Dirichlet multinomial mixtures: generative models for microbial metagenomics. PLoS ONE.

[55] Stewart CJ (2018). Temporal development of the gut microbiome in early childhood from the TEDDY study. Nature.

[56] Ding T, Schloss PD (2014). Dynamics and associations of microbial community types across the human body. Nature.

[57] Fujimura KE (2016). Neonatal gut microbiota associates with childhood multisensitized atopy and T cell differentiation. Nat. Med..

[58] Schloss PD (2009). Introducing mothur: open-source, platform-independent, community-supported software for describing and comparing microbial communities. Appl. Environ. Microbiol..

[59] Clarke KR (1993). Non-parametric multivariate analyses of changes in community structure. Aust. J. Ecol..

[60] De Cáceres M, Legendre P, Moretti M (2010). Improving indicator species analysis by combining groups of sites. Oikos.

[61] Otasek D, Morris JH, Boucas J, Pico AR, Demchak B (2019). Cytoscape Automation: empowering workflow-based network analysis. Genome Biol..

