# Supplementary material for: Temporal association between human upper respiratory and gut bacterial microbiomes during the course of COVID-19 in adults
Source: Commun Biol. 2021 Feb 18;4:240. doi: 10.1038/s42003-021-01796-w (PMC7893062; doi:10.1038/s42003-021-01796-w)
Supplement: Supplementary file 1 — Supplementary Information [file 42003_2021_1796_MOESM1_ESM.pdf]

Supplementary materials

Temporal association between human upper respiratory and gut bacterial microbiomes during the course of COVID-19 in adults

Running title: Upper respiratory and gut microbiomes in COVID-19 adults

Rong Xu[1, 4, #], Renfei Lu[2, #], Tao Zhang[3, #], Qunfu Wu[3, #], Weihua Cai[2], Xudong Han[2], Zhenzhou Wan[5], Xia Jin[1] *, Zhigang Zhang[3] *, Chiyu Zhang[1,4] *

1. Shanghai Public Health Clinical Center, Fudan University, Shanghai 201508, China
2. Clinical Laboratory, Nantong Third Hospital Affiliated to Nantong University, Nantong 226006, China
3. State Key Laboratory for Conservation and Utilization of Bio-Resources in Yunnan, School of Life Sciences, Yunnan University, Kunming, Yunnan 650091, China
4. Pathogen Discovery and Evolution Unit, Institut Pasteur of Shanghai, Chinese Academy of Sciences, Shanghai 200031, China.
5. Medical Laboratory of Taizhou Fourth People's Hospital, Taizhou 225300 China

# These authors contributed equally to this work.

* Correspondence authors: Chiyu Zhang, Shanghai Public Health Clinical Center, Fudan University, Shanghai 201508, China. Email: zhangcy1999@hotmail.com
Or Zhigang Zhang, State Key Laboratory for Conservation and Utilization of Bio-Resources in Yunnan, School of Life Sciences, Yunnan University, No. 2 North Cuihu Road, Kunming, Yunnan 650091, China. Email: zhangzhigang@ynu.edu.cn
Or Xia Jin, Shanghai Public Health Clinical Center, Fudan University, Shanghai 201508, China. Email: jinxia@shphc.org.cn

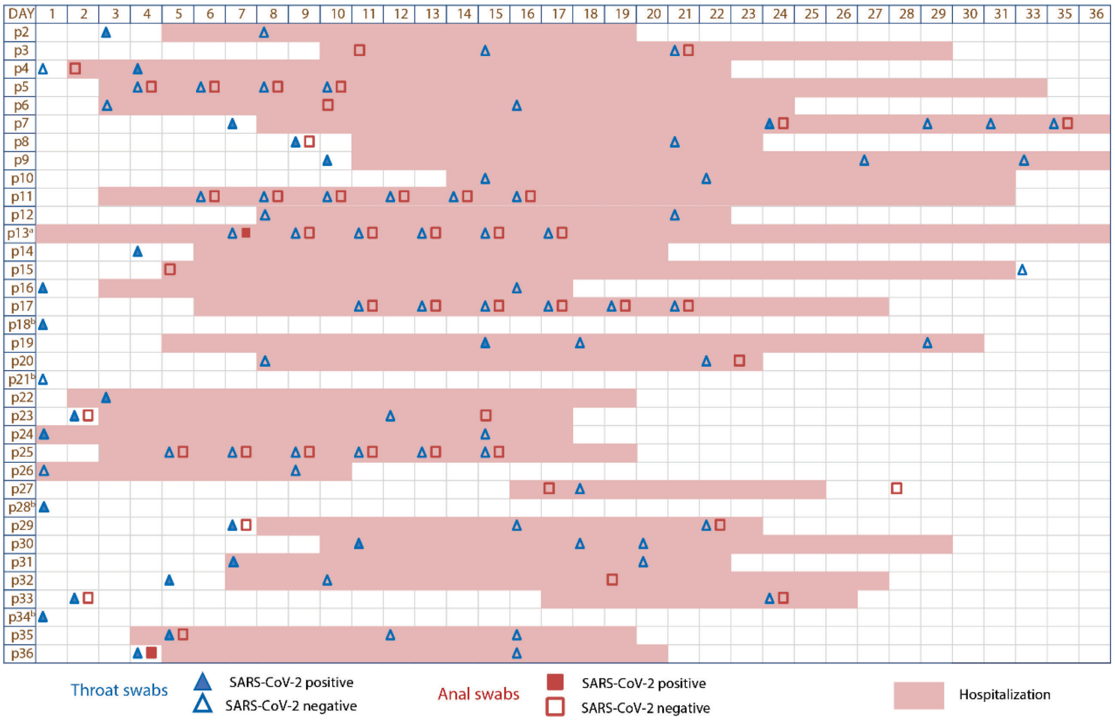

Supplementary figure S1. COIVD-19 patient admission and discharge time as well as the point of detection of SARS-CoV-2. a. the hospitalization of p13 was 40 days. b. the information of these patients was unavailable. DAY 1 was the day of symptom onset. Some COVID-19 patients were initially found/confirmed elsewhere and their samples were unavailable for this study. A lack of positive samples in some patients (e.g. P05, P11 and P25) were due to the unavailability of early positive samples.

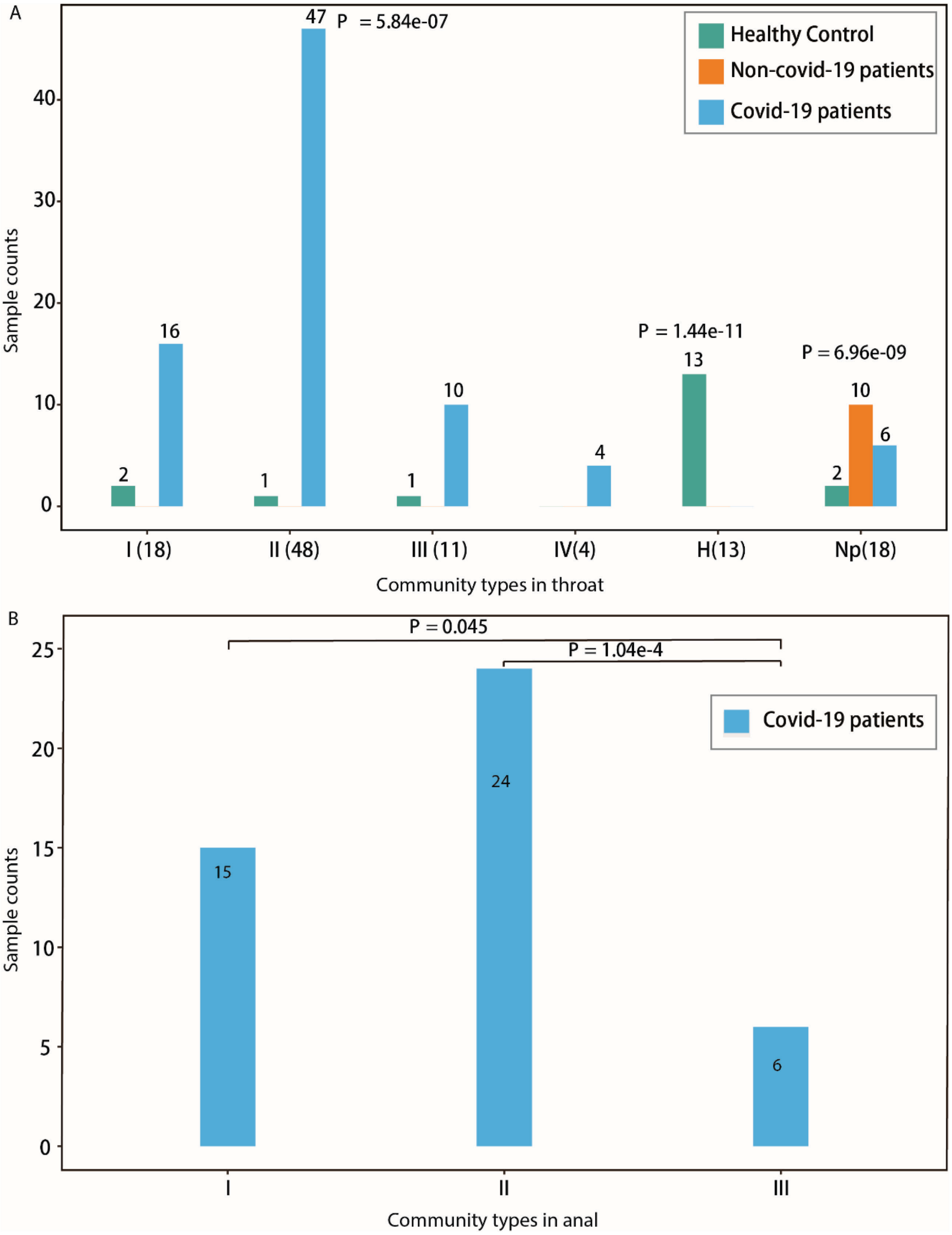

Supplementary figure S2. Group distribution characteristics of each community cluster. Total number is shown in parentheses.

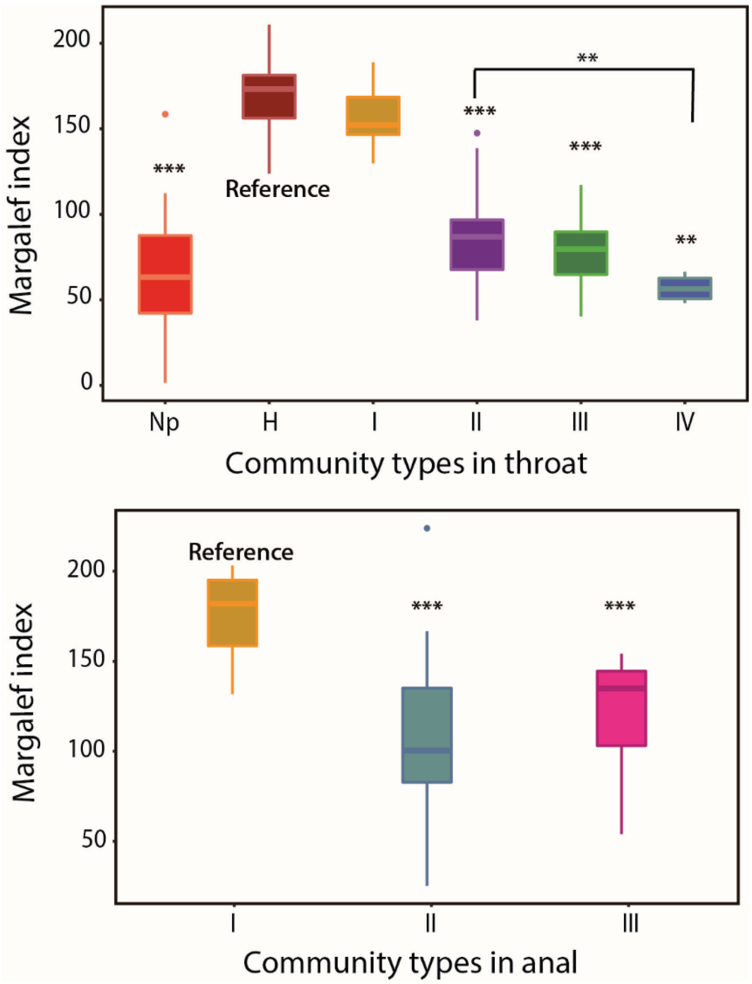

Supplementary figure S3. Margalef index of each community cluster. Margalef's index of clusters showed similar trends with richness as other analyses of Richness in both throat and anal samples. Margalef's index for each sample is calculated with the number of species (n) in sample minus 1 divided by the natural logarithm of the total sample count of cluster (N) in which the sample was included. Margalef's index = (n-1) / ln(N).

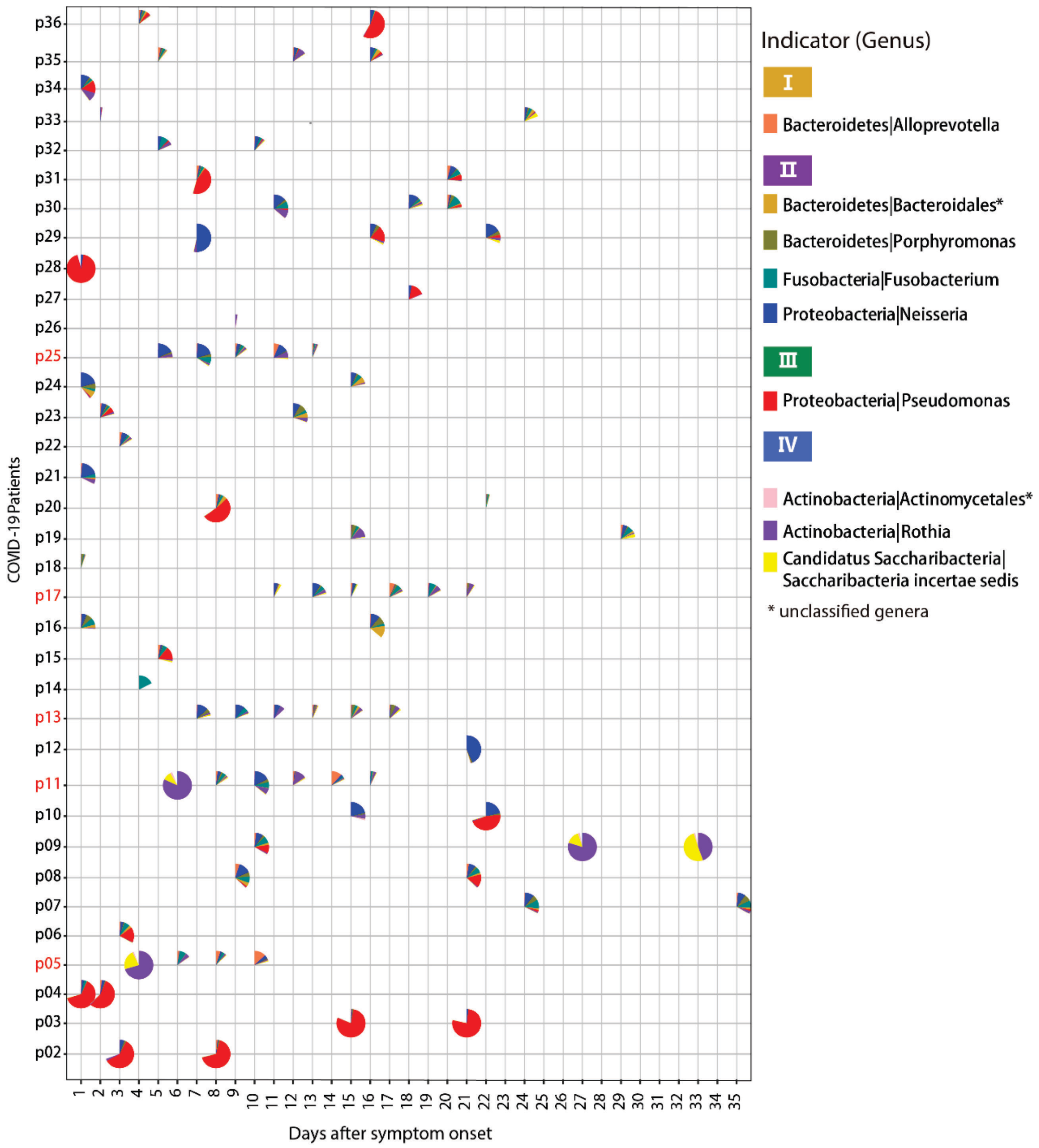

Supplementary figure S4. Time-scale changes of indicators of throat microbial community types. Color sectors represent relative abundance of indicators in different COVID-19 stages. Linked to **Figure 1**.

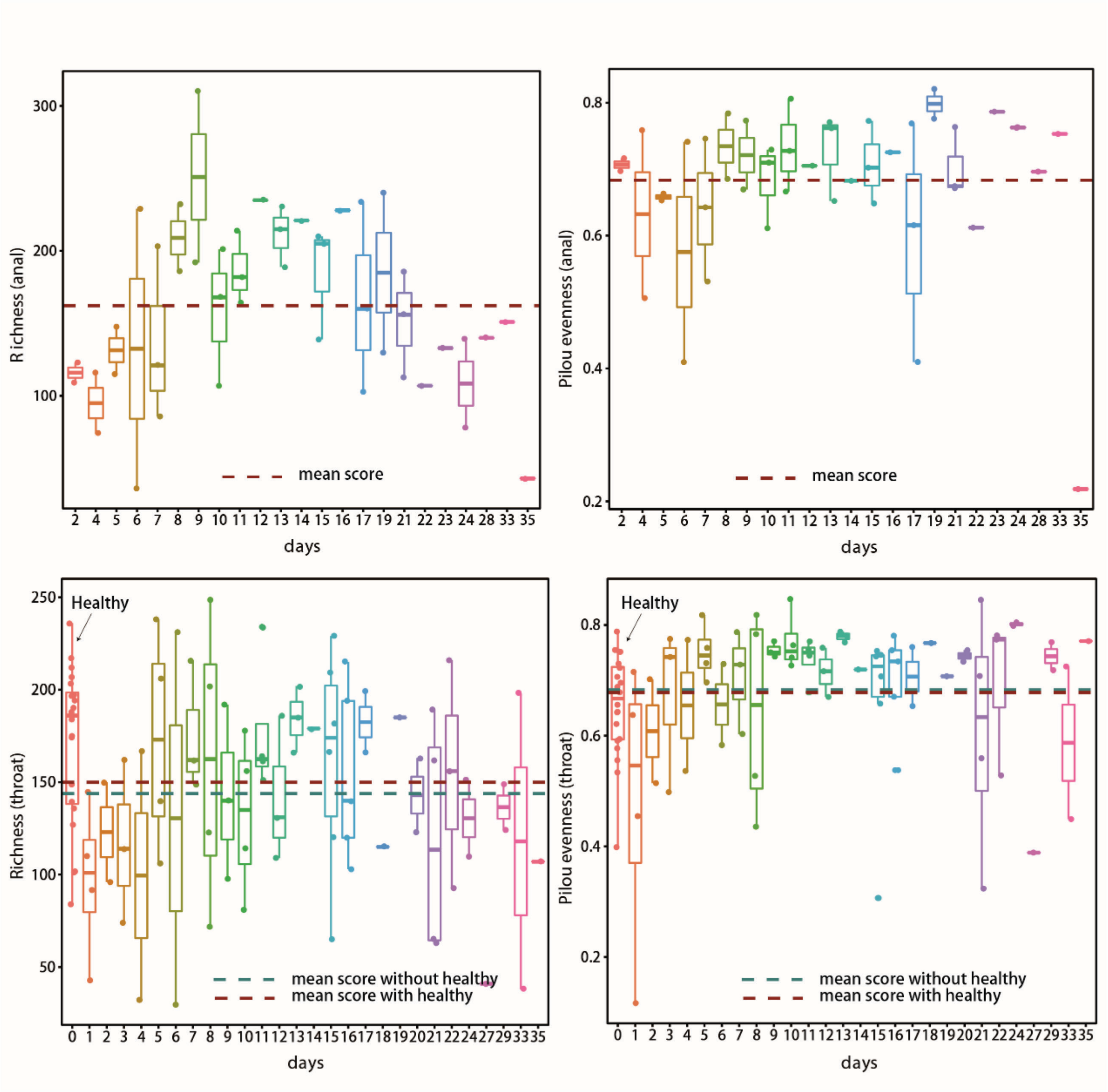

Supplementary figure S5. Dynamic changes of alpha-diversity since appearance of symptoms in COVID-19 patients.

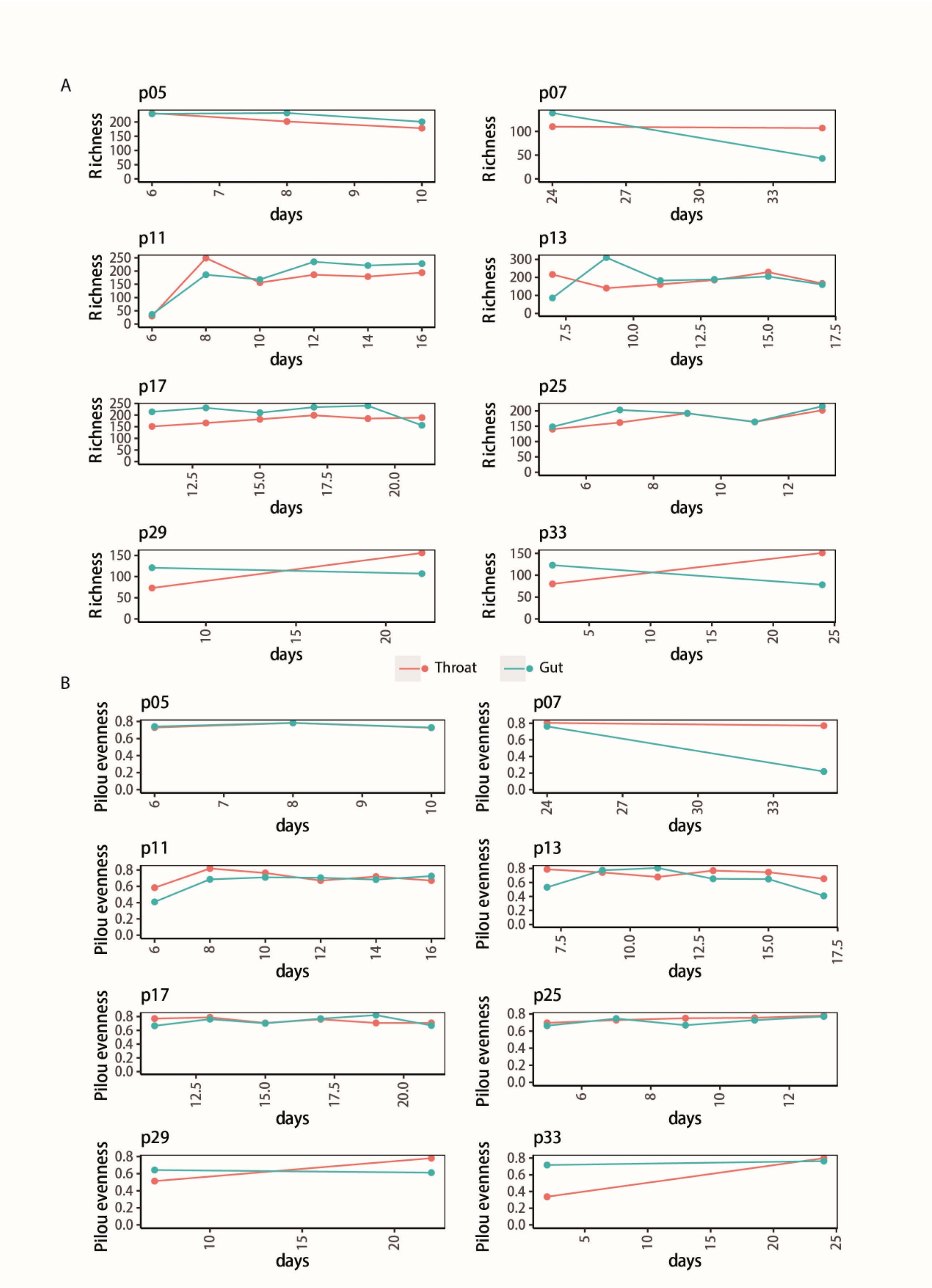

Supplementary figure S6. Dynamic changes of microbiome diversity over time during COVID-19 in eight patients. a) Richness, b) Peilou evenness.

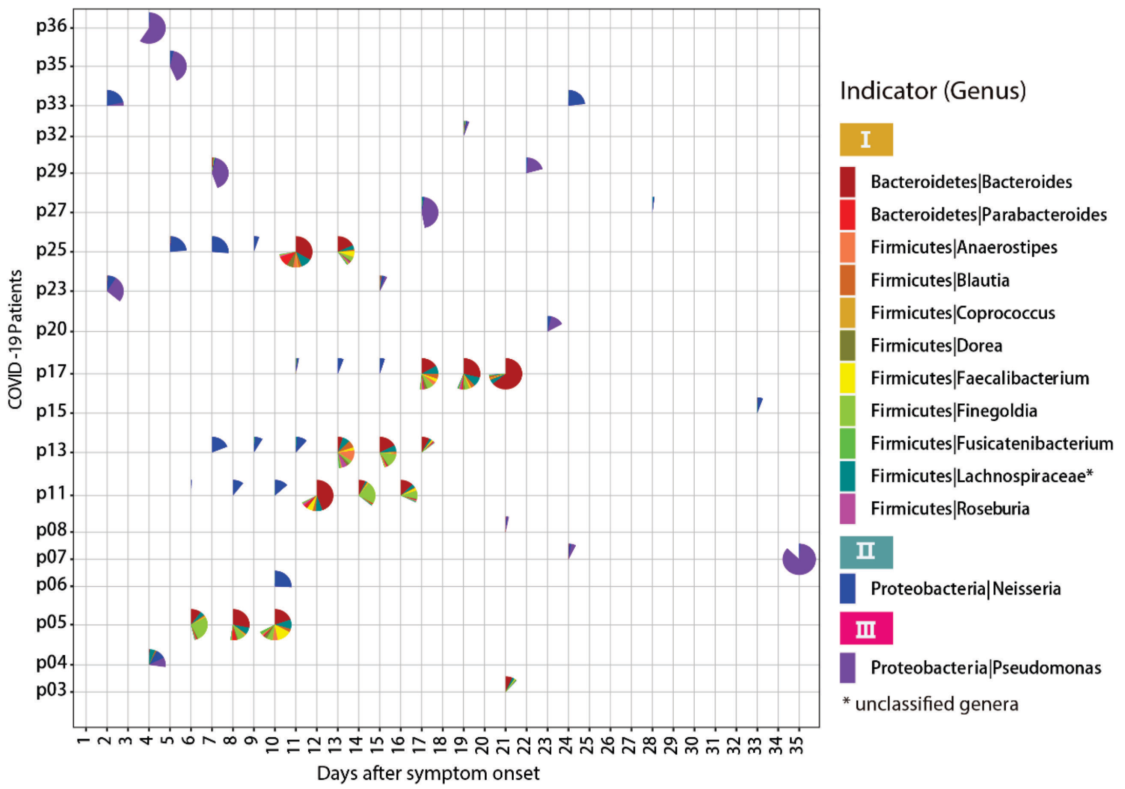

Supplementary Figure S7. Time-scale changes of indicators of gut microbial community types. Color sectors represent relative abundance of indicators in different COVID-19 stages. Linked to **Figure 2**.

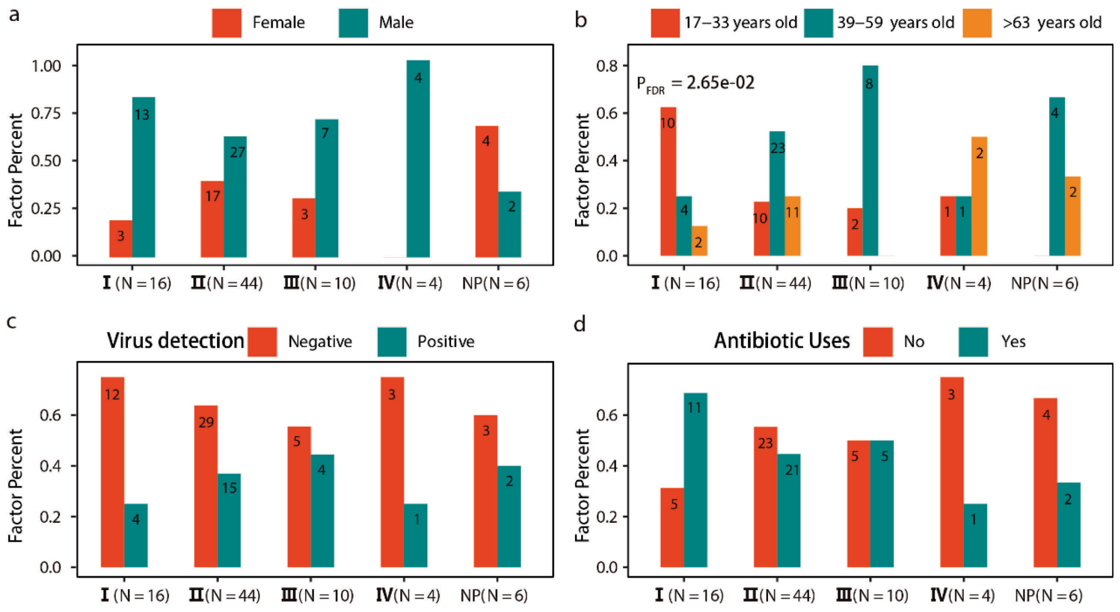

Supplementary Figure S8. Enrichment analysis of impact factors on throat microbial community typing. a) Sex, b) Age, c) Virus detection, and d) Antibiotic uses. Enrichment analysis was performed by using the Fisher's exact test under FDR-adjusted P < 0.05. Sample numbers were shown on the bar. Only COVID-19 patients were used for this analysis.

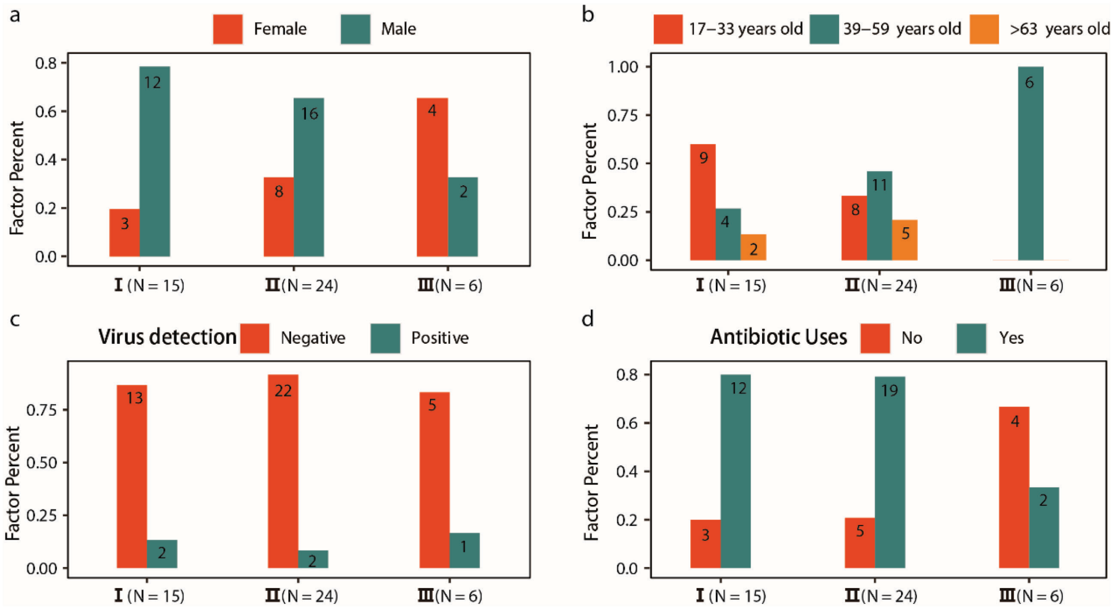

Supplementary Figure S9. Enrichment analysis of impact factors on gut microbial community typing. a) Sex, b) Age, c) Virus detection, and d) Antibiotic uses. Enrichment analysis was performed by using the Fisher's exact test under FDR-adjusted $P < 0.05$. No significant enrichment was observed. Sample numbers were shown on the bar.

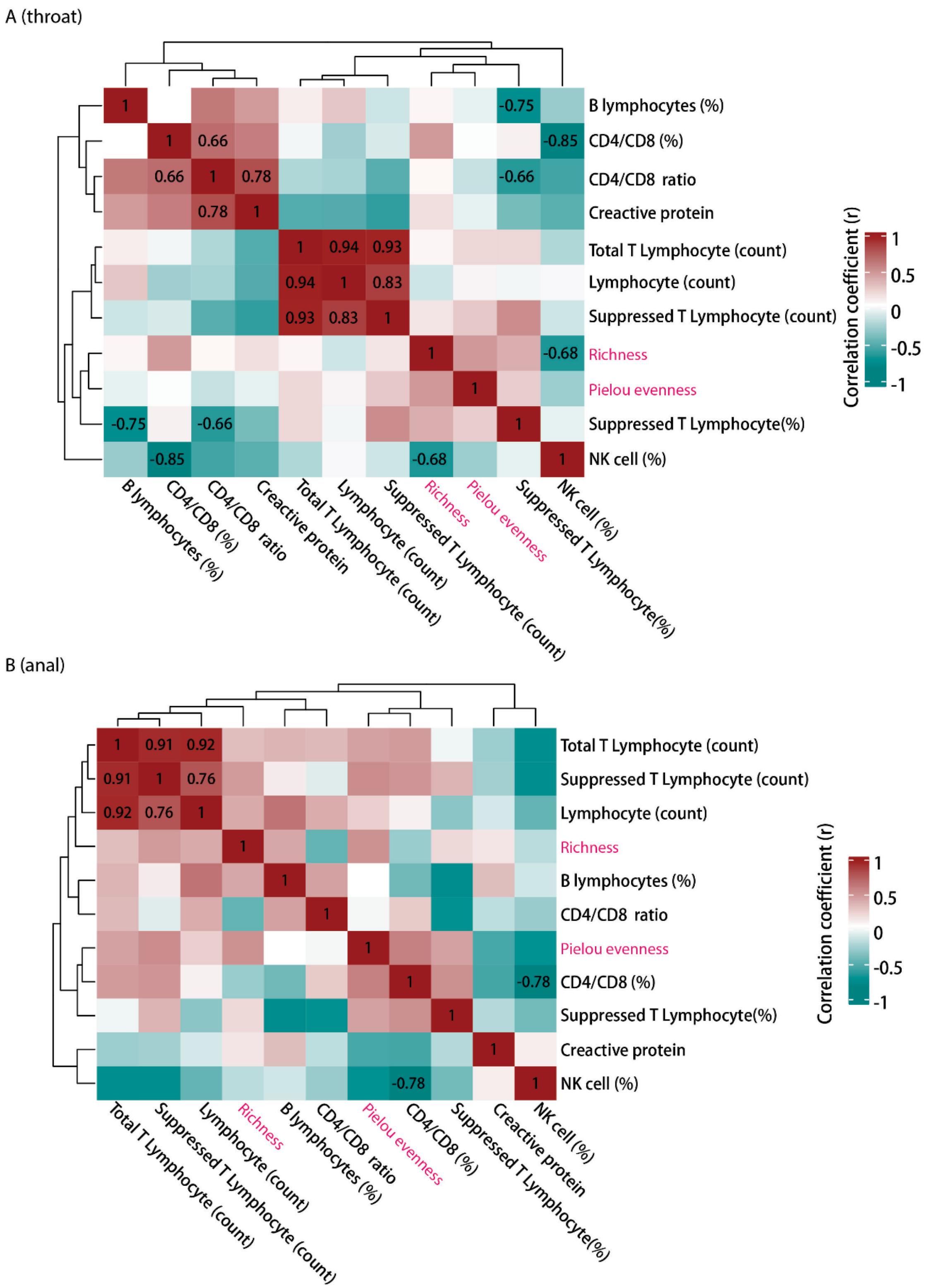

Supplementary figure S10. Correlation of microbiome diversity with clinical parameters. a) Correlation between rectum microbiota diversity index and clinical parameters (Correlation coefficient (r) were marked in cells for correlation pairs with p<0.05). b) Correlation between Throat swab microbiota diversity index and clinical parameters (Correlation coefficient (r) were marked in

cells for correlation pairs with $p<0.05$).

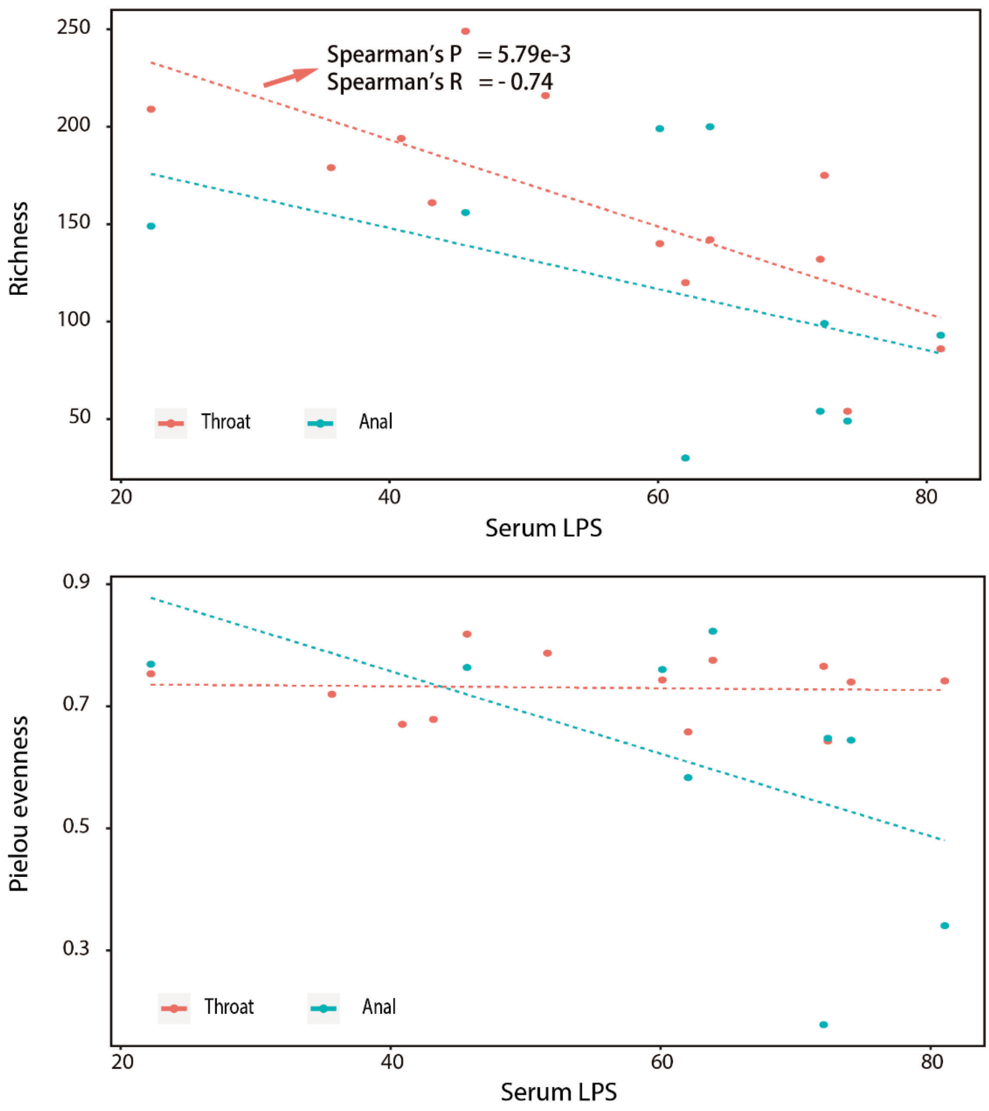

Supplementary figure S11. Correlation between microbiome diversity and LPS levels. a) Richness, b) Peilou evenness.

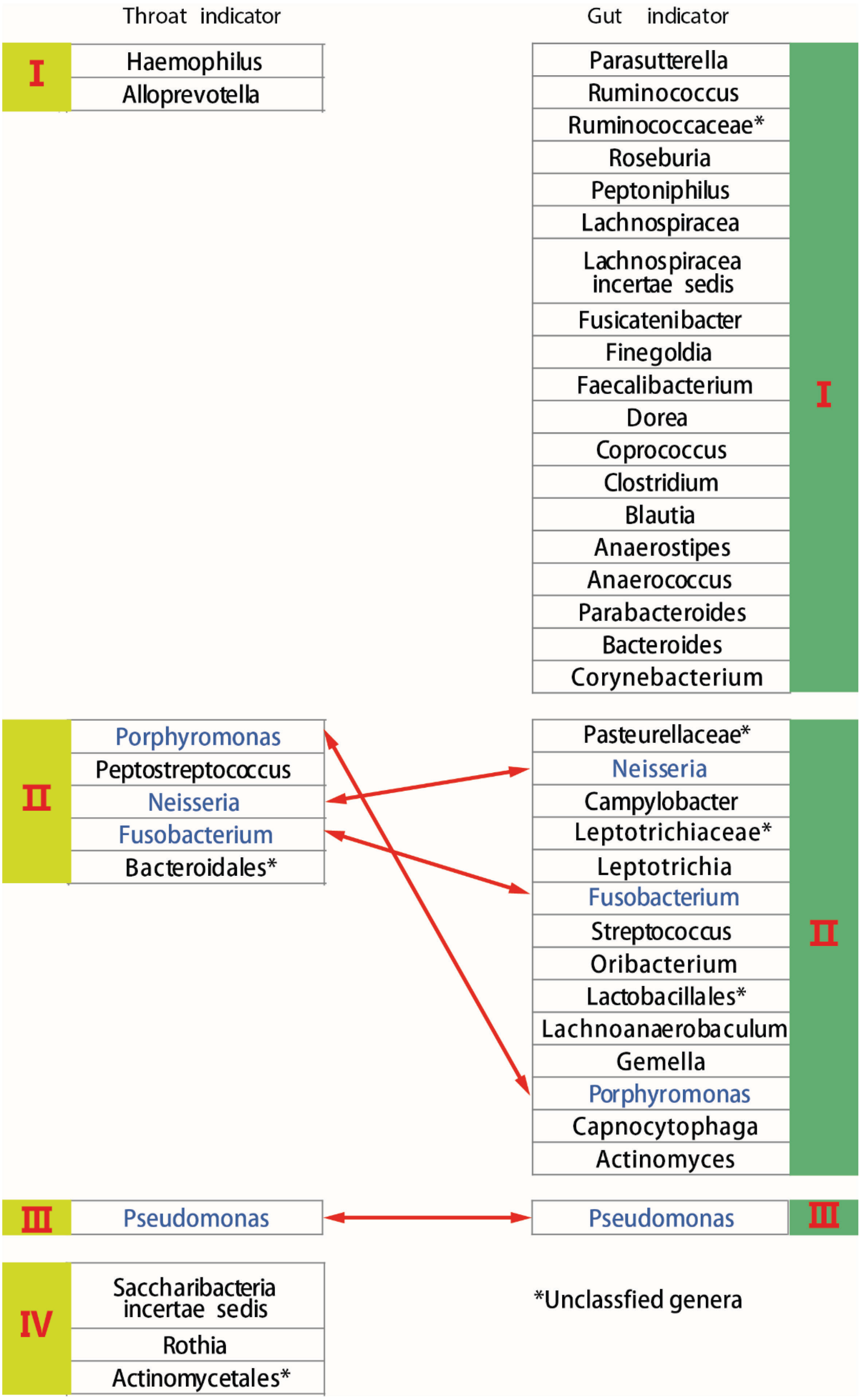

Supplementary Figure S12. Comparisons of indicator genera between throat and gut microbial clusters. Shared indicator genera are highlighted by red arrows.

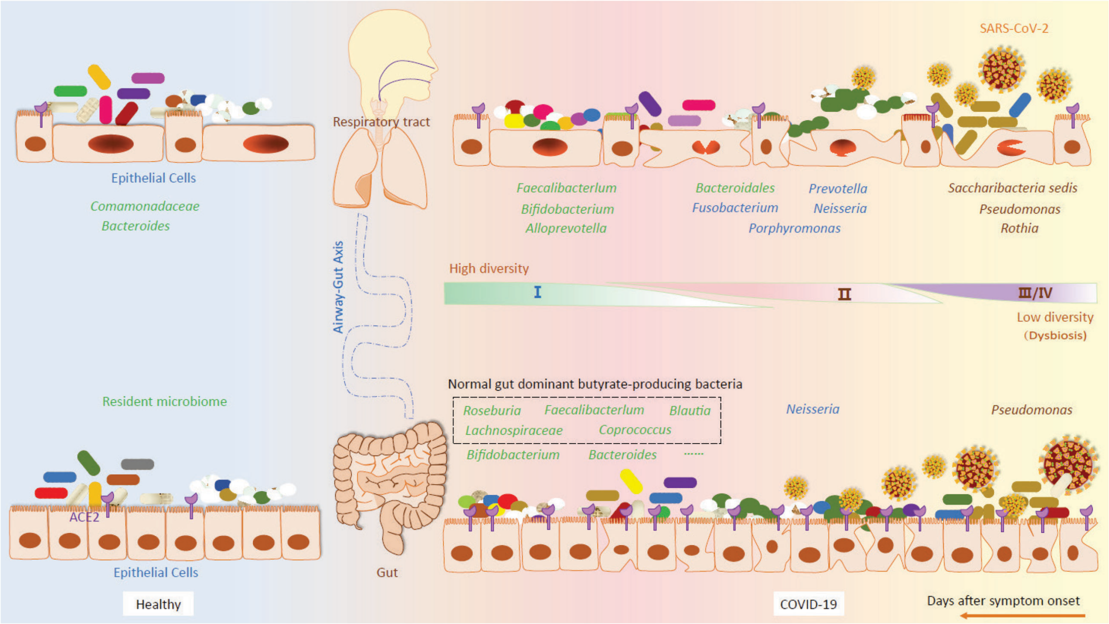

Supplementary Figure S13. Putative restoration model of the respiratory and gut microbiomes over time in adults with mild COVID-19. SARS-CoV-2 infection resulted in a fast dysbiosis in both the respiratory tract and gut at the very early phase of the disease. A fast restoration of both the respiratory and gut microbiomes from early dysbiosis towards late more diverse status was observed in most adults with mild COVID-19 albeit they seemed to have a relatively slow clinical recovery. This model reflects the major microbiome dynamic change in most adults with mild COVID-19 but not all features in all patients, especially in those with severe disease.

Supplemental table S1. The LPS level of the serum of patients.

Patients	Day	LPS level
P03	15	<5
P05	4	<5
	6	72.39
	8	63.87
	10	45.66
P07	29	<5
	31	<5
	35	<5
P09	10	201.4
	27	35.65
	33	<5
	34	<5
P10	15	43.17
	22	40.87
P36	4	<5
	16	<5
P35	5	<5
	12	<5
	16	<5
P11	6	<5
	8	<5
	10	<5
	12	<5
	14	<5
	16	<5
P13	7	<5
	13	<5
	15	81.05
	17	62.04
P17	11	<5
	19	72.08
P25	5	<5
	7	74.11+
P29	7	<5
	16	51.62
	22	<5
P33	24	<5
P23	2	22.24
P08	9	<5
	21	60.13

Supplementary table S2. Dynamic changes of clinical parameters of 13 COVID-19 patients.

Patents Number	Detection Data after Symptom Onset [i]	Total T lymphocyte count/μL	Percentage of CD4/CD8 T lymphocytes/%	CD4/CD8 T lymphocyte ratio	Percentage of suppressed T lymphocytes/CTL/%	Suppressed T lymphocytes/CTL count/μL	Percentage of NK cell/%	Percentage of B lymphocytes/%	Lymphocyte count/μL	C reactive protein/mg/L
P02	Day 11	1089	48.33	2.3	21	292	8.3	13.63	1430	8.5
P03	Day 11	674	39.8	1.27	31.23	280	22.67	7.37	960	0.7
	Day 14	440	23.87	0.85	28.17	221	25.17	20.7	829	<0.4
	Day 17	1069	49.1	1.8	27.33	378	5.3	16.97	1396	<0.5
	Day 21	1940	54.83	1.78	30.77	676	8.57	6.3	2271	<0.5
P05	Day 3	1383	18.23	0.63	29.07	661	26.9	12.37	2328	5.68
	Day 9	2146	20.43	0.65	31.5	1000	23.47	11.57	3371	10
P07	Day 8	527	40.40	1.82	22.20	171	14.43	19.63	814	
	Day 16	592	39.57	1.62	24.40	213	9.00	24.03	901	
	Day 21	1041	42.77	1.72	24.93	355	8.17	21.80	1502	
	Day 23	1057	47.23	2.38	19.87	274	6.97	21.30	1499	
	Day 25	859	46.47	2.02	23.00	255	6.17	20.77	1189	38.8
	Day 31	967	44.93	1.69	26.60	340	9.13	17.63	1320	
P09	Day 12	288	34.93	1.44	24.27	121	22.57	19.40	497	50.3
	Day 19	635	49.73	2.49	20.00	180	8.10	24.47	946	12.2
	Day 23	508	47.07	1.79	26.23	184	12.20	16.37	715	
	Day 28	898	56.77	2.56	22.20	240	7.50	10.97	1146	<0.5
	Day 33	1021	51.07	2.16	22.27	305	11.57	13.53	1491	13.63
P10	Day 15	554	56.90	3.80	14.97	112	10.07	16.63	757	
	Day 20	863	55.13	2.71	20.37	222	7.40	16.37	1136	
P11	Day 8	1242	43.23	1.20	36.00	530	14.53	6.00	1565	3.88

	Day 15	1238	40.53	1.17	34.70	528	13.73	8.47	1604	1.30
P13	Day 9	1757	39.97	1.81	22.07	571	21.30	15.73	2731	1.75
	Day 16	1801	44.13	2.16	20.40	525	8.07	23.67	2684	1.73
P17	Day 13	2110	36.30	1.40	25.93	760	12.30	19.30	3114	2.76
	Day 20	2777	35.43	1.26	28.20	1105	14.77	16.47	4059	1.55
P25	Day 5	1354	42.23	1.94	21.73	420	11.27	18.23	1972	4.02
	Day 9	1500	38.27	2.15	17.80	388	18.63	15.53	2339	2.46
	Day 13	1139	30.23	1.49	20.33	385	29.00	11.80	1946	4.04
P29	Day 9	320	18.47	0.59	31.07	185	20.00	23.57	622	
P33	Day 19	357	40.47	1.58	25.53	127	16.73	14.90	534	<0.3
	Day 24	641	34.53	1.28	26.90	264	27.90	9.00	1026	1.31
P36	Day 6	584	46.33	2.16	21.50	171	15.57	8.83	821	
	Day 9	242	27.33	1.47	18.60	87	34.70	13.37	489	
	Day 11	314	23.37	1.41	16.53	121	25.27	27.77	751	<0.499
	Day 16	822	56.13	3.12	18.00	193	4.70	20.47	1106	18.6

[i] the Day 1 was the time point disease onset, the first date of each patients in this table was the first day of hospitalization.
