# Supplementary material for: Temporal association between human upper respiratory and gut bacterial microbiomes during the course of COVID-19 in adults
Source: Commun Biol. 2021 Feb 18;4:240. doi: 10.1038/s42003-021-01796-w (PMC7893062; doi:10.1038/s42003-021-01796-w)
Supplement: Supplementary file 6 — Supplementary Code [file 42003_2021_1796_MOESM6_ESM.docx]

setwd('D:\\sh-Bastder\\work\\OTU_table')

library(compositions)

f1=read.table('airway-sample.txt',header=F)

f2=read.table('gut-sample.txt',header=F)

s1=as.character(f1[,1])

s11=paste(f1[,3],f1[,2],sep='-')

s111=paste(f1[,3],f1[,2],sep='-')

names(s1)=s11

names(s11)=s1

s2=as.character(f2[,1])

s22=paste(f2[,3],f2[,2],sep='-')

s222=paste(f2[,3],f2[,2],sep='-')

names(s2)=s22

names(s22)=s2

s12=s11[s11%in%s22]

s21=s22[s22%in%s11]

airsamp=as.vector(s1[s12])

gutsamp=as.vector(s2[s21])

f11=read.table('throat_swab\\DMM\\otu-shared-dmm-input-genus-throat_swab.1.mothur\\otu-shared-dmm-input-genus-throat_swab.1.1.days.names.txt',header=T,stringsAsFactors = F,sep='\t')

f22=read.table('anal_swab\\otu-shared-dmm-input-genus.1.dmm.mothur\\otu-shared-dmm-input-genus.1.1.days.names.txt',header=T,stringsAsFactors = F,sep='\t')

f3=f11[,7:ncol(f11)]

rownames(f3)=f11[,1]

f4=f22[,7:ncol(f22)]

rownames(f4)=f22[,1]

s3=rownames(f3)[rownames(f3)%in%airsamp]

f31=f3[s3,]/3000

rownames(f31)=s11[s3]

colnames(f31)=paste('air.',colnames(f31),sep='')

s4=rownames(f4)[rownames(f4)%in%gutsamp]

f41=f4[s4,]/4700

rownames(f41)=s22[s4]

colnames(f41)=paste('gut.',colnames(f41),sep='')

f32=f31[rownames(f31)%in%rownames(f41),]

f42=f41[rownames(f41)%in%rownames(f31),]

s1[rownames(f32)]#air

s2[rownames(f42)]#gut

fr=read.table('richness.txt',header=F,stringsAsFactors = F)

ra=fr[fr$V4=="a",][,2]

names(ra)=fr[fr$V4=="a",][,1]

rg=fr[fr$V4=="g",][,2]

names(rg)=fr[fr$V4=="g",][,1]

richa=ra[names(ra)[names(ra)%in%s1[rownames(f32)]]]

names(richa)

richg=rg[names(rg)[names(rg)%in%s2[rownames(f42)]]]

cor.test(richa,richg,method = 'spearman')

fp=read.table('pielou.txt',header=F,stringsAsFactors = F)

pa=fp[fp$V4=="a",][,2]

names(pa)=fp[fp$V4=="a",][,1]

pg=fp[fp$V4=="g",][,2]

names(pg)=fp[fp$V4=="g",][,1]

piloua=pa[names(pa)[names(pa)%in%s1[rownames(f32)]]]

piloug=pg[names(pg)[names(pg)%in%s2[rownames(f42)]]]

cor.test(piloua,piloug,method = "spearman")

names(richa)=s11[names(richa)]

names(richg)=s22[names(richg)]

f32r=data.frame(f32)#,ald=richa[rownames(f32)])

f42g=data.frame(f42)#,ald=richg[rownames(f42)])

#clna=paste('a',colnames(f32r),sep='.')

#clng=paste('g',colnames(f42g),sep='.')

#colnames(f32r)=clna

#colnames(f42g)=clng

#rownames(clna)

tt1=t(as.data.frame(strsplit(colnames(f32r),split='\\.p__')))

rownames(tt1)=seq(1,nrow(tt1),1)

ttt1=tt1[,2]

for (i in 1:length(ttt1))

{

if (ttt1[i]=='Actinobacteria.c__Actinobacteria.Other.Other.Other')

{

ttt1[i]='Actinobacteria.c__Actinobacteriaclass.Other.Other.Other'

}

else if(ttt1[i]=='Actinobacteria.Other.Other.Other.Other')

{

ttt1[i]='Actinobacteriaphylum.Other.Other.Other.Other'

}

}

for (i in 1:length(ttt1))

{

if (strsplit(ttt1[i],split='\\.')[[1]][length(strsplit(ttt1[i],split='\\.')[[1]])]=="Other")

{

xm=paste('air',strsplit(ttt1[i],split='\\.')[[1]][1],strsplit(ttt1[i],split='_')[[1]][length(strsplit(ttt1[i],split='_')[[1]])],sep='|')

ttt1[i]=paste(gsub('.Other','',xm),'*',sep='')

}

else

{

ttt1[i]= paste('air',strsplit(ttt1[i],split='\\.')[[1]][1] ,strsplit(ttt1[i],split='g__')[[1]][length(strsplit(ttt1[i],split='g__')[[1]])],sep='|')

}

}

ttt1

colnames(f32r)=ttt1

tt11=t(as.data.frame(strsplit(colnames(f42g),split='\\.p__')))

rownames(tt11)=seq(1,nrow(tt11),1)

ttt11=tt11[,2]

for (i in 1:length(ttt11))

{

if (ttt11[i]=='Actinobacteria.c__Actinobacteria.Other.Other.Other')

{

ttt11[i]='Actinobacteria.c__Actinobacteriaclass.Other.Other.Other'

}

else if(ttt11[i]=='Actinobacteria.Other.Other.Other.Other')

{

ttt11[i]='Actinobacteriaphylum.Other.Other.Other.Other'

}

}

for (i in 1:length(ttt11))

{

if (strsplit(ttt11[i],split='\\.')[[1]][length(strsplit(ttt11[i],split='\\.')[[1]])]=="Other")

{

xm=paste('gut',strsplit(ttt11[i],split='\\.')[[1]][1],strsplit(ttt11[i],split='_')[[1]][length(strsplit(ttt11[i],split='_')[[1]])],sep='|')

ttt11[i]=paste(gsub('.Other','',xm),'*',sep='')

}

else

{

ttt11[i]= paste('gut',strsplit(ttt11[i],split='\\.')[[1]][1] ,strsplit(ttt11[i],split='g__')[[1]][length(strsplit(ttt11[i],split='g__')[[1]])],sep='|')

}

}

ttt11

colnames(f42g)=ttt11

f33=f32r[sort(rownames(f32r)),]

f33=f33[,apply(f33,2,sum)>0.001]

f43=f42g[sort(rownames(f42g)),]

f43=f43[,apply(f43,2,sum)>0.001]

f5=data.frame(f33,f43)

f5=clr(f5)# centered log ratio

d1=data.frame()

for (i in 1:(ncol(f5)-1))

{

d=data.frame()

for (j in (i+1):ncol(f5))

{

d[j,1]=colnames(f5)[i]

d[j,2]=colnames(f5)[j]

d[j,3]=cor.test(as.numeric(f5[,i]),as.numeric(f5[,j]))$estimate

d[j,4]=cor.test(f5[,i],f5[,j])$p.value

}

d1=rbind(d1,d)

}

d1

d1=na.omit(d1)

colnames(d1)=c('source','target','r-spearman','pvalue')

pa=p.adjust(d1$pvalue,n=nrow(d1))

d2=data.frame(d1,fdr=pa)

na.omit(d2)

d3=d2[d2$fdr<0.05,]

write.table(d2,'genus-correlation-gut-airway-13-patients.all.rb.clr.txt',row.names=F,col.names=T,sep='\t')

write.table(d3,'genus-correlation-gut-airway-13-patients.rb.clr.txt',row.names=F,col.names=T,sep='\t')
