# Supplementary material for: Temporal association between human upper respiratory and gut bacterial microbiomes during the course of COVID-19 in adults
Source: Commun Biol. 2021 Feb 18;4:240. doi: 10.1038/s42003-021-01796-w (PMC7893062; doi:10.1038/s42003-021-01796-w)
Supplement: Supplementary file 7 — Reporting Summary [file 42003_2021_1796_MOESM7_ESM.pdf]

nature research

Corresponding author(s): Chiyu Zhang

Last updated by author(s): Jan 9, 2021

Reporting Summary

Nature Research wishes to improve the reproducibility of the work that we publish. This form provides structure for consistency and transparency in reporting. For further information on Nature Research policies, see our Editorial Policies and the Editorial Policy Checklist.

Statistics

For all statistical analyses, confirm that the following items are present in the figure legend, table legend, main text, or Methods section.

n/a	Confirmed	
☐	☒	The exact sample size (*n*) for each experimental group/condition, given as a discrete number and unit of measurement
☐	☒	A statement on whether measurements were taken from distinct samples or whether the same sample was measured repeatedly
☐	☒	The statistical test(s) used AND whether they are one- or two-sided<br>*Only common tests should be described solely by name; describe more complex techniques in the Methods section.*
☐	☒	A description of all covariates tested
☐	☒	A description of any assumptions or corrections, such as tests of normality and adjustment for multiple comparisons
☐	☒	A full description of the statistical parameters including central tendency (e.g. means) or other basic estimates (e.g. regression coefficient) AND variation (e.g. standard deviation) or associated estimates of uncertainty (e.g. confidence intervals)
☐	☒	For null hypothesis testing, the test statistic (e.g. *F*, *t*, *r*) with confidence intervals, effect sizes, degrees of freedom and *P* value noted<br>*Give P values as exact values whenever suitable.*
☐	☒	For Bayesian analysis, information on the choice of priors and Markov chain Monte Carlo settings
☐	☒	For hierarchical and complex designs, identification of the appropriate level for tests and full reporting of outcomes
☐	☒	Estimates of effect sizes (e.g. Cohen's *d*, Pearson's *r*), indicating how they were calculated

Our web collection on statistics for biologists contains articles on many of the points above.

Software and code

Policy information about availability of computer code

Data collection	Commercial softwares were not used for data collection
Data analysis	Raw sequences were analyzed on Linux (Red Hat 4.8.5-36) and Windows10 environment. Software under Linux environment include USEARCH11, FASTX-Toolkit, DADA2 and Deblur both of which were integrated in Qiime2 (v2019.10) and RDP Naive Bayesian Classifier algorithm. Software under Windows10 including Dirichlet multinomial mixtures integrated in Mothur v1.44.1, RStudio v1.2.1335. Data analysis and plotting were performed in RStudio with R v3.6.1 and R packages including pheatmap (v1.0.12), vegan (v2.5-6), permute (v0.9-5), lattice (v0.20-38), ggplot2 (v3.3.0), RColorBrewer (v1.1-2), viridis (v0.5.1), indicspecies (v 1.7.9), ade4 (v 1.7-15), ggalluvial (v 0.11.3), grid.

For manuscripts utilizing custom algorithms or software that are central to the research but not yet described in published literature, software must be made available to editors and reviewers. We strongly encourage code deposition in a community repository (e.g. GitHub). See the Nature Research guidelines for submitting code & software for further information.

Data

Policy information about availability of data

All manuscripts must include a data availability statement. This statement should provide the following information, where applicable:

- Accession codes, unique identifiers, or web links for publicly available datasets
- A list of figures that have associated raw data
- A description of any restrictions on data availability

Sample information and raw sequences are available in the National Center for Biotechnology Information Sequence Read Archive under BioProject ID PRJNA639286.

Field-specific reporting

Please select the one below that is the best fit for your research. If you are not sure, read the appropriate sections before making your selection.

☒ Life sciences ☐ Behavioural & social sciences ☐ Ecological, evolutionary & environmental sciences

For a reference copy of the document with all sections, see nature.com/documents/nr-reporting-summary-flat.pdf

Life sciences study design

All studies must disclose on these points even when the disclosure is negative.

Sample size	A total of 58 subjects, including 35 laboratory-confirmed COVID-19 patients, 10 SARS-CoV-2 negative patients with various diseases (non-COVID-19) and 13 healthy adults were enrolled in this study. Demographic and clinical characteristics of these patients were provided in Supplementary Table S2 and S3. Specimens including throat swabs and anal swabs were collected from the patients during hospitalization (10-40) at Nantong Third Hospital Affiliated to Nantong University and from healthy adults when they visited the same hospital for physical examination. Sampling was performed using flexible, sterile, dry swabs, which can reach the posterior oropharynx and anus easily (approximately 2 inches) by the professionals at the hospital. At least two throat swabs at different days were available for 32 of 38 COVID-19 patients (Supplementary Fig S1).
Data exclusions	No available data was excluded.
Replication	For the different phases we concluded, the samples from different individuals were studied. For the dynamic changes during the course of disease, the samples collected from three body sites at different time points were studied.
Randomization	The mechanism of DMM(Dirichlet multinomial mixtures) algorithm is based on microbial composition only, which sensitivity, reliability and accuracy were widely confirmed in microbiome studies.
Blinding	Blinding was not possible in the analysis of clinical index with dynamics of microbiome.

Reporting for specific materials, systems and methods

We require information from authors about some types of materials, experimental systems and methods used in many studies. Here, indicate whether each material, system or method listed is relevant to your study. If you are not sure if a list item applies to your research, read the appropriate section before selecting a response.

Materials & experimental systems

n/a	Involved in the study	
☒	☐	Antibodies
☒	☐	Eukaryotic cell lines
☒	☐	Palaeontology and archaeology
☒	☐	Animals and other organisms
☐	☒	Human research participants
☒	☐	Clinical data
☒	☐	Dual use research of concern

Methods

n/a	Involved in the study	
☒	☐	ChIP-seq
☒	☐	Flow cytometry
☒	☐	MRI-based neuroimaging

Human research participants

Policy information about studies involving human research participants

Population characteristics	Demographic and clinical characteristics of these patients were provided in Supplementary Table S2 and S3.
Recruitment	A total of 58 subjects, including 35 laboratory-confirmed COVID-19 patients, 10 SARS-CoV-2 negative patients with various diseases (non-COVID-19) and 13 healthy adults were enrolled in this study. Specimens including throat swabs and anal swabs were collected from the patients during hospitalization (10-40) at Nantong Third Hospital Affiliated to Nantong University and from healthy adults when they visited the same hospital for physical examination. Sampling was performed using flexible, sterile, dry swabs, which can reach the posterior oropharynx and anus easily (approximately 2 inches) by the professionals at the hospital. At least two throat swabs at different days were available for 32 of 38 COVID-19 patients (Supplementary Fig S1).
Ethics oversight	The study was approved by Nantong Third Hospital Ethics Committee (EL2020006: 28 February 2020).

Note that full information on the approval of the study protocol must also be provided in the manuscript.
