# Supplementary material for: Temporal association between human upper respiratory and gut bacterial microbiomes during the course of COVID-19 in adults
Source: Commun Biol. 2021 Feb 18;4:240. doi: 10.1038/s42003-021-01796-w (PMC7893062; doi:10.1038/s42003-021-01796-w)
Supplement: Supplementary file 8 — Description of Additional Supplementary Files [file 42003_2021_1796_MOESM8_ESM.pdf]

Description of Additional Supplementary Files

File name: Supplementary Data 1.

Description: Throat and gut microbial abundances (phyla and genera). The 16S-rRNA gene sequences of all samples used in this study.

File name: Supplementary Data 2.

Description: Clinical index of COVID-19 patients in this study. Demographic and clinical characteristics of these patients.

File name: Supplementary Data 3.

Description: Dynamic changes of clinical parameters of 13 COVID-19 patients. The clinical index during the course of disease.

File name: Supplementary Data 4.

Description: The original raw source data needed to create figure files.

File name: Supplementary Code.

Description: The analyses scripts/code of the correlation analysis R package.
