# Supplementary material for: Temporal association between human upper respiratory and gut bacterial microbiomes during the course of COVID-19 in adults
Source: Commun Biol. 2021 Feb 18;4:240. doi: 10.1038/s42003-021-01796-w (PMC7893062; doi:10.1038/s42003-021-01796-w)
Supplement: Supplementary file 9 — Peer Review File [file 42003_2021_1796_MOESM9_ESM.pdf]

Reviewers' comments:

Reviewer #1 (Remarks to the Author):

The manuscript from Xu and colleagues describes temporal changes in microbiome composition during the course of SARS-CoV-2 infections. The study finds that the microbiome of COVID-19 patients can be classified into 3 or 4 categories (for throat and gut samples respectively), reflecting the transition from dysbiosis (Types III and IV) to a microbiota that is more similar to the one found in healthy individuals (Type I). A similar pattern was observed in the gut and in the respiratory tract.
Overall, the manuscript addresses an important knowledge gap and brings a coherent story. Although the results are reasonable, I think the small number of samples prevents appropriate statistical tests to support the main conclusions of the paper. I understand that clinical data can be difficult to collect, but at very least the manuscript needs to be upfront about these limitations and should take them into consideration when interpreting the results. I have several major concerns that would need to be addressed before I can recommend this manuscript for publication.

The main conclusions of this study are based on the diversity and composition of community types and their putative link with a dysbiosis-to-health transition. In particular, community types III and IV have a very low diversity and are dominated by a few seemly pathogenic species, which leads the authors to conclude that these samples are more severe cases of dysbiosis. However, those community types are also the ones with the lowest number of samples. In the respiratory tract dataset for example: community type IV has only 4 samples, while type II has 44, therefore it is logical that community type IV has a lower diversity. I suggest the diversity indices to be corrected for sample size before they are used as a dysbiosis marker.

The other argument was that community types III and IV are dominated by pathogenic bacteria, especially Pseudomonas and Rothia. The paper specifically argues that "Pseudomonas is a well-known pathogenic bacterium, and rarely found in healthy individuals." (Line 281). This is not correct, Pseudomonas can be pathogenic but it is also commonly found in healthy individuals. It is actually one of the most common bacteria in the saliva (Ruan, et al. 2020 "Healthy Human Gastrointestinal Microbiome: Composition and Function After a Decade of Exploration." Digestive Diseases and Sciences). Rhotia, the most abundant bacteria in community type IV, is also a common member of a healthy oral microbiome (Uranga et al. 2020 "Commensal Oral Rothia mucilaginosa Produces Enterobactin, a Metal-Chelating Siderophore." MSystems). Therefore, I cannot see any evidence that the community types I-IV reflect a transition from health to dysbiosis.

There is also no information about the actual health status of the patients to support the link with health and dysbiosis. We can assume that the patients were admitted to the hospital in poor health and progressively got better, but I would expect that the improvement is not linear (i.e. did any of the patients got worse during their stay at the hospital, before getting better?)

The number of samples from dysbiotic-like community types are very small. In the respiratory tract for example, community type IV was only observed in 3 patients, including the one with severe symptoms. Overall, these numbers preclude making meaningful statistical tests to confirm if community types are associated with COVID-19 transition stages. I suggest considering using a continuous variable (rather than categories) to associate community diversity with COVID-19 progression, which might give more statistical power to address the study's questions.

I found some inconsistencies in the description of the sampling that needs clarification:
The paper reads "A total of 63 subjects, including 35 laboratory-confirmed COVID-19 patients, 10 SARS-CoV-2 negative patients with various diseases (non-COVID-19) and 15 healthy adults were enrolled in this study". 35 + 15 + 10 = 60, so what are the 3 extra subjects?

More importantly - Supplementary figure 1 indicates that some of the COVID-19 patients (e.g. p05, p11) never tested positive for COVID-19, yet they seem to be considered COVID-19 patients in the analyses(Figure 1). Please explain. Patients 05 and 11 are the only ones where community type IV was observed in early stages of disease, so this obviously needs clarification. Supplementary methods are mentioned in the text (line 73) but were not provided.

Specific comments:

The paper states that all patients (except p09) had mild symptoms. Is it a normal procedure to hospitalize patients with mild symptoms?

L 82- 83: "The vast majority of the specimens of COVID-19 patients were divided into four community types, called I-IV, and 6 specimens were included in the NP type". I did not understand this analysis. Was this a cross-validation? How did you proceed with the 6 specimens that were classified as "non-COVID-19 patients"?

Paragraph starting in L 108: I think this is an overinterpretation of the result. For example: "Prominent microbiome community type shifts from early lower-diversity community types (NP, IV or II) towards later higher-diversity types (II or I) were observed in 9/24 COVID-19 adults who had specimens at two or more time points." In other words – these transitions were observed in only 37% of the data. The graphs show that the results are not that obvious, and community shifts are far from "prominent".

Section starting on L 128:
Were anal swabs also collected for healthy and non-covid-19 patients?

L 164 – 166: How were those bacterial genera selected?

L 197 – 200: I think this is an overstatement. It can be observed that some bacterial genera co-occur, but there too few samples and time points to make inferences about community succession. I suggest to also correct these networks for sample size. The authors could build networks of groups II and I with just 4 samples for example, to confirm that the increased complexity in these groups is not an artifact of sample size.

L 313: the manuscript states "Lastly, fecal microbiota transplantation may be considered as another treatment choice." This is a dangerous statement to make. Considering the current circumstances, I can imagine people may try to perform fecal transplants at home to treat COVID-19 after reading this. There is no data in this manuscript to support that fecal transplants can be used to treat COVID-19.

Reviewer #2 (Remarks to the Author):

In the present study, the authors used throat and anal samples from 35 COVID-19 adults and 15 controls to profile changes of the microbiome composition associated with COVID-19 infection by 16S rRNA gene sequencing.
The authors claim to show alterations of respiratory and intestinal communities associated with the viral infection, which may potential impact on the outcome. Moreover, they claim having uncovered a new airway-gut microbial axis.

The authors address interesting and important questions. However, I have serious concerns regarding lack of statistical power, choice of statistical methodology, lack of external validation and

often times highly speculative or erroneous conclusions from the present results. Specific comments are listed below.

I have serious doubts that throat swabs represent "the respiratory microbiota", wouldn't specimens collected form deeper respiratory tract localizations better represent the airway microbiota (although I acknowledge that these are more difficult to collect).

Wot 35 cases and only 15(!) controls the study is severely limited by the sample sizes and the representability of the given results is highly questionable.

The community typing using DMM modelling is highly problematic as the models were trained on a very small cohort – and the validity of the inferred community types needs to be validated in an (sufficiently large) independent cohort before further downstream results are inferred based on assumptions derived from this model.

The separation in the ordination plots between the "community types" is not surprising given that given that the very inferred or separated beforehand on the same cohort with a different method (DMM), this only shows that the methods comply. The clusters could show up simply by chance, the reliability of the clusters/community types has to proofed using an independent cohort.

Line 90, why were "the top 30 genera" chosen to represent the community types? This seems arbitrary to me, why not e.g. 50? Why isn't the number of genera rather chosen based on abundance or persistence, e.g. genera with at least 0.1% (or 1%) mean relative abundance and presence in at least 50% of the samples, this would be a more convincing definition of a kind of core microbiota.

The inference of any function or medical implication of identified bacteria here is highly problematic as the authors work with 16S data. E.g., line 96 ff. Pseudomans is not necessarily pathogenic – as by far not species or strains belonging to this genus exhibit pathogenic properties.

Line 164, the choice of "representative bacteria" is highly problematic, as indeed the functional relevance of throat bacteria in general, let alone in the context of COVID-19, is uncertain. Also, the term "probiotics" for Bifidobacterium and Faecalibacterium is inadequate, only certain strains of certain Bifidobacterium species are entailed in some commercially available probiotics, a (next-generation) probiotic potential of F. prausnitzii remains to be proven convincingly yet.

Generally, the throughout the text there is an uncritical use of the attribution of being beneficial or pathogenic, to date this ascription is possible for only very few bacteria based on genus-level taxonomic resolution alone.

The whole part on bacterial cooccurrence networks is highly dubious. As microbiome data generated by 16S sequencing is of compositional nature appropriate correlation methods have to used here, such as SparCC https://bitbucket.org/yonatanf/sparcc/src/default/

At least before spearman correlation the 16S data should be transformed according to the centered log ratio transformation.
https://www.frontiersin.org/articles/10.3389/fmicb.2017.02224/full?report=reader

Also, the conclusion that any statistically significant correlations indicate "bacterial translocation" (line 194) is highly speculative. This hypothesis needs to be addressed with more sophisticated methods.

From the given study design and based on the statistical methods applied, it cannot be concluded that microbiota is altered by COVID-19 and restored upon resolution of infection. First, because

criteria for alteration in this context are very difficult to define, and second because in order to show that a longitudinal study is required to show changes in microbial composition during and after infection.

Reviewer #3 (Remarks to the Author):

This is an interesting paper in an emerging field, and seeks to answer an important question concerning the gut-lung axis. The primary claims of the paper are that the throat and gut microbiota of COVID-19 infected adults can be characterised into different community types, beginning with a low diversity population soon after infection and a restoration back to diverse microbiota that in synchronous in the respiratory tract and the gut.
Overall, the researchers have done very well to draw conclusions from a limited dataset to provide timely information to assist with the current pandemic. Their attention to the precise details of the data and their longitudinal sampling is commendable. This work is very interesting and could provide valuable insight into the field and the long-term consequences of this novel disease, but additional considerations may help to improve the confidence of these conclusions:

Major Comments:
-Patient characteristics of healthy adults and non-COVID-19 patients should be included as supplementary information.
-Care should be taken describing the results of throat swabs. The authors describe the sampling as coming from the "posterior oropharynx" (Line 323) which is only one part of the upper respiratory tract. Referring to results more generally as "respiratory tract" may give the impression that results are associated with changes in the lower respiratory tract, particularly given references to the airways and lung throughout the discussion (e.g. lines 288-291)
-Line 83: It was noted that 6 COVID samples clustered with the NP type samples. Was there anything to distinguish these samples from other COVID samples?
-Authors discuss a return from low diversity to high diversity community types over time in throat samples, yet this was only observed in 9/24 patients (Line 112) while a reverse pattern was observed in four patients (Line 118-119). To my mind, this does not appear to demonstrate a clear trend that these patterns are consistent features of COVID-19 and additional sampling may be required to validate these findings. Alternatively, authors may be able to place additional emphasis on the association between the community types and time since appearance of symptoms in samples which were not a part of the longitudinal series but nevertheless support the hypothesis (e.g. p34, p18, p21, p28 in Fig 1e)
-Line 124-125: "These results indicate that the change of the respiratory microbiome might be closely associated with disease progression in COVID-19". The strength of this statement could be substantially improved by matching community types to some measure of clinical severity (e.g. symptom score, viral load, oxygen saturation, etc.) if this data is available.
-Line 161-163: Could the lack of correlation between community divergence and other parameters be due to the relatively small sample size available?
-Line 182-184: Authors discuss the hypothesis that co-occurrence networks between bacteria reflect crosstalk between the gut and lungs as a result of bacterial translocation, which is later described as a result of damage to the respiratory and gastrointestinal mucosa (Lines 194-195; Lines 261-262). Other processes may also explain these co-occurrences including (1) bacteria migrating from the oropharangeal site to the gut via swallowing and passage through the gastrointestinal tract (especially as several oral taxa are implicated) or (2) induction of immune responses at both sites which apply similar selective pressures to the microbiota. Could the authors please address these hypotheses.
-Similarly, is there any evidence available for a breakdown of mucosal barriers which may support the hypothesis of the authors (e.g. serum LPS or citrulline, etc.)? This may not be possible given the status of the patients but could provide valuable support for the hypothesis.

-Line 202-204: Increasedd Bifidobacterium and Faecalibacterium in throat samples were noted as evidence of restoration in microbiome composition, yet to the best of my knowledge these genera are not considered to be a normal part of the oropharangeal microbiome. Can the authors please provide some citations to demonstrate that these genera are representative of a healthy oropharangeal microbiome.
-Increases in Bifidobacterium and Faecalibacterium are noted as signs of improvement and restoration (e.g. Lines 164-170; 202-204) but the authors note several other commensal genera such as Bacteroides, Roseburia, Blautia, and Coprococcus, while other probiotic strains such as Lactobacillus are also prominent members of the microbiota. Can the authors explain why they chose to focus on Bifidobacterium and Faecalibacterium specifically, and not other probiotic genera? If other genera do not display similar patterns, does this perhaps indicate that the restoration of the microbiota is incomplete and may have long-term consequences?
-Line 228-230: The authors indicate that inconsistencies in changes observed during longitudinal sampling indicate that diversity characteristics of the throat microbiome were affected by COVID-19. Can the authors please explain why they determined this direction of causality (i.e. is it possible that diversity characteristics may have rather influenced the progression of COVID-19 and not vice versa).
-Line 268-272: It is stated that the gut microbiome appeared to have a faster restoration to increased bacterial diversity, but this phenomenon did not appear to be described in the results section. Indeed, Figure 3a seems to suggest that the progression to more diverse community types was occurring at the same time in the two body sites. Could the authors please provide clarity for the justification of this statement.
-Similarly, in Lines 270-272 the authors describe bacterial crosstalk which promotes restoration of the respiratory microbiota. How do the authors propose that bacteria are translocated from the gut to the oropharynx, particularly if mucosal integrity is beginning to recover concurrent with restoration of the microbiome?

Minor comments:
-Title should be revised to make it clear that this work specifically investigated bacterial communities, and not the broader microbiome (including viruses, archaea and fungi)
-Line 30: It may not be accurate to say that this paper "addresses the question" of whether microbiomes "affect disease progression". Although the authors show longitudinal changes, they do not demonstrate causality or a functional role and should be cautious of over-emphasising their results
-Line 36: replace "they had a" with "their
-descriptions of "bacterial interactions" (e.g. line 36, line 193, etc) may be interpreted to mean that the authors have evidence of bacterial cells interacting, whereas the analysis performed only demonstrated correlations and co-occurrence. Revised word should be considered to avoid any confusion
-Lines 49-55: Sentence is long and difficult to follow. Consider breaking it up into smaller sentences
-Line 61: delete "but"
-Please avoid using "significantly" where statistical analysis has not been applied (e.g. line 122 refers to a change in a single sample). Consider perhaps using "substantially" or a similar word to emphasis the magnitude of changes.
-Line 166: The authors state that that relative abundances of commensals "appeared to be" correlated. This term is ambiguous – if a correlation was statistically significant then the result shouls be stated more conclusively. If it was merely identified as an association or trend then the term "correlated" should be avoided as this is a specific type of statistical analysis.
-Line 210: reference to "near-normal microbiota" may be difficult to define, especially in the gut samples where no samples were available from healthy controls. Perhaps rephrasing to "more diverse" or "greater abundance of commensals" may be a more accurate description

-Line 217-219: authors describe that the respiratory microbiome is more easily affected by the infection, but also admit that this has not been examined yet. The first half of this sentence should be rephrased to demonstrate this is a hypothesis and not a know phenomenon
-please describe how "mild" and "severe" cases of COVID-19 were distinguished in patients
-Line 233: please change "baseline" to "early infection" or similar, to avoid any ambiguity about whether the samples were collected prior to infection.
-Line 265: it seems contradictory to state that the gut microbiome is both "more stable" and "more plastic: than the respiratory microbiota. Please clarify
-Line 306: please change "will be particularly useful" to "may be useful" as SCFA production is influence by factors other than microbiome composition (e.g. diet, cross-feeding, etc.)
-Line 303 and 310: Please capitalise Pseudomonas

Response to reviewers' comments

Reviewer #1 (Remarks to the Author):

The manuscript from Xu and colleagues describes temporal changes in microbiome composition during the course of SARS-CoV-2 infections. The study finds that the microbiome of COVID-19 patients can be classified into 3 or 4 categories (for throat and gut samples respectively), reflecting the transition from dysbiosis (Types III and IV) to a microbiota that is more similar to the one found in healthy individuals (Type I). A similar pattern was observed in the gut and in the respiratory tract.
Overall, the manuscript addresses an important knowledge gap and brings a coherent story. Although the results are reasonable, I think the small number of samples prevents appropriate statistical tests to support the main conclusions of the paper. I understand that clinical data can be difficult to collect, but at very least the manuscript needs to be upfront about these limitations and should take them into consideration when interpreting the results. I have several major concerns that would need to be addressed before I can recommend this manuscript for publication.
Authors: Thank you for your crucial comments and suggestions. We agree that the small number of patients is a major limitation of our paper, and appreciate your understanding in the difficulty of sample collection. We discussed this carefully in the Discussion section of the revised MS.

The main conclusions of this study are based on the diversity and composition of community types and their putative link with a dysbiosis-to-health transition. In particular, community types III and IV have a very low diversity and are dominated by a few seemly pathogenic species, which leads the authors to conclude that these samples are more severe cases of dysbiosis. However, those community types are also the ones with the lowest number of samples. In the respiratory tract dataset for example: community type IV has only 4 samples, while type II has 44, therefore it is logical that community type IV has a lower diversity. I suggest the diversity indices to be corrected for sample size before they are used as a dysbiosis marker.
Authors: As suggested, we employed the Margalef's index (Clifford HT, Stephenson W. 1975. An introduction to numerical classification. London: Academic Press; Perspectives in marine Biology: 323-349) for comparing the a-diversity difference among community types. The Margalef's index is created to compensate for the effects of sample size by dividing the number of species in a sample by the natural log of the number of organisms collected. The sample size-controlled diversities reflected by Margalef's index are similar to those observed in our previous analyses (Fig. R1), indicating that sample size has minor influence on the microbiome diversity. We added

the results of sample size-controlled diversities in both supplementary Fig. S3 and revised MS (Lines 89-90).

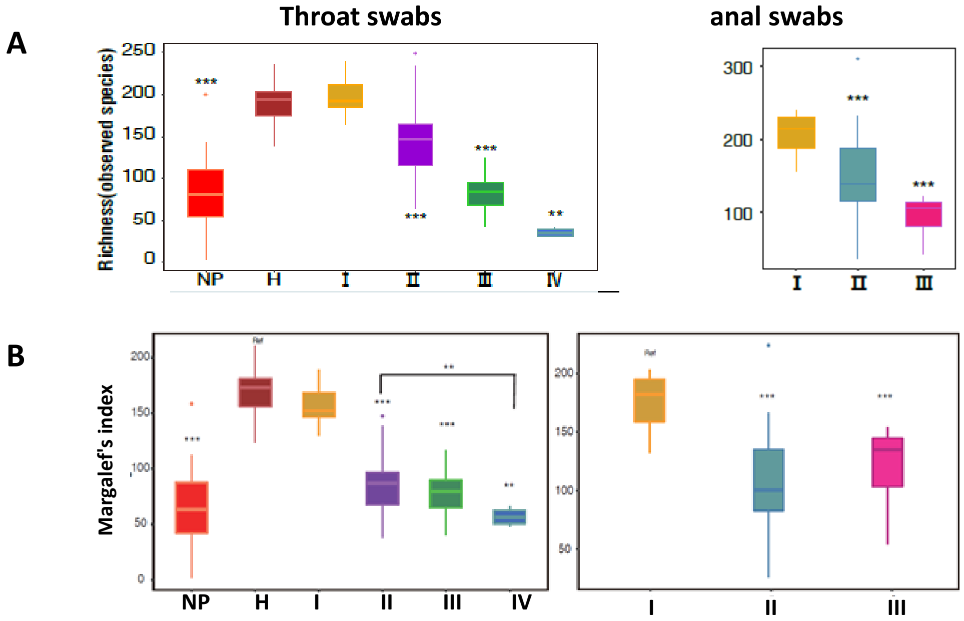

Fig. R1. Comparison of the microbiome diversity with (B) and without (A) sample size-controlled analyses.

The other argument was that community types III and IV are dominated by pathogenic bacteria, especially *Pseudomonas* and *Rothia*. The paper specifically argues that "*Pseudomonas* is a well-known pathogenic bacterium, and rarely found in healthy individuals." (Line 281). This is not correct, *Pseudomonas* can be pathogenic but it is also commonly found in healthy individuals. It is actually one of the most common bacteria in the saliva (Ruan, et al. 2020 "Healthy Human Gastrointestinal Microbiome: Composition and Function After a Decade of Exploration." Digestive Diseases and Sciences). *Rothia*, the most abundant bacteria in community type IV, is also a common member of a healthy oral microbiome (Uranga et al. 2020 "Commensal Oral *Rothia* mucilaginosa Produces Enterobactin, a Metal-Chelating Siderophore." MSystems). Therefore, I cannot see any evidence that the community types I-IV reflect a transition from health to dysbiosis.

Authors: We thank the reviewer for correctly indicated that *Pseudomonas* and *Rothia* are normally non-pathogenic and can be detected in healthy individuals. We carefully checked the raw data and performed a blasting analysis against rRNA_typestrains/16S_ribosomal_RNA database from NCBI. The result showed that the identified *Pseudomans* species had highest sequence similarity (100%) to the non-pathogenic species *Pseudomonas lactis* (Fig. R3 in page 12). *Pseudomonas lactis* was initially isolated from bovine raw milk (Fig. R4 in page 13).

With respect to *Rothia* spp., they are Gram-positive coccobacilli that cause a wide

range of serious infections, especially in immunocompromised hosts. Although some *Rothia* species (e.g. *R. dentocariosa, R. aeria, R. nasimurium*, and *R. amarae*) are part of the normal flora of the human oropharynx and upper respiratory tract, they are more commonly associated with various diseases (e.g. dental caries, periodontal disease, bacteremia, endocarditis, meningitis, peritonitis, bone and joint infections, pneumonia, skin and soft tissue infection, endophthalmitis, etc.) (*Rothia* bacteremia: a 10-year experience at Mayo Clinic, Rochester, Minnesota. J Clin Microbiol, 2014, 52(9):3184–3189.). In particular, invasive infections occur predominantly in immunocompromised hosts, but rarely in healthy hosts. In this study, the identified *Rothia* species had the highest > 99% sequence similarity to *Rothia mucilaginosa* that is often associated with various diseases (e.g. bacteremia) (Fig. R2 below) (Ramanan P, Barreto JN, Osmon DR, Tosh PK. Rothia bacteremia: a 10-year experience at Mayo Clinic, Rochester, Minnesota. J Clin Microbiol. 2014 Sep;52(9):3184-9.). These bacteria are less common in healthy individuals as observed in some previous studies (Lloyd-Price, J., Abu-Ali, G. & Huttenhower, C. The healthy human microbiome. Genome Med, 2016, Apr 27;8(1):51).

In this study, we found that healthy controls and most COVID-19 patients carried very low abundance of *Pseudomonas* and *Rothia,* and therefore a high abundance of *Pseudomonas lactis* (often in bovine raw milk) and *Rothia* in community type III and IV are consistent with a probable disruption of homeostasis, as the result of inflammation that is known to exist in most COVID-19 patients. In view of the concerns and the knowledge in literature, we modified our description on the significance of the variation of these bacterial species in the revised manuscript. .

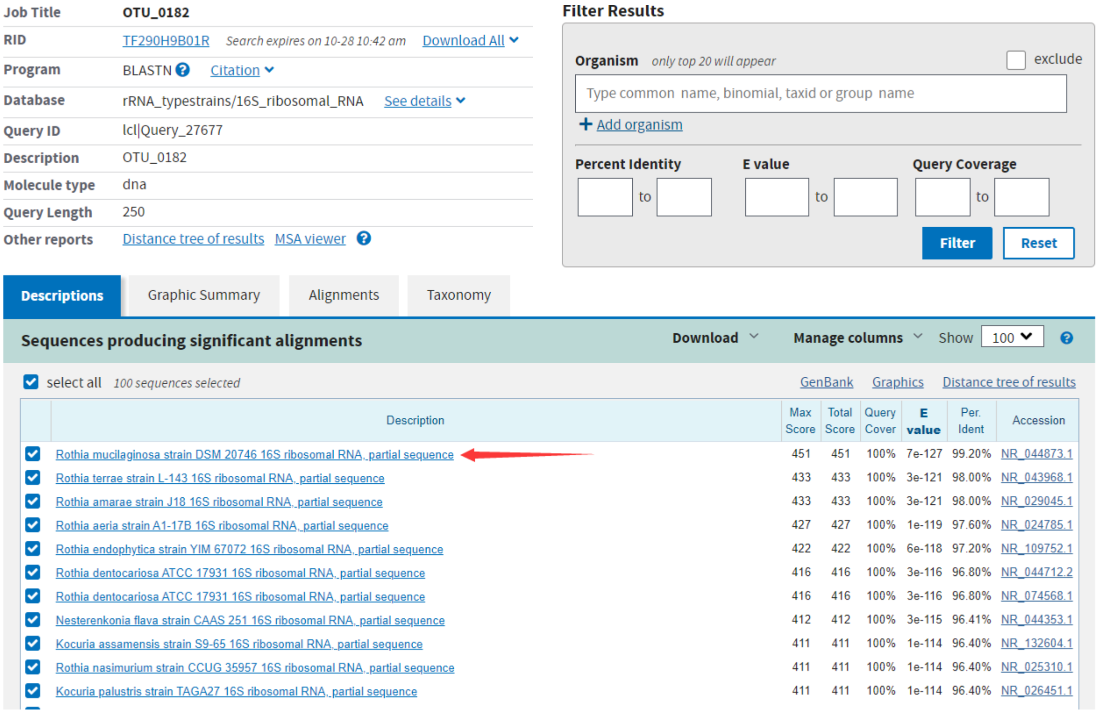

Description	Max Score	Total Score	Query Cover	E value	Per. Ident	Accession
Rothia mucilaginosa strain DSM 20746 16S ribosomal RNA, partial sequence	451	451	100%	7e-127	99.20%	NR_044873.1
Rothia terrae strain L-143 16S ribosomal RNA, partial sequence	433	433	100%	3e-121	98.00%	NR_043968.1
Rothia amarae strain J18 16S ribosomal RNA, partial sequence	433	433	100%	3e-121	98.00%	NR_029045.1
Rothia aeria strain A1-17B 16S ribosomal RNA, partial sequence	427	427	100%	1e-119	97.60%	NR_024785.1
Rothia endophytica strain YIM 67072 16S ribosomal RNA, partial sequence	422	422	100%	6e-118	97.20%	NR_109752.1
Rothia dentocariosa ATCC 17931 16S ribosomal RNA, partial sequence	416	416	100%	3e-116	96.80%	NR_044712.2
Rothia dentocariosa ATCC 17931 16S ribosomal RNA, partial sequence	416	416	100%	3e-116	96.80%	NR_074568.1
Nesterenkonia flava strain CAAS 251 16S ribosomal RNA, partial sequence	412	412	100%	3e-115	96.41%	NR_044353.1
Kocuria assamensis strain S9-65 16S ribosomal RNA, partial sequence	411	411	100%	1e-114	96.40%	NR_132604.1
Rothia nasimurium strain CCUG 35957 16S ribosomal RNA, partial sequence	411	411	100%	1e-114	96.40%	NR_025310.1
Kocuria palustris strain TAGA27 16S ribosomal RNA, partial sequence	411	411	100%	1e-114	96.40%	NR_026451.1

Fig. R2. The blasting results of *Rothia* species.

There is also no information about the actual health status of the patients to support the link with health and dysbiosis. We can assume that the patients were admitted to the hospital in poor health and progressively got better, but I would expect that the improvement is not linear (i.e. did any of the patients got worse during their stay at the hospital, before getting better?)

Authors: The reviewer is quite right to make these assumptions. To clarify the health status of patients, we added a supplementary table S4 to show the dynamic changes of clinical parameters of 13 COVID-19 patients, and added correlation analyses between the health status (clinical parameters) and the microbiome changes (please see new supplementary Fig. S10: below) in 8 patients.

Overall, almost all patients included in this study (except patient P09) had mild symptoms when they were hospitalized and during hospitalization. Patient P09 appeared to have deteriorated clinically during hospitalization.

The number of samples from dysbiotic-like community types are very small. In the respiratory tract for example, community type IV was only observed in 3 patients, including the one with severe symptoms. Overall, these numbers preclude making meaningful statistical tests to confirm if community types are associated with COVID-19 transition stages. I suggest considering using a continuous variable (rather than categories) to associate community diversity with COVID-19 progression, which might give more statistical power to address the study's questions.

Authors: We agree that the small sample (patient) numbers (especially those with community type IV) is a major limitation of this study, and it is hard to draw a strong association between community types and COVID-19 transition stages.

As suggested, we added the dynamic change of microbiome diversity over time in new supplementary Fig. S5-S6. The dynamics of microbiome compositions over time have been previously shown in supplementary Fig. S4, S7, and Fig. 4. The microbiome diversity did not show consistent trend among these patients (new supplementary Fig. S6), indicating individual variability of microbial community diversities associated with COVID-19. Therefore, we toned down our conclusion (association between community types and clinical recovery of COVID-19), and interpreted our results with caution.

Supplementary Fig. S5

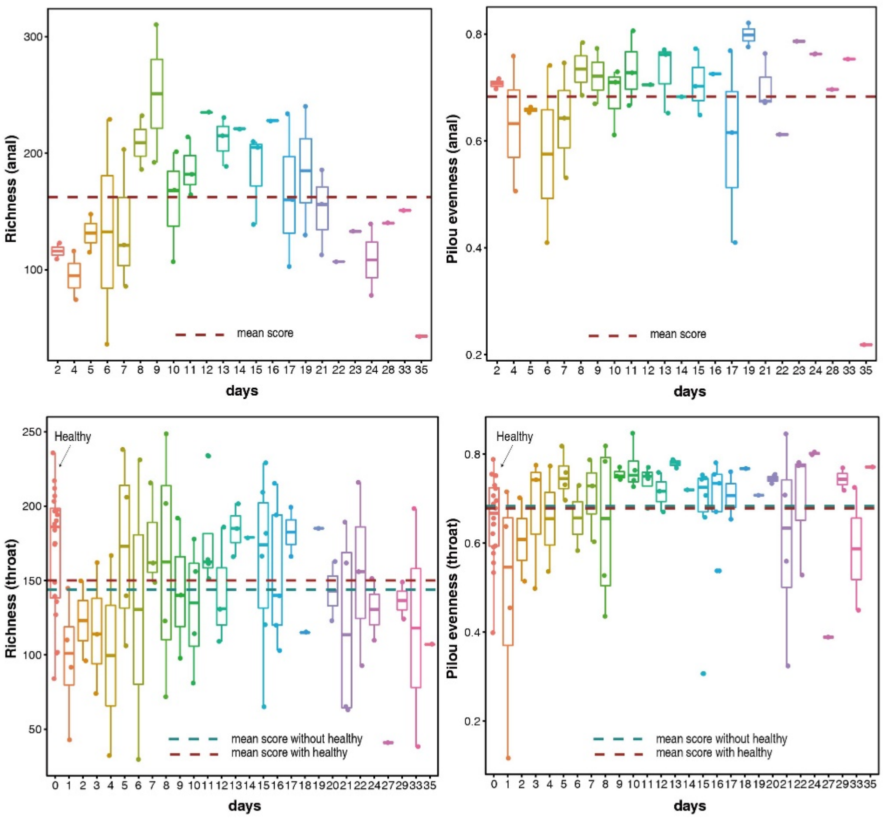

New supplementary Fig. S5. Dynamic changes of alpha-diversity since appearance of symptoms in COVID-19 patients.

A

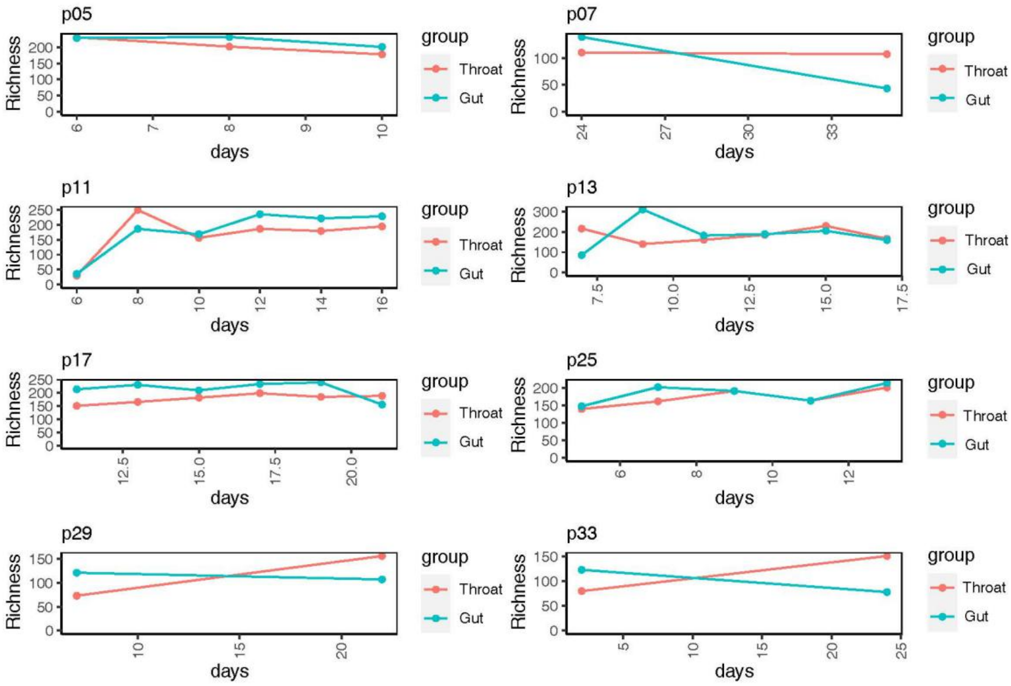

B

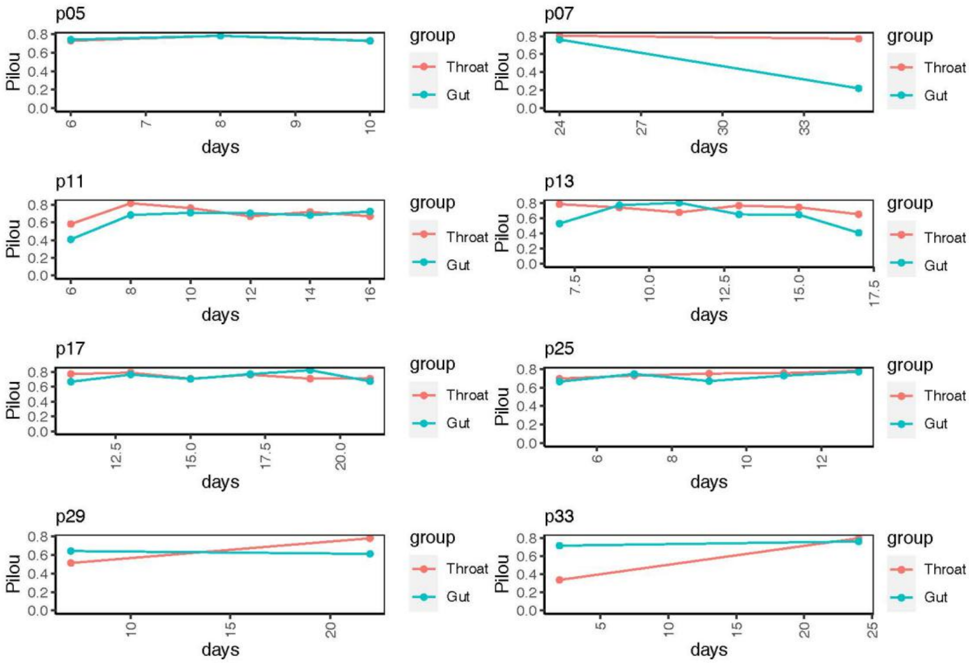

New supplementary Fig. S6. Dynamic changes of microbiome diversity during COVID-19 in eight patients. A: Richness, B: Peilou evenness.

I found some inconsistencies in the description of the sampling that needs clarification: The paper reads "A total of 63 subjects, including 35 laboratory-confirmed COVID-19 patients, 10 SARS-CoV-2 negative patients with various diseases (non-COVID-19) and 15 healthy adults were enrolled in this study". 35 + 15 + 10 = 60, so what are the 3 extra subjects?

Authors: We thank the reviewer for pointing out errors in counting. We have checked the numbers to make sure their consistency in the revised manuscript.

More importantly - Supplementary figure 1 indicates that some of the COVID-19 patients (e.g. p05, p11) never tested positive for COVID-19, yet they seem to be considered COVID-19 patients in the analyses (Figure 1). Please explain. Patients 05 and 11 are the only ones where community type IV was observed in early stages of disease, so this obviously needs clarification.

Authors: First, all recruited patients were confirmed to have COVID-19 infection by the local CDC. The criterion for diagnosis of COVID-19 infection was positive for two or more different SARS-CoV-2 genes by RT-qPCR assay.

During the pandemic, China national and local CDC are responsible for prevention, control and management of COVID-19 infection. Some of the recruited patients (e.g. P05, P11, P13, P25, P27 etc.) were initially found/confirmed by local CDC in Nantong city, and then admitted, as required, to Nantong Third Hospital Affiliated to Nantong University for treatment and quarantine. At the time of sampling, some patients have

recovered and become test-negative. Alternatively, the particular samples tested might have been false-negative.

We added more information on these patients in supplementary Fig. S1. In addition, we mentioned that community type IV was observed in early stages of disease in only two patients P05 and P11 although SARS-CoV-2 RNA has become negative in their throat swabs.

Supplementary methods are mentioned in the text (line 73) but were not provided.

Authors: More detailed methodology is provided in the "Methods" section of the main text, rather than in a Supplementary file. We deleted "See Supplementary Methods" from this sentence.

Specific comments:

The paper states that all patients (except p09) had mild symptoms. Is it a normal procedure to hospitalize patients with mild symptoms?

Authors: Yes, in China, all confirmed patients (including asymptomatic, mild or severe cases) are required to be admitted to designated hospitals for treatment and/or quarantine.

L 82- 83: "The vast majority of the specimens of COVID-19 patients were divided into four community types, called I-IV, and 6 specimens were included in the NP type". I did not understand this analysis. Was this a cross-validation? How did you proceed with the 6 specimens that were classified as "non-COVID-19 patients"?

Authors: In our study, we included 10 SARS-CoV-2 RNA negative patients who were hospitalized because of other diseases, and designated them as non-COVID-19 patients (NP). In the DMM cluster analysis, samples of the NP group clustered together to form an independent cluster, and showed a community feature distinct from the groups of healthy control or COVID-19 patients.

To characterize each cluster, group distribution was analyzed and compared (new supplementary Fig. S2). The H cluster that only contains healthy controls represents normal throat microbiome. In the NP cluster, there was a significantly higher proportion of NP patients (55.6%) than COVID-19 patients (33.3%) and healthy controls (11.1%) ($P<0.01$). Thereby, the NP cluster is more likely representative of a diverse microbiome status associated with various other diseases (new supplementary Fig. S2). Whereas the COVID-19 patients are more homogeneous at a given disease stage, and their microbiome was mainly predominated by community type II regardless of in throat swabs (56.6%) and in anal swabs (53.3%).

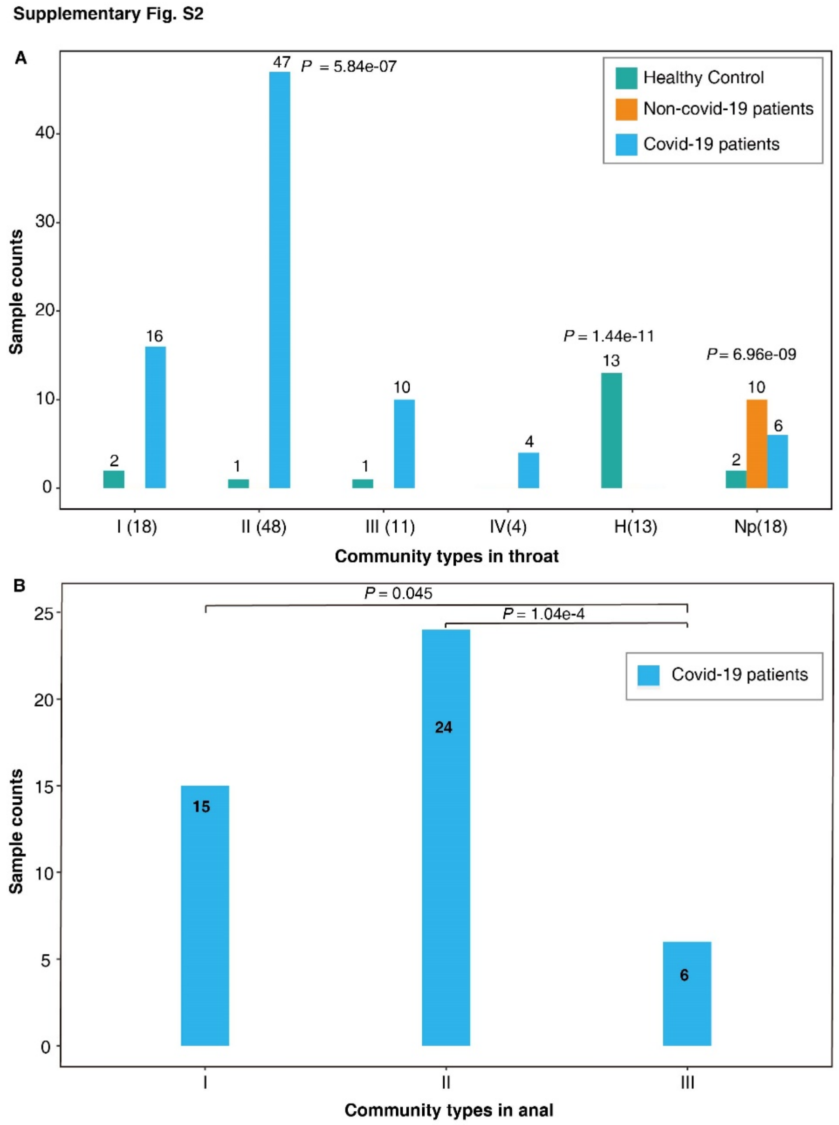

New supplementary Fig. S2. Group distribution characteristics of each community cluster.

Paragraph starting in L 108: I think this is an over-interpretation of the result. For example: "Prominent microbiome community type shifts from early lower-diversity community types (NP, IV or II) towards later higher-diversity types (II or I) were observed in 9/24 COVID-19 adults who had specimens at two or more time points." In other words – these transitions were observed in only 37% of the data. The graphs show that the results are not that obvious, and community shifts are far from "prominent".

Authors: We appreciate this critical crucial comment, and toned down our conclusion accordingly in the revised manuscript.

Section starting on L 128:

Were anal swabs also collected for healthy and non-covid-19 patients?

Authors: The anal swabs were not collected from healthy and non-COVID-19 patients.

L 164 – 166: How were those bacterial genera selected?

Authors: The top indicator bacteria with at least 0.5 indicator values were selected from each cluster (Fig. 1d and 2d), and subjected to analyses with several core functional bacteria (e.g. *Faecalibacterium, Lactobacillus* and *Bifidobacterium*) in gut.

L 197 – 200: I think this is an overstatement. It can be observed that some bacterial genera co-occur, but there too few samples and time points to make inferences about community succession. I suggest to also correct these networks for sample size. The authors could build networks of groups II and I with just 4 samples for example, to confirm that the increased complexity in these groups is not an artifact of sample size.

Authors: This is a reasonable comment. According to the suggestion, we reanalyzed the 16S datasets from all 74 samples of 13 COVID-19 patients (corresponding to old Supplementary Fig. S3 or new Supplementary Fig. S4) by log transform the raw data before performing spearman correlation test. A total of 153 co-occurred pairs with $|r| > 0.7$ under FDR-adjusted $P < 0.05$ were identified and visualized by Cytoscape version 3.8.0. We presented the new co-occurrence networks in the revised manuscript. In the new co-occurrence networks, we found that 1) there were cross-talks of microbial compositions between and within host niches; 2) there was a competitive relationship between Gut-type-II and Gut-type-I mediated by significantly negative correlation between gut bacterial genera *Neisseria* and *Bacteroides*; 3) *Bacter*oides may modulate the cross-talk between Throat-type H and Gut-type-I (shown in page 15).

L 313: the manuscript states "Lastly, fecal microbiota transplantation may be considered as another treatment choice." This is a dangerous statement to make. Considering the current circumstances, I can imagine people may try to perform fecal transplants at home to treat COVID-19 after reading this. There is no data in this manuscript to support that fecal transplants can be used to treat COVID-19.

Authors: We appreciate the reviewer's critical comment and therefore deleted this sentence from the revised manuscript.

Reviewer #2 (Remarks to the Author):

In the present study, the authors used throat and anal samples from 35 COVID-19 adults and 15 controls to profile changes of the microbiome composition associated with COVID-19 infection by 16S rRNA gene sequencing.

The authors claim to show alterations of respiratory and intestinal communities associated with the viral infection, which may potential impact on the outcome.

Moreover, they claim having uncovered a new airway-gut microbial axis.

The authors address interesting and important questions. However, I have serious concerns regarding lack of statistical power, choice of statistical methodology, lack of external validation and often times highly speculative or erroneous conclusions from the present results. Specific comments are listed below.

Authors: We thank the reviewer for these critiques. We have carefully revised our manuscript according to all comments and suggestions as detailed below.

I have serious doubts that throat swabs represent "the respiratory microbiota", wouldn't specimens collected form deeper respiratory tract localizations better represent the airway microbiota (although I acknowledge that these are more difficult to collect).

Authors: We revised the text to be more precise. Specifically, "respiratory microbiota" was more clearly written as "upper respiratory microbiota" in the revised manuscript.

Wot 35 cases and only 15(!) controls the study is severely limited by the sample sizes and the representability of the given results is highly questionable.

Authors: We agree that sample size is a limitation of this study. Despite the small sample size, we discovered 13 of 19 healthy controls (throat swabs) can yield microbiomes clustered clearly different from that of other groups in the DMM modelling analysis (Fig. 1a and 1b). However, we discussed this issue and cautioned the readers that our results may not be representative of all patient groups.

The community typing using DMM modelling is highly problematic as the models were trained on a very small cohort – and the validity of the inferred community types needs to be validated in an (sufficiently large) independent cohort before further downstream results are inferred based on assumptions derived from this model.

Authors: Even with small sample size, this type of analyses have yielded interesting information previously. In the original paper that describes DMM modelling method (Holmes I, Harris K, Quince C. 2012. Dirichlet Multinomial Mixtures: Generative Models for Microbial Metagenomics. PLoS ONE 7: e30126.), the authors used two datasets to test the DMM availability. Based on the small dataset (78 samples) of inflammatory bowel disease (IBD) phenotypes, the authors found ileal Crohn's disease (ICD) was associated with a more variable community. In another study (Vandeputte D, et al. 2016. Stool consistency is strongly associated with gut microbiota richness and composition, enterotypes and bacterial growth rates. Gut 65: 57-62.), the authors used 16S DNA datasets from faecal samples of 53 healthy women for performing DMM modeling analysis and found strong associations between stool consistency and enterotype distribution. In our study, 112 throat swabs and 45 anal swabs were used to investigate the upper respiratory and gut microbial community type distribution in

COVID-19 patients, respectively. We believed that the obtained results are reliable. Of course, we would like in the future to validate our current results with an increased sample size.

The separation in the ordination plots between the "community types" is not surprising given that the very inferred or separated beforehand on the same cohort with a different method (DMM), this only shows that the methods comply. The clusters could show up simply by chance, the reliability of the clusters/community types has to proofed using an independent cohort.

Authors: We thank the reviewer for concurring the methodology is sound. In DMM modeling (Holmes I, Harris K, Quince C. 2012. Dirichlet Multinomial Mixtures: Generative Models for Microbial Metagenomics. PLoS ONE 7: e30126; Ding T, Schloss PD. 2014. Dynamics and associations of microbial community types across the human body. Nature 509: 357-360.), the reliability of clusters is defined by both the minimum Laplace approximation value and the maximum posterior probability (at least 0.90).

In fact, all our samples were classified as different clusters by using the minimum Laplace approximation value and posterior probabilities equal to 1. Of course, it would be better if another independent cohort is used to test the generalizability and reliability of our findings in future studies.

Line 90, why were "the top 30 genera" chosen to represent the community types? This seems arbitrary to me, why not e.g. 50? Why isn't the number of genera rather chosen based on abundance or persistence, e.g. genera with at least 0.1% (or 1%) mean relative abundance and presence in at least 50% of the samples, this would be a more convincing definition of a kind of core microbiota.

Authors: We selected the top genera number based on what is needed to form a separate community. We agree the selection is a bit arbitrary. To address, we also performed analyses with varying genera numbers.

In throat microbiota, the top 30 genera contributed to 66% of cumulative differences that are similar to total variations of all bacterial genera applied to DMM modeling. In anal microbiota, the top 30 genera reached up to 68% of cumulative differences. Those descriptions can be seen from "**Indicator analysis in throat and gut community types**" section in Methods. We also added related descriptions in the text of revised MS (Lines 100-114).

We also compared the difference between top30 (contributed to 66% of cumulative differences) and top40 genera (contributed to 72% of cumulative differences) for the identification of indicators of each throat microbial cluster. We found that the results were similar (Fig. R3) because five new identified indicators from the top40 genera had

the average or median abundances higher than 1% only in 3-13% of samples but not in at least 50% of samples. So, those top30 genera representing at least 66% of cumulative differences is reliable to identify the indicator of throat & gut microbial clusters.

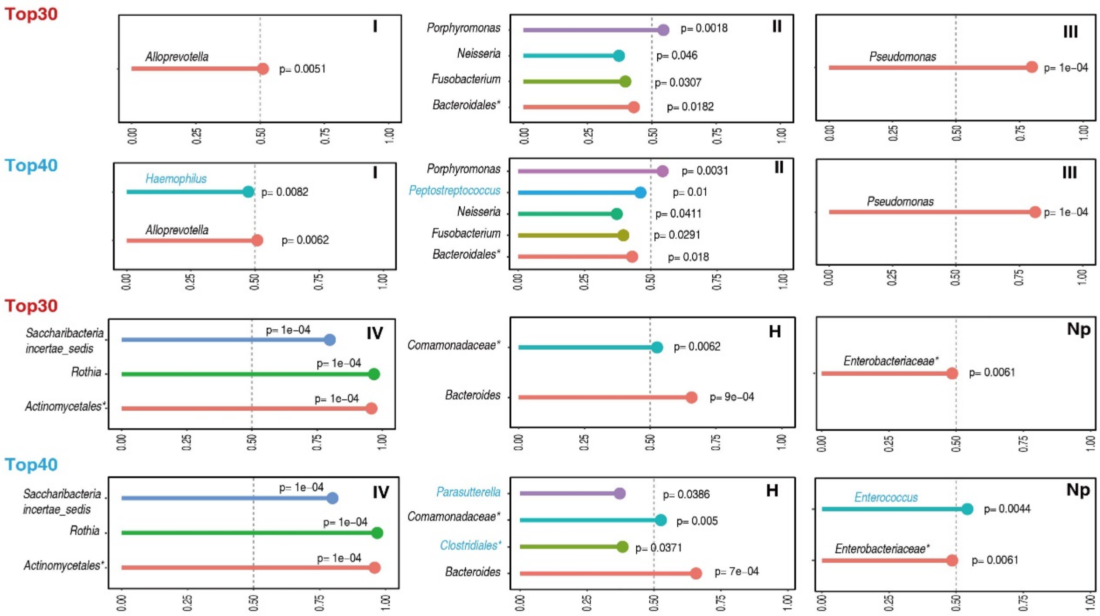

Fig. R3. Comparison of the indicator bacteria identified from top 30 and top 40 genera.

The inference of any function or medical implication of identified bacteria here is highly problematic as the authors work with 16S data. E.g., line 96 ff. Pseudomans is not necessarily pathogenic – as by far not species or strains belonging to this genus exhibit pathogenic properties.

Authors: We agree with the limitation when only 16S data were used. After checking the raw data by blasting analysis against rRNA_typestrains/16S_ribosomal_RNA database from NCBI, we found that the identified *Pseudomans* species had highest sequence similarity (100%) to the non-pathogenic species *Pseudomonas lactis* (Fig. R4). *Pseudomonas lactis* was initially isolated from bovine raw milk (von Neubeck M, et al. Pseudomonas lactis sp. nov. and Pseudomonas paralactis sp. nov., isolated from bovine raw milk. *Int J Syst Evol Microbiol.* 2017 Jun; 67(6):1656-1664). Accordingly, we revised the related results and conclusions.

Sequences producing significant alignments   Download   Manage columns   Show 100

select all *100 sequences selected*   GenBank   Graphics   Distance tree of results

Description	Max Score	Total Score	Query Cover	E value	Per. Ident	Accession
Pseudomonas lactis strain DSM 29167 16S ribosomal RNA, partial sequence	462	462	100%	3e-130	100.00%	NR_156986.1
Pseudomonas paralactis strain DSM 29164 16S ribosomal RNA, partial sequence	462	462	100%	3e-130	100.00%	NR_156987.1
Pseudomonas canadensis strain 2-92 16S ribosomal RNA, partial sequence	462	462	100%	3e-130	100.00%	NR_156852.1
Pseudomonas tolaasii strain ATCC 33618 16S ribosomal RNA, partial sequence	462	462	100%	3e-130	100.00%	NR_114481.1
Pseudomonas tolaasii strain NBRC 103163 16S ribosomal RNA, partial sequence	462	462	100%	3e-130	100.00%	NR_114227.1
Pseudomonas fluorescens strain NBRC 14160 16S ribosomal RNA, partial sequence	462	462	100%	3e-130	100.00%	NR_113647.1
Pseudomonas azotoformans strain NBRC 12693 16S ribosomal RNA, partial sequence	462	462	100%	3e-130	100.00%	NR_113600.1
Pseudomonas lurida strain P 513/18 16S ribosomal RNA, partial sequence	462	462	100%	3e-130	100.00%	NR_042199.1
Pseudomonas tolaasii 16S ribosomal RNA, partial sequence	462	462	100%	3e-130	100.00%	NR_117823.1
Pseudomonas trivialis strain P 513/19 16S ribosomal RNA, partial sequence	462	462	100%	3e-130	100.00%	NR_028987.1
Pseudomonas poae strain P 527/13 16S ribosomal RNA, partial sequence	462	462	100%	3e-130	100.00%	NR_028986.1
Pseudomonas fluorescens strain CCM 2115 16S ribosomal RNA, partial sequence	462	462	100%	3e-130	100.00%	NR_115715.1
Pseudomonas simiae strain OLi 16S ribosomal RNA, partial sequence	462	462	100%	3e-130	100.00%	NR_042392.1
Pseudomonas tolaasii NCPPB 2192 16S ribosomal RNA, partial sequence	462	462	100%	3e-130	100.00%	NR_114595.1
Pseudomonas meridiana strain CMS 38 16S ribosomal RNA, partial sequence	462	462	100%	3e-130	100.00%	NR_025587.1
Pseudomonas antarctica strain CMS 35 16S ribosomal RNA, partial sequence	462	462	100%	3e-130	100.00%	NR_025586.1
Pseudomonas fluorescens strain ATCC 13525 16S ribosomal RNA, partial sequence	462	462	100%	3e-130	100.00%	NR_114476.1
Pseudomonas costantinii strain CFBP 5705 16S ribosomal RNA, partial sequence	462	462	100%	3e-130	100.00%	NR_025164.1
Pseudomonas extremorientalis strain KMM 3447 16S ribosomal RNA, partial sequence	462	462	100%	3e-130	100.00%	NR_025174.1
Pseudomonas tolaasii strain LMG 2342 16S ribosomal RNA, partial sequence	462	462	100%	3e-130	100.00%	NR_041799.1

Fig. R4. The blasting results of *Pseudomans* species.

Line 164, the choice of "representative bacteria" is highly problematic, as indeed the functional relevance of throat bacteria in general, let alone in the context of COVID-19, is uncertain. Also, the term "probiotics" for Bifidobacterium and Faecalibacterium is inadequate, only certain strains of certain Bifidobacterium species are entailed in some commercially available probiotics, a (next-generation) probiotic potential of F. prausnitzii remains to be proven convincingly yet.

Authors: We agree with the reviewer. First, in the revised MS, we selected the top indicator bacteria with > 0.5 indicator values from each cluster (Fig. 1d and 2d) and several major core functional bacteria (e.g. *Faecalibacterium, Lactobacillus* and *Bifidobacterium*) in gut as the representative bacteria, and re-performed the analysis.

Second, the majority of the species of *Bifidobacterium* and *Faecalibacterium* are beneficial to host health. Anti-Inflammatory roles of gut bacteria *Faecalibacterium prausnitzii* have been revealed and confirmed by a series of studies (Sokol et al. 2008; Heinken et al. 2014; Miquel et al. 2015; Lopez-Siles et al. 2017; Martín et al. 2017). At least, these studies suggested benefical roles of gut bacteria *Faecalibacterium prausnitzii* to host health, although some of these bacteria may not be called as probiotics. Accordingly, we revised the term "probiotics" as "potential beneficial bacteria".

References:

a) Heinken A, Khan MT, Paglia G, Rodionov DA, Harmsen HJM, Thiele I. 2014. A functional metabolic map of *Faecalibacterium prausnitzii*, a beneficial human gut microbe. J Bacteriol 196(18):3289-302. doi:10.1128/jb.01780-14.

b) Lopez-Siles M, Duncan SH, Garcia-Gil LJ, Martinez-Medina M. 2017. *Faecalibacterium prausnitzii*: from microbiology to diagnostics and prognostics. The ISME Journal 11: 841-852.

c) Martín R, Miquel S, Benevides L, Bridonneau C, Robert V, Hudault S, Chain F, Berteau O,

Azevedo V, Chatel JM et al. 2017. Functional Characterization of Novel *Faecalibacterium prausnitzii* Strains Isolated from Healthy Volunteers: A Step Forward in the Use of F. prausnitzii as a Next-Generation Probiotic. Front Microbiol 8:1226

d) Miquel S, Leclerc M, Martin R, Chain F, Lenoir M, Raguideau S, Hudault S, Bridonneau C, Northen T, Bowen B et al. 2015. Identification of Metabolic Signatures Linked to Anti-Inflammatory Effects of *Faecalibacterium prausnitzii*. mBio 6(2):e00300-15.

e) Sokol H, Pigneur B, Watterlot L, Lakhdari O, Bermúdez-Humarán LG, Gratadoux J-J, Blugeon S, Bridonneau C, Furet J-P, Corthier G et al. 2008. *Faecalibacterium prausnitzii* is an anti-inflammatory commensal bacterium identified by gut microbiota analysis of Crohn disease patients. Proc Natl Acad Sci USA 105: 16731-16736.

Generally, the throughout the text there is an uncritical use of the attribution of being beneficial or pathogenic, to date this ascription is possible for only very few bacteria based on genus-level taxonomic resolution alone.

Authors: We agree with the reviewer. “Pathogenic” may be a relative concept. Some bacteria in healthy individuals might be pathogenic in immunocompromised individuals or under inflammatory status. For caution, we used relatively more neutral terms (e.g. potential pathogenic bacteria) in the revised MS.

The whole part on bacterial cooccurrence networks is highly dubious. As microbiome data generated by 16S sequencing is of compositional nature, appropriate correlation methods have to used here, such as SparCC https://bitbucket.org/yonatanf/sparcc/src/default/

Authors: As suggested, we re-performed the co-occurrence network analysis using the 16S data normalized by the centered log ratio transformation as described below.

At least before spearman correlation the 16S data should be transformed according to the centered log ratio transformation. https://www.frontiersin.org/articles/10.3389/fmicb.2017.02224/full?report=reader

Authors: As suggested, we transferred the 16S datasets from all 74 samples of 13 COVID-19 patients according to the centered log ratio transformation before performing spearman correlation. A total of 153 co-occurred pairs with $|r| > 0.7$ under FDR-adjusted $P < 0.05$ were identified and visualized by Cytoscape version 3.8.0. In new co-occurrence network, we found 1) obvious cross-talks of microbial compositions between and with niches; 2) a competitive relationship between Gut-type-B and Gut-type-A mediated by significantly negative interaction between gut bacterial genera *Neisseria* and *Bacteroides*, which might determine the microbiome shift from Gut-type-B to Gut-type-A during the progress of COVID-19; 3) *Bacteroides* may modulate the cross-talk between Throat-type H and Gut-type-A, which might be beneficial for the restoration of throat and gut microbiota associated with COVID-19. The new results

have been updated in the revised MS.

Fig. 5

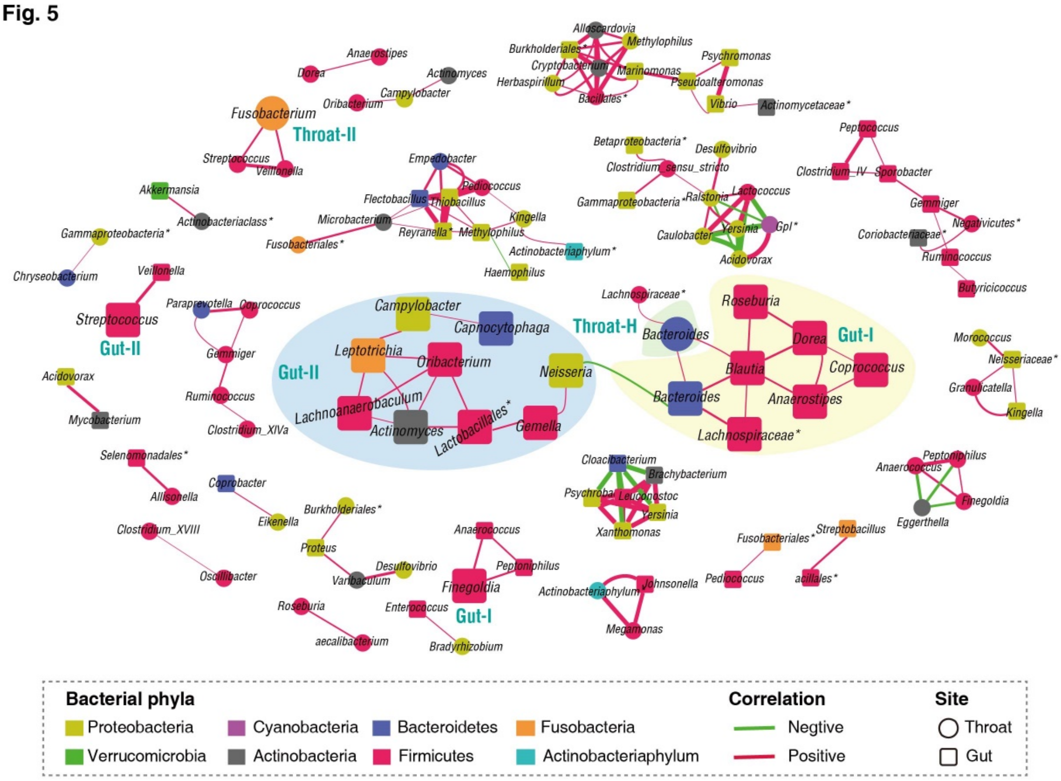

New Fig. 5 in the main text. Co-occurrence networks of gut and throat microbiota within 13 COVID-19 patients.

Also, the conclusion that any statistically significant correlations indicate "bacterial translocation" (line 194) is highly speculative. This hypothesis needs to be addressed with more sophisticated methods.

Authors: We added some new results (e.g. serum LPS levels) and explanations in the revised MS, and toned down some conclusions including those on bacterial translocation.

From the given study design and based on the statistical methods applied, it cannot be concluded that microbiota is altered by COVID-19 and restored upon resolution of infection. First, because criteria for alteration in this context are very difficult to define, and second because in order to show that a longitudinal study is required to show changes in microbial composition during and after infection.

Authors: Alteration in gut microbiota of COVID-19 patients have been previously reported (Zuo et al. 2020; Gu et al. 2020). In this study, by comparing with the healthy controls, we clearly found that the upper respiratory tract microbiotas of COVID-19 patients are altered in multiple levels of α-diversity (Fig. 1c), β-diversity (Fig. 1b), bacterial community types (Fig. 1a), and indicator bacteria (Fig. 1d), which are

consistent with and support previous observations. In particular, we observed the dynamic changes of the microbiota profiles at both upper respiratory tract and gut during acute infection and recovery phase of COVID-19. It is clear that the bacterial community types (along with the α-diversity and indicator bacteria) have changed over time since COVID-19 infection. In particular, the most prevalent microbial community type associated with COVID-19 patients is Throat-II, significantly distinct from that in healthy controls. Similarly, Gut-II significantly associated with COVID-19 patients is significantly different from known healthy gut microbial community structures (Arumugam et al. 2011; The Human Microbiome Project Consortium, 2012; Zhang et al. 2014).

References:

f) Zuo T, Zhang F, Lui GCY, Yeoh YK, Li AYL, Zhan H, Wan Y, Chung ACK, Cheung CP, Chen N, Lai CKC, Chen Z, Tso EYK, Fung KSC, Chan V, Ling L, Joynt G, Hui DSC, Chan FKL, Chan PKS, Ng SC. Alterations in Gut Microbiota of Patients With COVID-19 During Time of Hospitalization. Gastroenterology. 2020 Sep;159(3):944-955.e8.;

g) Gu S, Chen Y, Wu Z, Chen Y, Gao H, Lv L, Guo F, Zhang X, Luo R, Huang C, Lu H, Zheng B, Zhang J, Yan R, Zhang H, Jiang H, Xu Q, Guo J, Gong Y, Tang L, Li L. Alterations of the Gut Microbiota in Patients with COVID-19 or H1N1 Influenza. Clin Infect Dis. 2020 Jun 4:ciaa709.

h) The Human Microbiome Project Consortium. 2012. Structure, function and diversity of the healthy human microbiome. Nature 486: 207-214.

i) Arumugam M, Raes J, Pelletier E, Le Paslier D, Yamada T, Mende DR, Fernandes GR, Tap J, Bruls T,

j) Batto J-M et al. 2011. Enterotypes of the human gut microbiome. Nature 473: 174-180.

k) Zhang Z, Geng J, Tang X, Fan H, Xu J, Wen X, Ma Z, Shi P. 2014. Spatial heterogeneity and co-occurrence patterns of human mucosal-associated intestinal microbiota. ISME J 8: 881-893.

Reviewer #3 (Remarks to the Author):

This is an interesting paper in an emerging field, and seeks to answer an important question concerning the gut-lung axis. The primary claims of the paper are that the throat and gut microbiota of COVID-19 infected adults can be characterised into different community types, beginning with a low diversity population soon after infection and a restoration back to diverse microbiota that in synchronous in the respiratory tract and the gut.

Overall, the researchers have done very well to draw conclusions from a limited dataset to provide timely information to assist with the current pandemic. Their attention to the precise details of the data and their longitudinal sampling is commendable. This work is very interesting and could provide valuable insight into the field and the long-term consequences of this novel disease, but additional considerations may help to improve

the confidence of these conclusions:

Authors: We thank the reviewer for these positive comments.

Major Comments:

-Patient characteristics of healthy adults and non-COVID-19 patients should be included as supplementary information.

Authors: We provided the clinical information of the COVID-19 patients whenever possible, with the caveat that some demographic and clinical characteristics of healthy adults and non-COVID-19 patients are not available due to the emergency nature of the pandemic, and lock-down measure in some places.

-Care should be taken describing the results of throat swabs. The authors describe the sampling as coming from the "posterior oropharynx" (Line 323) which is only one part of the upper respiratory tract. Referring to results more generally as "respiratory tract" may give the impression that results are associated with changes in the lower respiratory tract, particularly given references to the airways and lung throughout the discussion (e.g. lines 288-291)

Authors: We thank the reviewer for pointing out this point. We revised "respiratory tract" and "airway" into "upper respiratory tract" in the revised MS.

-Line 83: It was noted that 6 COVID samples clustered with the NP type samples. Was there anything to distinguish these samples from other COVID samples?

Authors: In our study, 10 SARS-CoV-2 RNA negative patients who were hospitalized because of other diseases were included, and defined as non-COVID-19 patients (NP) group. In the DMM cluster analysis, all samples of the NP group clustered with those of 6 COVID-19 patients and two healthy controls, and formed a cluster, distinct from community features of majority of COVID-19 patients as well as healthy control. Because a significantly higher proportion (55.6%) of NP patients were included in this cluster than COVID-19 (33.3%) and healthy controls (11.1%) ($P<0.01$), this cluster represents more likely a diverse microbiome status associated with various other diseases (please see new supplementary Fig. S2 in page 8).

The 6 COVID-19 samples in NP cluster exhibited different bacterial characteristics from other COVID-19 samples, and can be distinguished from other COVID-19 samples by corresponding indictor bacteria (e.g. *Enterobacteriaceae* in NP type; other bacteria such as *Neisseria, Pseudomonas* etc. in community types I-IV) (Fig. 1d). The inclusion of 6 COVID-19 samples in the NP cluster might indicate that they shared similar microbiota profile to the NP group, and COVID-19 has diverse effects on microbiome.

-Authors discuss a return from low diversity to high diversity community types over time in throat samples, yet this was only observed in 9/24 patients (Line 112) while a reverse pattern was observed in four patients (Line 118-119). To my mind, this does not appear to demonstrate a clear trend that these patterns are consistent features of COVID-19 and additional sampling may be required to validate these findings. Alternatively, authors may be able to place additional emphasis on the association between the community types and time since appearance of symptoms in samples which were not a part of the longitudinal series but nevertheless support the hypothesis (e.g. p34, p18, p21, p28 in Fig 1e)

Authors: We thank the reviewer's comment and suggestion. First, we toned down our conclusion on the association between a microbiota change and COVID-19 disease stage. Second, we performed the correlation relationship analysis between the community types and sampling time. There was no significantly association to be identified. However, when we analyzed the community type characteristics of all samples, we found that the majority of COVID-19 samples belonged to microbiome community type II regardless of in upper respiratory tracts or the gut (New supplementary Fig. S2, see page 8). In other words, COVID-19-associated microbiome was characterized by community type II. Furthermore, several lines of evidence also support the microbiome shift from community type II to type I during COVID-19 (Figs. 1e, 2e, 3a, and 4). We revised related sections in the revised MS. In spite of this, we believe that the alternations and dynamics changes of microbiome caused by COVID-19 are divergent among different individuals (please see New supplementary Fig. S6 in page 6)

-Line 124-125: "These results indicate that the change of the respiratory microbiome might be closely associated with disease progression in COVID-19". The strength of this statement could be substantially improved by matching community types to some measure of clinical severity (e.g. symptom score, viral load, oxygen saturation, etc.) if this data is available.

Authors: Thank you for this suggestion. We performed this analysis using available clinical parameters (e.g. total T lymphocyte, B lymphocyte, NK cells, CD4/CD8 ratios, etc.) (new supplementary Fig. S10: below). These results showed that there was no significant association between the microbiome diversity and clinical parameters except that upper respiratory microbiome richness appeared to negatively correlate with NK cell counts.

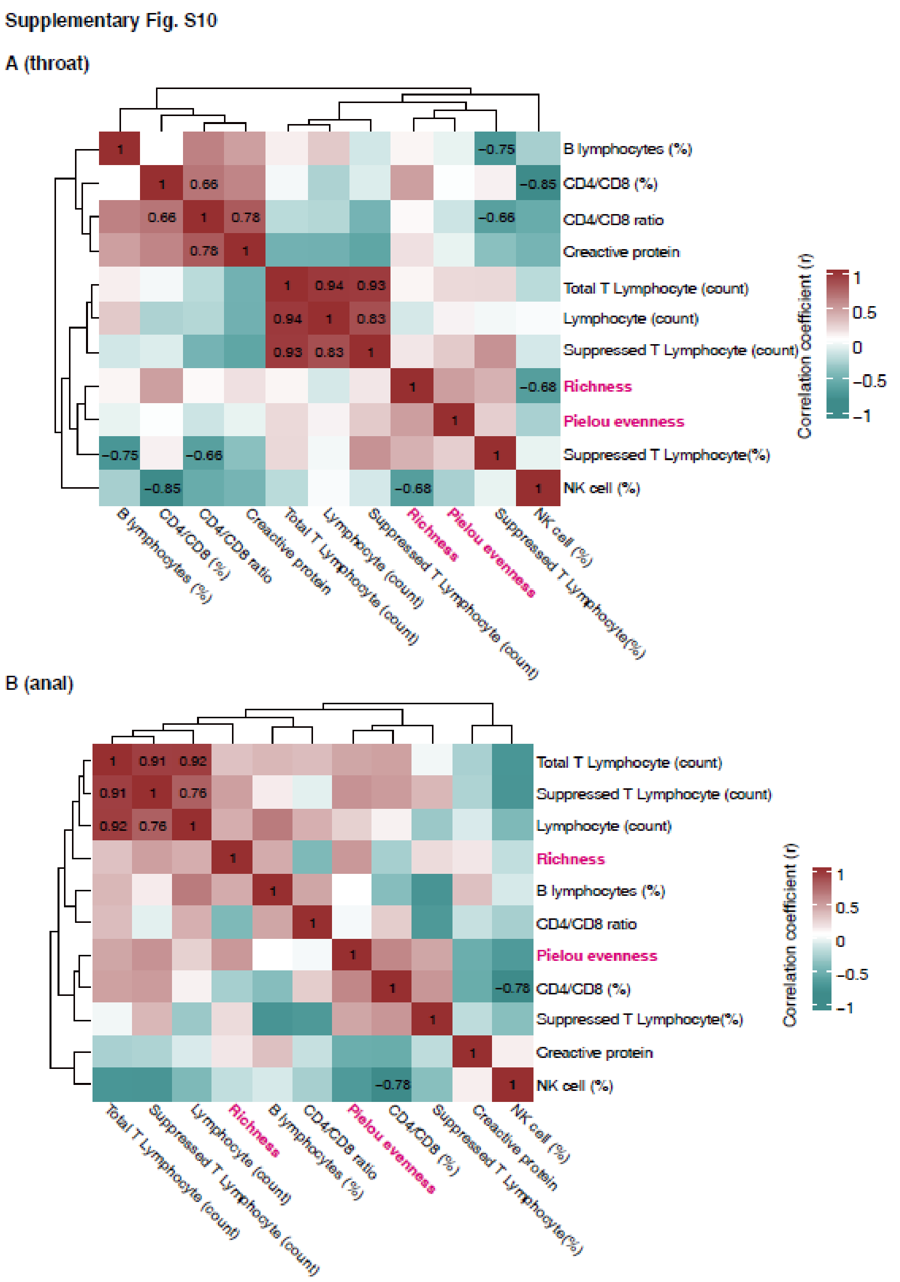

New supplementary Fig. S10. Correlation of microbiome diversity with clinical parameters.

-Line 161-163: Could the lack of correlation between community divergence and other parameters be due to the relatively small sample size available?

Authors: Relatively small sample size might be a reason for weak or no correlation between community diversity and clinical parameters (new supplementary Fig. S10: above).

-Line 182-184: Authors discuss the hypothesis that co-occurrence networks between bacteria reflect crosstalk between the gut and lungs as a result of bacterial translocation, which is later described as a result of damage to the respiratory and gastrointestinal mucosa (Lines 194-195; Lines 261-262). Other processes may also explain these co-

occurrences including (1) bacteria migrating from the oropharangeal site to the gut via swallowing and passage through the gastrointestinal tract (especially as several oral taxa are implicated) or (2) induction of immune responses at both sites which apply similar selective pressures to the microbiota. Could the authors please address these hypotheses.

Authors: Thank the reviewer for these suggestions. We discussed these potential mechanisms.

-Similarly, is there any evidence available for a breakdown of mucosal barriers which may support the hypothesis of the authors (e.g. serum LPS or citrulline, etc.)? This may not be possible given the status of the patients but could provide valuable support for the hypothesis.

Authors: Thank you for this suggestion. We measured the serum LPS levels of the COVID-19 patients. High levels of serum LPS were found in some patients such as P05, P09, P10, P13, etc, implying disruption of mucosal barriers and potential microbial translocation. In spite of high LPS levels in some patients, our data are more consistent with the interpretation that a cross-talk between the respiratory and gut microbiomes occurred more likely through respiratory and gastrointestinal tracts. Furthermore, we performed the correlation analysis, and found that the microbial richness index was negatively correlated with the serum LPS levels (New supplementary figure S11).

Supplementary Fig. S11

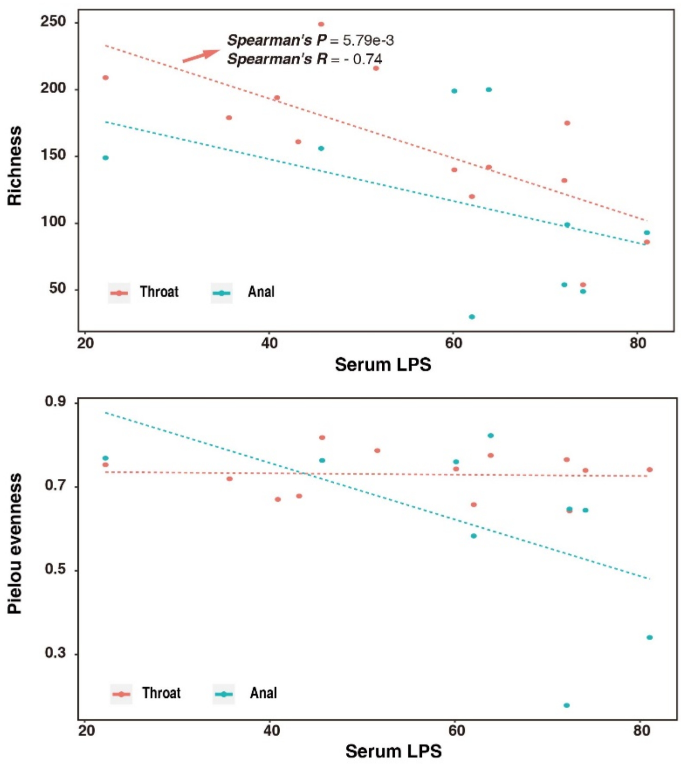

New supplementary Figure S11. Correlation between microbiome diversity and LPS levels.

-Line 202-204: Increased *Bifidobacterium* and *Faecalibacterium* in throat samples were noted as evidence of restoration in microbiome composition, yet to the best of my knowledge these genera are not considered to be a normal part of the oropharangeal microbiome. Can the authors please provide some citations to demonstrate that these genera are representative of a healthy oropharangeal microbiome.

-Increases in *Bifidobacterium* and *Faecalibacterium* are noted as signs of improvement and restoration (e.g. Lines 164-170; 202-204) but the authors note several other commensal genera such as *Bacteroides*, *Roseburia, Blautia*, and *Coprococcus*, while other probiotic strains such as Lactobacillus are also prominent members of the microbiota. Can the authors explain why they chose to focus on *Bifidobacterium* and *Faecalibacterium* specifically, and not other probiotic genera? If other genera do not display similar patterns, does this perhaps indicate that the restoration of the microbiota is incomplete and may have long-term consequences?

Authors (to both questions above)**:** *Bacteroides*, *Roseburia*, *Blautia*, *Coprococcus*, *Faecalibacterium, Lactobacillus* and *Bifidobacterium* are the core functional bacteria of gut (Falony G, Joossens M, Vieira-Silva S, Wang J, Darzi Y, Faust K, Kurilshikov A, Bonder MJ, Valles-Colomer M, Vandeputte D, Tito RY, Chaffron S, Rymenans L, Verspecht C, De Sutter L, Lima-Mendez G, D'hoe K, Jonckheere K, Homola D, Garcia R, Tigchelaar EF, Eeckhaudt L, Fu J, Henckaerts L, Zhernakova A, Wijmenga C, Raes J. Population-level analysis of gut microbiome variation. Science. 2016 Apr 29;352(6285):560-4.). In the revised MS, the top indicator bacteria with indicator values above 0.5 were selected from each cluster (Fig. 1d and 2d), and were subjected to the analyses together with the several core functional bacteria (e.g. *Faecalibacterium, Lactobacillus* and *Bifidobacterium*) in gut.

Although *Bifidobacterium* and *Faecalibacterium* are not the normal part of the oropharangeal microbiome, the majority of the species of *Bifidobacterium* and *Faecalibacterium* are documented to be beneficial bacteria and can be used as probiotics. In fact, the relative abundance of *Bifidobacterium* and *Faecalibacterium* was indeed substantially lower in the upper respiratory tract than the gut (Fig. 3b), supporting that *Bifidobacterium* and *Faecalibacterium* are not the most common bacteria in oropharangeal microbiome. Although the increase in *Bifidobacterium* and *Faecalibacterium* in throat samples might not be noted as evidence of restoration of microbiome composition in upper respiratory tract, their increase in gut at least reflects the improvement and restoration of gut microbiome.

Several parameters, including α-diversity, bacterial community types, and representative bacteria, were used to reflect the restoration of the microbiota. According to our current results, the microbiotas of COVID-19 patients have not been restored to healthy type (Fig. 1c). Therefore, we presume that COVID-19 has a long-term

consequence on health and deserve further follow-up investigation.

-Line 228-230: The authors indicate that inconsistencies in changes observed during longitudinal sampling indicate that diversity characteristics of the throat microbiome were affected by COVID-19. Can the authors please explain why they determined this direction of causality (i.e. is it possible that diversity characteristics may have rather influenced the progression of COVID-19 and not vice versa).

Authors: Although it is difficult to draw a solid conclusion on the causality between SARS-CoV-2 infection and the altered microbiota, according to our results (comparison with the microbiota of healthy controls in diversity, community types and indicator bacteria) and previous studies (Zuo T, et al. Alterations in Gut Microbiota of Patients With COVID-19 During Time of Hospitalization. Gastroenterology. 2020 Sep;159(3):944-955.e8.; Gu S, et al. Alterations of the Gut Microbiota in Patients with COVID-19 or H1N1 Influenza. Clin Infect Dis. 2020 Jun 4:ciaa709.), it is more likely that SARS-CoV-2 infection first alters the microbiota in respiratory tract and the gut, and then the changed microbiota leads to a long-term influence on the health of these patients.

In fact, the relationship between respiratory virus infection (e.g. influenza, RSV, rhinovirus, etc.) and microbiota has been investigated (Dubourg G, Edouard S, Raoult D. Relationship between nasopharyngeal microbiota and patient's susceptibility to viral infection. Expert Rev Anti Infect Ther. 2019 Jun;17(6):437-447). In this study, we provided further evidences to support the interaction between respiratory virus infection and microbiota. In particular, the dynamic changes of microbiota in both upper respiratory tract and gut over time since SARS-CoV-2 infection provide new insight into the understanding of mechanism of COVID-19 and the causality of virus infection and microbiota.

-Line 268-272: It is stated that the gut microbiome appeared to have a faster restoration to increased bacterial diversity, but this phenomenon did not appear to be described in the results section. Indeed, Figure 3a seems to suggest that the progression to more diverse community types was occurring at the same time in the two body sites. Could the authors please provide clarity for the justification of this statement.

-Similarly, in Lines 270-272 the authors describe bacterial crosstalk which promotes restoration of the respiratory microbiota. How do the authors propose that bacteria are translocated from the gut to the oropharynx, particularly if mucosal integrity is beginning to recover concurrent with restoration of the microbiome?

Authors (to both questions above)**:** We agree that the progression to more diverse community types was occurring at the same time in the upper respiratory tract and gut. Therefore, we corrected previous description into “synchronous occurrence” in the

revised MS.

The co-occurrence networks analysis suggests the cross-talk between the respiratory and gut microbiomes (new Fig. 5). In fact, the detection of *Bifidobacterium* and *Faecalibacterium* (the core functional bacteria in gut) in the upper respiratory tract in spite of lower relative abundance might support the cross-talk. Although we also detected high level of serum LPS in some COVID-19 patients (supplementary Table S2), we presume that the cross-talk between upper respiratory and gut microbiomes might be mediated through oropharyngeal-gastrointestinal tracts or diet.

Minor comments:

-Title should be revised to make it clear that this work specifically investigated bacterial communities, and not the broader microbiome (including viruses, archaea and fungi)

Authors: We emphasized "bacterial" microbiomes in title and some places of the main text.

-Line 30: It may not be accurate to say that this paper "addresses the question" of whether microbiomes "affect disease progression". Although the authors show longitudinal changes, they do not demonstrate causality or a functional role and should be cautious of over-emphasising their results

Authors: We agree with reviewer. We edited related sentences and accordingly toned down some conclusions in the revised MS.

-Line 36: replace "they had a" with "their

-Line 61: delete "but"

-Lines 49-55: Sentence is long and difficult to follow. Consider breaking it up into smaller sentences

-Please avoid using "significantly" where statistical analysis has not been applied (e.g. line 122 refers to a change in a single sample). Consider perhaps using "substantially" or a similar word to emphasis the magnitude of changes.

-Line 210: reference to "near-normal microbiota" may be difficult to define, especially in the gut samples where no samples were available from healthy controls. Perhaps rephrasing to "more diverse" or "greater abundance of commensals" may be a more accurate description

-Line 233: please change "baseline" to "early infection" or similar, to avoid any ambiguity about whether the samples were collected prior to infection.

-Line 306: please change "will be particularly useful" to "may be useful" as SCFA production is influence by factors other than microbiome composition (e.g. diet, cross-feeding, etc.)

-Line 303 and 310: Please capitalise Pseudomonas

Authors (to eight questions above)**:** Thank you very much for these editing suggestions. We did all these changes in the revised MS.

-descriptions of "bacterial interactions" (e.g. line 36, line 193, etc) may be interpreted to mean that the authors have evidence of bacterial cells interacting, whereas the analysis performed only demonstrated correlations and co-occurrence. Revised word should be considered to avoid any confusion

Authors: We changed "interactions" into "co-occurrence".

-Line 166: The authors state that that relative abundances of commensals "appeared to be" correlated. This term is ambiguous – if a correlation was statistically significant then the result should be stated more conclusively. If it was merely identified as an association or trend then the term "correlated" should be avoided as this is a specific type of statistical analysis.

Authors: We changed "correlated" into "associated".

-Line 217-219: authors describe that the respiratory microbiome is more easily affected by the infection, but also admit that this has not been examined yet. The first half of this sentence should be rephrased to demonstrate this is a hypothesis and not a known phenomenon

Authors: The altered respiratory microbiome was previous observed during other respiratory virus infections (e.g. RSV, influenza etc.). So, we changed the sentence as "As an open system with direct contact with environment and the primary site for respiratory infections, the respiratory tract microbiota is more easily affected." However, the effect of SARS-CoV-2 infection has not been examined".

-please describe how "mild" and "severe" cases of COVID-19 were distinguished in patients

Authors: We added the definitions and criteria of mild" and "severe" cases of COVID-19 into the Methods section.

-Line 265: it seems contradictory to state that the gut microbiome is both "more stable" and "more plastic: than the respiratory microbiota. Please clarify

Authors: We used "plasticity" to express resilience of the microbiota. To avoid confusion, we replaced "plastic" by using "resilient".

Temporal association between human upper respiratory and gut bacterial microbiomes during the course of COVID-19 in adults

Running title: Upper respiratory and gut microbiomes in COVID-19 adults

Rong Xu[1, 4, #], Renfei Lu[2, #], Tao Zhang[3, #], Qunfu Wu[3, #], Weihua Cai[2], Xudong Han[2], Zhenzhou Wan[5], Xia Jin[1] *, Zhigang Zhang[3] *, Chiyu Zhang[1,4] *

1. Shanghai Public Health Clinical Center, Fudan University, Shanghai 201508, China
2. Clinical Laboratory, Nantong Third Hospital Affiliated to Nantong University, Nantong 226006, China
3. State Key Laboratory for Conservation and Utilization of Bio-Resources in Yunnan, School of Life Sciences, Yunnan University, Kunming, Yunnan 650091, China
4. Pathogen Discovery and Evolution Unit, Institut Pasteur of Shanghai, Chinese Academy of Sciences, Shanghai 200031, China.
5. Medical Laboratory of Taizhou Fourth People's Hospital, Taizhou 225300 China

# These authors contributed equally to this work.

* Correspondence: Chiyu Zhang, Shanghai Public Health Clinical Center, Fudan University, Shanghai 201508, China. Email: zhangcy1999@hotmail.com
Or Zhigang Zhang, State Key Laboratory for Conservation and Utilization of Bio-Resources in Yunnan, School of Life Sciences, Yunnan University, No. 2 North Cuihu Road, Kunming, Yunnan 650091, China. Email: zhangzhigang@ynu.edu.cn.
Or Xia Jin, Shanghai Public Health Clinical Center, Fudan University, Shanghai 201508, China. Email: jinxia@shphc.org.cn

Abstract

SARS-CoV-2 is the cause of COVID-19. It infects multiple organs including the respiratory tract and gut. Dynamic changes of regional microbiomes in infected adults are largely unknown. Here, we performed longitudinal analyses of throat and anal swabs from 35 COVID-19 and 19 healthy adult controls, as well as 10 non-COVID-19 patients with other diseases, by 16S rRNA gene sequencing. The results showed a partitioning of the patients into 3-4 categories based on microbial community types (I-IV) in both sites. The bacterial diversity was lower in COVID-19 patients than healthy controls and decreased gradually from community type I to III/IV. Although the dynamic change of microbiome was complex during COVID-19, a synchronous restoration of both the upper respiratory and gut microbiomes from early dysbiosis towards late more diverse status was observed in 6/8 mild COVID-19 adult patients. These findings reveal previously unknown interactions between upper respiratory and gut microbiomes, and suggest that modulations of regional microbiota might help to improve the recovery of COVID-19 patients.

Keywords: SARS-CoV-2/COVID-19; upper respiratory microbiota; gut microbiota, dysbiosis; adults; co-occurrence network.

Introduction

COVID-19, a severe respiratory disease caused by a novel virus SARS-CoV-2[1,2], has led to a devastating global pandemic. It typically presents as an asymptomatic infection or manifests mild respiratory symptoms, but in elderly over 60 years of age or those having comorbidities, COVID-19 can develop into severe pneumonia and cause death[3,4]. The biological mechanisms behind the varied clinical presentations are not fully understood.

The microbiota plays a major role in modulating human health status by shaping the immune system and maintaining homeostasis[5]. In several respiratory viral infections (RVs), the microbial composition in the respiratory tract and the gut have been linked to the occurrence and severity of disease and affects subsequent respiratory health[6,7], through increasing airway susceptibility to infection by other RVs and/or the colonization of pathogenic bacteria[8-10]. It is therefore reasonable to posit that the new respiratory infection COVID-19 may also interact with microbiota.

Indeed, some recent studies have shown that SARS-CoV-2 infects human gut enterocytes and causes diarrhea[11,12]. Altered gut microbiota has been observed in COVID-19 patients leading to an enrichment of opportunistic pathogens and a depletion of beneficial bacteria[13,14]. However, changes in the respiratory microbiome has not been evaluated in COVID-19. Furthermore, despite persistent alterations in the gut microbiota has been reported using longitudinal stool samples collected in COVID-19 patients[13], no study has examined whether there is any association between the respiratory and gut microbiota during the cause of disease. In this study, we investigated for the first time the dynamics of both the upper respiratory and gut microbiomes in a cohort of COVID-19 patients and controls, and discovered a pattern of synchronous changes in these two microbiomes ~~communities~~.

Results

Study cohort

The study subjects included 35 adult COVID-19 patients from 17 to 68 years of age, 19 healthy adults, and 10 non-COVID-19 patients (NP) with other diseases. Except patient p09 who had severe clinical symptoms, all other 34 COVID-19 patients had mild clinical symptoms. A total of 146 specimens including 37 pairs of both throat and anal swabs were collected from COVID-19 patients (Supplementary Fig. S1). High-throughput sequencing of the V4-region of bacterial 16S rRNA gene was performed for all samples.

Respiratory microbiome dynamics in COVID-19

The 16S-rRNA gene sequences of all throat swabs were resolved into 3,126 amplicon sequence variants (ASVs) representing 17 known phyla including 209 known genera (Supplementary Table S1). Six throat microbial community types (or clusters) were identified using the Dirichlet Multinomial Mixtures (DMM) modelling based on the lowest Laplace approximation (Fig. 1a) and visualized by Nonmetric Multidimensional Scaling (NMDS) based on Bray-Curtis distance (Fig. 1b). Thirteen of 19 specimens of healthy adults (H) formed an independent cluster defined as community type H. The vast majority of the specimens of COVID-19 patients were divided into four clusters, herein named community types I-IV (Fig. 1a). Other specimens from 6 COVID-19 patients were clustered with those from 10 non-COVID-19 patients and two healthy controls. Because this cluster has a significantly higher proportion of NP patients (55.6%, $P<0.01$) than COVID-19 patients (33.3%) and healthy controls (11.1%) (Supplementary Fig. S2), it was designated as community type NP. All COVID-19-related community types, as well as the NP type, were significantly distant from the H type. Community types III and IV were not only separated from the types I and II, but also from each other (Fig. 1b). A decrease in alpha-diversity of the microbiome was observed from type I to IV, and significantly lower richness and evenness were observed in community types III and IV, compared with the H type (Fig. 1c). Similar decreasing trends of alpha-diversity were also observed when the Margalef's indexes were used to control the effect of sample size (Supplementary Fig. S3)[15].

To more directly demonstrate that the variation of throat microbial composition is an indicator of COVID-19 disease stages, the community type-specific indicator taxa were identified based on the top 30 microbial genera (Fig. 1d). The type H was characterized by bacterial genus *Bacteroides*

(predominant taxa in the lung of healthy individuals) and unclassified *Comamonadaceae*, whereas the NP type was marked by pro-inflammatory *Enterobacteriaceae* members. In contrast, the indicator bacteria of four COVID-19-related community types were *Alloprevotella* in type I, *Porphyromonas*, *Neisseria*, *Fusobacterium* and unclassified *Bacteroidales* in type II, *Pseudomonas* in type III, and *Saccharibacteria incertae sedis*, *Rothia* and unclassified *Actinomycetales* in type IV (Fig. 1d). Community type I contained *Alloprevotella* genus, as well as abundant *Bacteroides* and *Prevotella* that typically present in the H type (Fig.1a). Some indicator bacteria substantially enriched in types II and IV belong to opportunistic pathogenic bacteria that may be associated with human diseases such as pneumonia, chronic periodontitis, and bacteremia [16-21]. For example, the identified *Rothia* species in type IV have the highest sequence similarity with *Rothia mucilaginosa* that is often associated with cancer and bacteremia[22]. *Porphyromonas*, *Fusobacterium,* and *Neisseria* enriched in type II typically exist in the nasopharynx, and they are associated with pneumonia or chronic periodontitis. Besides opportunistic pathogenic bacteria, commensals (e.g. Bacteroidales) were also enriched in type II. In type III, the identified *Pseudomonas* species have the highest sequence similarity (100%) with the non-pathogenic species *Pseudomonas lactis* that was initially isolated from bovine raw milk, and rarely found in human[23]. Compared with the community type H, a decreased alpha-diversity with high abundance of opportunistic pathogenic and environmental bacteria (*Pseudomonas lactis*) in community types II-IV might imply a disruption of microbiome homeostasis (dysbiosis) in the respiratory tract (Supplementary Table S1 and Fig. S4). Lower alpha-diversity with enrichment of pro-inflammatory *Enterobacteriaceae* indicates that the type NP represents another status of microbial dysbiosis.

According to indicator bacteria and alpha-diversity characteristics, the microbial community types from I to IV may represent a progressive imbalance of the respiratory microbiome (Fig. 1c-d). Among all throat specimens from COVID-19 patients, 47 (56.6%) belong to community type II (Supplementary Fig. S2), indicating that altered upper respiratory microbiome by COVID-19 was mainly characterized by community type II. Longitudinal analysis showed that community types with relatively lower alpha-diversity are more likely to have appeared in early specimens (Fig. 1e), but the diversity did not significantly correlate with the time after symptom onset regardless of being analyzed at the overall and individual levels (Supplementary Fig. S5-S6). Among 22 COVID-19 adults who had specimens at two or more time points, over half (12, 54.5%) maintained a relatively

stable microbiome community types, and the others had community types altered over time. An obvious throat microbiome recovery from types IV or II in early specimens to type I in late specimens was observed in five patients (p17, p25, p13, p11 and p05) with 4 or more consecutive specimens (Fig. 1e), accompanied with the restoration of throat microbiota, appearance of beneficial commensals, and increased bacterial diversity (Supplementary Fig. S4). Conversely, an opposite pattern was observed in four patients who had microbiome composition shift from early higher-diversity types (I or II) to later lower-diversity type (II-IV), implying a worsening of the throat microbiome. In particular, the only severe case (p09) experienced a community type shift from type I on day 10 to type IV on day 27, and sustained type IV to at least day 33 after symptom onset (Fig. 1e). Accompanied with this shift, opportunistic pathogenic bacteria *Saccharibacteria incertae sedis* and *Rothia* were substantially enriched at late stage (Supplementary Fig. S4). These indicate that the dynamic changes of upper respiratory microbiome caused by COVID-19 was heterogenous among different individuals.

Gut microbiome dynamics in COVID-19

To expand the scope of this research, a total of 1,940 ASVs were recovered from the 16S-rRNA gene sequences of all anal swabs, representing 13 known phyla including 182 known genera (Supplementary Table S1). The gut microbial communities of COVID-19 patients formed three distinct community types I-III (Fig. 2a-b). The richness and evenness of the gut microbiome decreased from type I to III (Fig. 2c). Indicator analyses showed that type I was primarily characterized by healthy gut bacteria including *Bacteroides* genus and several known butyrate-producing bacteria (e.g. *Faecalibacterium*, *Roseburia*, *Blautia*, and *Coprococcus*) and one opportunistic pathogenic bacterium (*Finegoldia*) (Fig. 2d)[24-29]. The indicators of type II mainly contain various pathogenic or opportunistic pathogenic bacteria (e.g. *Neisseria* and *Actinomyces*). In community type III, the gut microbiota was dominated by *Pseudomonas*, implying a severe dysbiosis. We also used the community types I-III to examine the dysbiosis status of the gut microbiome.

A shift of the gut microbiome from the lower-diversity community type (II or III) towards a higher-diversity type (I or II) was observed over time in 8/10 patients who had anal swabs at different time points (Fig. 2e). Accompanied with the shift, a clear trend of increased bacterial diversity and the relative abundance of beneficial commensals (e.g. *Bacteroides* and *Faecalibacterium*) was

observed in the gut microbiota from early to late stages of COVID-19 (Supplementary Fig. S7), suggesting a restoration of gut microbiota. Two patients maintained a stable microbiome community types, and only one patient had an opposite shift of community type from higher-diversity type II to lower-diversity community type III.

Association between the respiratory and gut microbiomes in COVID-19

Most paired throat and anal swabs showed the same or similar community type levels (Fig. 3). In particular, the shift of microbiome community types over time appeared to match between the throat and the gut in 7/8 patients who had two or more paired specimens at different time points (Fig. 3). Synchronous improvement of both the respiratory and gut microbiomes from early lower-diversity community type towards late higher-diversity type occurred in six patients (p05, p17, p13, p11, p25 and p29). One patient (p33) experienced an improved respiratory microbiome but maintained an unchanged gut community type up to day 24. One case (p07) had a worsen gut microbiome from day 24 to day 35 but maintained an unchanged respiratory community type. Because of no available anal specimens, we were unable to assess whether the gut microbiota, like the respiratory microbiota, shifted from higher-diversity type to lower-diversity type over time in the severe case (p09) (Fig. 1e). Except for the duration of COVID-19, the upper respiratory and gut microbial community divergence seemed not to be significantly associated with age, gender, antibiotics use, and detection of SARS-CoV-2 RNA (Supplementary Figs. S8-S9). The alpha-diversity of the microbiome was also not significantly associated with the time after symptom onset (supplementary Fig. S5-S6), and clinical parameters, except for a weak association between the upper respiratory microbiome richness and NK cell counts (Supplementary Figs. S10). Furthermore, the richness of both upper respiratory and gut microbiome appeared to be negatively correlated with the serum levels of lipopolysaccharides (LPS) (Supplementary Fig. S11 and Table S2).

We further selected the top indicator bacteria with > 0.5 indicator values from each community type (Figs. 1d and 2d) and several major core functional bacteria (e.g. *Faecalibacterium*, *Lactobacillus* and *Bifidobacterium*) in gut as the representative bacteria to assess their dynamic changes in relative abundance over time (Fig. 4). In general, the relative abundances of *Bifidobacterium*, *Lactobacillus* and/or *Faecalibacterium* appeared to be negatively associated with the relative abundance of the opportunistic pathogens (e.g. *Rothia* and *Neisseria*), especially in the

gut microbiome. An obvious decrease in the relative abundance of opportunistic pathogenic bacteria was accompanied by an increase in the relative abundance of resident commensals *Bacteroides* in gut microbiome over time in five patients having three or more longitudinal samples (Fig. 4 and Supplementary Fig. S4 and S7). Moreover, a substantially decreased abundance of *Pseudomonas* was observed in both organs in another two patients (p23 and p29). The relative abundance of *Pseudomonas* increased only in patient (p07) who experienced a worsening gut microbiome.

Bacteria–bacteria co-occurrence networks

There were four indicator bacteria genera (*Porphyromonas*, *Neisseria,* and *Fusobacterium* in type II and *Pseudomonas* in type III) in the throat microbiome that had been identified as the indicators of gut microbial community types II and III in COVID-19 patients (Supplementary Fig. S12). Apart from the shared indicators, oropharyngeal pathogenic bacteria *Capnocytophaga* and *Actinomyces* were also identified as indicators of the gut microbial community type II (Figs. 1d and 2d)[30,31]. Because community types II and III often appeared in the early stage of COVID-19 (Figs. 1e and 2e), the appearance of these oropharyngeal bacteria in the gut suggested that a cross-talk between the respiratory and gut microbiomes occurred by frequent bacterial translocation during the early stage. Furthermore, high serum LPS levels were detected in some COVID-19 patients (Supplementary Table S2), suggesting the possibility of bacteria translocation.

To further investigate the association between the respiratory and gut microbiomes, we performed co-occurrence network analysis using paired specimens from 13 patients. We constructed a co-occurrence network consisting of a total of 153 co-occurred pairs with Pearson correlation $|r| > 0.7$ under FDR-adjusted $P < 0.05$ (Fig. 5). Bacteria in the same niche trended to have close co-occurrence relationship, and the cross-talks of microbial compositions between the upper respiratory tract and the gut were also observed. In particular, a competitive relationship between Gut-type-II and Gut-type-I was mediated by a significantly negative interaction between gut bacterial genera *Neisseria* and *Bacteroides* (Fig. 5), which might determine the microbiome shift from Gut-type-II to Gut-type-I during the COVID-19 disease progression. Furthermore, core resident commensals *Bacteroides* appeared to mediate the cross-talk between Throat-type H and Gut-type-I, which might modulate the restoration of throat and gut microbiota during COVID-19[27,32].

Discussion

Whether SARS-CoV-2 infection alters microbiota to affect COVID-19 disease progression is an important question that needs answers. In this study, we made three major observations. First, the upper respiratory and gut microbiota compositions of COVID-19 adults can be characterized by four (I- IV) and three (I-III) community types, respectively, and these types reflect different levels of balance between the more diverse microbiota (type I) and dysbiosis (type II-IV). Second, upper respiratory and gut microbiome altered by COVID-19 are mainly characterized by community type II, and the microbiome community types with lower alpha-diversity more likely appears in the early phase of COVID-19. Third, the dynamic change of community types is synchronous in the upper respiratory tract and gut.

SARS-CoV-2 infects cells through ACE2 receptor[2], which is highly expressed in respiratory and intestinal epithelial cells[33]. The infection can trigger the cytokine storm, cause local pathological damage[34,35]. As an open system with direct contact with environment and the primary site for respiratory infections, the upper respiratory tract microbiota is more easily affected by respiratory virus infections, but the effect of SARS-CoV-2 infection has not been examined yet. In this study, we observed alterations of the upper respiratory microbiota in COVID-19 adults, and presented data on the dynamic change of the respiratory microbiome composition over time. The upper respiratory microbiome of the COVID-19 adults was characterized by four bacterial community types I-IV, which reflect the different levels of the normal microbiome to dysbiosis. The community types with lower alpha-diversity and high enrichment of opportunistic pathogenic bacteria and *Pseudomonas* often appeared in early throat specimens (e.g. first several days after symptom onset), indicating that SARS-CoV-2 infection results in a very rapid dysbiosis in upper respiratory tract. A restoration of the upper respiratory microbiome from dysbiosis towards more diverse types was observed over time in some with mild disease, whereas prolonged or worsening microbiome appeared in a few others including the only one severe case (p09).

Intestinal enterocytes that express ACE2 are also the target of SARS-CoV-2 which further up-regulates the expression of ACE2, leading to a longer viral RNA shedding time in the gut than respiratory tract[11,33]. The early infection microbiome composition with abundant pathogenic bacteria (e.g. *Coprobacillus*, *Clostridium ramoaum* and *Clostridium hathewayi*) had been associated with the fecal levels of SARS-CoV-2 and COVID-19 severity in a previous study[33]. However, the sampling

time was relatively late in that study (about 14 days after symptom onset), therefore unable to determine whether the early infection microbiome status is a consequence of SARS-CoV-2 infection, or a cause of disease severity. We also observed alterations of the gut microbiota during COVID-19 in adults, and found some opportunistic pathogenic bacteria (e.g. *Streptococcus*, *Rothia*, *Veillonella*, *Actinomyces* and *Actinomyces*) reported in the previous observations[13,14]. However, distinct from the previous studies, we identified three community types (I-III) that can characterize the changes of gut microbiome over time. Similar to the observation in the upper respiratory microbiome, community types (i.e. II and III) with lower alpha-diversity often appeared in early specimens, supporting the early effect of SARS-CoV-2 on the gut microbiome. A restoration with the community type shifted from low-diversity type II to high-diversity type I over time was observed in at least 4 patients. However, *Pseudomonas*-dominated community type III showed a slow improvement towards community type II in three patients. In particular, the temporal dynamic changes of the microbiomes matched between the upper respiratory tract and the gut, indicating a close association in microbiota between both body sites, possibly via the “airway-gut axis”[36].

The reason for the fast dysbiosis in both the upper respiratory tract and the gut of COVID-19 patients might be associated with the early-stage inflammation induced by SARS-CoV-2 infection, which leads to a fast loss of beneficial commensals, and the colonization and growth of opportunistic pathogenic bacteria (Supplementary Fig. S13). The use of empirical antibiotics in some patient during the early stages of the pandemic may exacerbate the dysbiosis in the upper respiratory tract and gut. Therefore, the microbiome composition with enrichment of opportunistic pathogenic bacteria (e.g. *Rothia* and *Neisseria*) was observed in both throat and gut microbiomes during the first several days after symptom onsets. Because the upper respiratory tract is more receptive to both exogenous and indigenous microbes than the gut[7,37], the dysbiosis of upper respiratory microbiome appeared to be worse and occurred earlier than that of the gut microbiota, as manifested by lower diversity and richness and more indicators of opportunistic pathogenic bacteria in the former than in the latter. The damaged upper respiratory tract mucosa enables some oral taxa to be translocated to the gut, worsening the gut bacterial community (Supplementary Fig. S13). There are several possible mechanisms to explain the oropharyngeal bacterial translocation to the gut. First, inflammation induced by SARS-CoV-2 infection damaged the mucosal tissues and increased mucosal permeability of the airway, lung and gut[7,38], which then enables bacterial translocation. Second, bacteria migrated

from the oropharyngeal site to the gut via swallowing and passage through the gastrointestinal tract. Third, immune responses induced by infection applied similar selective pressures to the microbiota at both sites.

Gut microbiota plays an important role in human health by shaping local immunity and remodeling mucosal tissues[39]. It is relatively more stable and resilient than the respiratory microbiota, and it may affect the latter by cross-talk between these two organs along the airway-gut axis[36,37]. In spite of longer duration of SARS-CoV-2 shedding in the gut than in the respiratory tract, gut microbiota appeared to have a synchronous change with the respiratory microbiota (Fig. 3). Although the dynamic change of the microbiome was relatively divergent and independent of early microbiome community types, synchronous restoration of both the respiratory and gut microbiomes from early low diverse status towards late more diverse status was observed in 6 (75%) mild COVID-19 adult patients who had two or more paired specimens at different time points. Age, gender and antibiotics use seemed not to be linked to restoration of the microbiome, implying potential contributions from other factors such as diet and genetic background.

As common bacteria in bovine raw milk, *Pseudomonas* was rarely detected in human[23]. Because of specific antibiotics, it was not surprising that *Pseudomonas*-dominated bacterial community type III was difficult to restore towards higher-diversity community types in the upper respiratory tract. The identification of some opportunistic pathogenic bacteria (*Neisseria, Porphyromonas, Rothia, Actinomycetales* and *Saccharibacteria*) in more dysbiosis community types II and IV might imply a need for microbiota-based personalized antibiotics treatment against these specific pathogens. As the most common microbiome status in COVID-19 patients, the community type II represents a crucial intermediate stage during the restoration of the microbiome from dysbiosis towards more diverse microbiome. It was characterized by *Neisseria, Fusobacterium,* and *Porphyromonas*. *Fusobacterium*. *Porphyromonas* are the common commensals in the oropharynx and the gut[19,21], while *Neisseria* generally presents in the lung. The appearance of lung *Neisseria* in both the upper respiratory tract and the gut, implying bacteria translocations along the "airway-lung-gut axis"[40]. The bacteria translocations may be the consequence of increased permeability among these organs caused by local inflammation[41], as evidenced by high levels of serum LPS. The *Bifidobacterium* and some butyrate-producing bacteria (e.g. *Faecalibacterium*) can improve the inflammatory conditions and regulate innate immunity by down-regulating ACE2 expression, and activating the corresponding signaling

pathways[27,32]. During the restoration of the microbiota, these beneficial bacteria gradually occupied the ecological niches in the gut and respiratory tract, and governed the microbial communities in both organs by replacement of opportunistic pathogenic bacteria (e.g. *Rothia* and *Neisseria*) over time. However, a progressively worsening in the upper respiratory and gut microbiome might be associated with severe cases of COVID-19.

One noted limitation of this study is the relatively small patient number. Our results may not be representative of all patient groups, and the observed dynamic changes of the microbiome in both the upper respiratory tract and gut may be further validated in a larger cohort. Another limitation of the study is that the dynamic changes of the microbiome were only followed up to 35 days after symptom onset. Whether COVID-19 exerts long-term effect of on the microbiomes is an interesting question for further investigation. Technically, the use of only 16S data may restrict our ability to identify specific bacteria species and infer their functions.

In summary, we revealed for the first time an association between the upper respiratory and gut microbiota during COVID-19 disease progression, and observed synchronous changes of microbiota in both organs mainly from early dysbiosis towards later more diverse status in a proportion of adults with mild COVID-19 (Supplementary Fig. S13). In the absence of specific antiviral drugs and vaccines for COVID-19, our findings may have clinical implications. For instance, some indicator bacteria (e.g. opportunistic pathogenic and beneficial butyrate-producing bacteria) can be used as crucial biomarkers for clinical treatment decision making and prognostic evaluation. The measurement of predominant short-chain fatty acid (especially butyrate) concentration in fecal samples may be useful in early clinical diagnosis. Apart from the routine treatment efforts (e.g. non-specific antiviral and supportive treatments)[42], precision intervention and modulation of the gut and respiratory microbiota may offer novel therapeutic alternatives, such as personalized antibiotics therapy to inhibit certain opportunistic pathogenic bacteria. Moreover, COVID-19 tailored probiotics (e.g. *Bifidobacterium* and *Faecalibacterium*), prebiotics (e.g. xylooligosaccharide) treatment, or symbiotic treatments might be applied to modulate the gut and respiratory microbiota to facilitate the recovery of COVID-19 patients.

Methods

Study population

A total of 64 subjects, including 35 laboratory-confirmed COVID-19 patients, 10 SARS-CoV-2 negative patients with various diseases (non-COVID-19) and 19 healthy adults were enrolled in this study. COVID-19 was diagnosed in adult patients according to the National Guidelines for Diagnosis and Treatment of COVID-19. The virus RNA was extracted from all samples using a Mag-Bind RNA Extraction Kit (MACCURA, Sichuan, China) according to the manufacturer's instructions. Then the *ORFlab* and *N* genes of SARS-CoV-2 was detected using a Novel Coronavirus (2019-nCoV) Real Time RT-PCR Kit (Liferiver, Shanghai, China) according to the manufacturer's instructions. Only the individuals who had at least two consecutive throat swabs been positive for both *ORFlab* and *N* genes of SARS-CoV-2 were defined as COVID-19 patients. All positive specimens of COVID-19 patients were confirmed by Nantong Center for Disease Control and Prevention (CDC) using recommended real-time RT-PCR assay by China CDC. Mild and moderate cases are defined as having clinical symptoms (e.g. fever, cough, etc.) with and without the pneumonia on lung imaging. Severe COVID-19 (adult) is defined as the presence of any one of the following: respiratory rate ≥30 breaths/minute, arterial oxygen saturation ≤93% at rest; PaO2/FiO2 ≤300 mm Hg. The COVID-19 patients were hospitalized at Nantong Third Hospital Affiliated to Nantong University. Among 35 COVID-19 patients, 34 were mild or moderate cases, and only one (P09) was severe case.

Demographic and clinical characteristics of the COVID-19 patients were provided in Supplementary Table S3 and S4[43]. Specimens including throat swabs and anal swabs were collected from the COVID-19 patients at different time points during their hospitalization (10-40 days). Sampling was performed using flexible, sterile, dry swabs, which can reach the posterior oropharynx and anus easily (approximately 2 inches) by the professionals at the hospital. At least two throat swabs at different days were available for 32 of 38 COVID-19 patients (Supplementary Fig S1).

Non-COVID-19 control patients were selected from patients hospitalized at the same hospital during the COVID-19 pandemic due to other diseases, and healthy controls were selected from adults who came for routine physical examination and showed no symptoms. Throat swabs of non-COVID-19 patients and healthy controls were collected during their hospital visit.

The study was approved by Nantong Third Hospital Ethics Committee (EL2020006: 28 February 2020). Written informed consents were obtained from each of the involved individuals. All

experiments were performed in accordance with relevant guidelines and regulations.

16S rRNA gene sequencing

Bacterial DNA was extracted from the swabs using a QIAamp DNA Microbiome Kit (QIAGEN, Düsseldorf, Germany) according to the manufacturer's instructions, and eluted with Nuclease-free water and stored at -80°C until use. The V4 hypervariable region (515-806 nt) of the 16S rRNA gene was amplified universal bacterial primers[44]. To pool and sort multiple samples in a single tube of reactions, two rounds of PCR amplifications were performed using a novel triple-index amplicon sequencing strategy as described previously[45]. The first round of the PCR (PCR1) amplification was performed with a reaction mixture containing 8 μL Nuclease-free water, 0.5 μL KOD-Plus-Neo (TOYOBO, Osaka Boseki, Japan), 2.5 μL of 1 μM PCR1 forward primer, 2.5 μL of 1 μM PCR1 reverse primer, and 5 μL DNA template. The products of the PCR1 reactions were verified using a 1.5% agarose gel, purified using Monarch DNA Gel Extraction Kit (New England Biolabs, Ipswich, MA, USA), and quantified by a Qubit® 4.0 Fluorometer (Invitrogen, Carlsbad, CA, USA). Equal amounts of purified PXR1 products were pooled, and subjected to the secondary round of PCR (PCR2) amplification. The PCR2 was performed with a reaction mix containing 21 μL Nuclease-free water, 1 μL KOD-Plus-Neo (TOYOBO, Osaka Boseki, Japan), 5 μL of 1 μM PCR2 forward primer, 5 μL of 1 μM PCR2 reverse primer, and 5 μL pooled PCR1 products. The PCR2 products were verified using a 2% agarose gel, purified using the same Gel Extraction Kit and qualified using the Qubit® 4.0 Fluorometer. The amounts of the specific product bands were further qualified by Agilent 2100 Bioanalyzer (Agilent, Santa Clara, CA, USA). Equal molars of specific products were pooled and purified after mixing with AMPure XP beads (Beckman Coulter, Pasadena, CA, USA) in a ratio of 0.8:1. Purified amplicons were paired-end sequenced (2x250) using Illumina-P250 sequencer.

Bioinformatic analysis of 16S rRNA gene sequence data

Sequenced forward and reverse reads were merged using USEARCH11 software[46], then de-multiplexed according to known barcodes using FASTX-Toolkit[47]. After trimming barcode, adapter and primer sequences using USEARCH11, 19,096,003 sequences were retained with an average of 105508 sequences per sample. Samples with sequence <1000 were excluded from the following analysis.

Because traditional OTU (operational taxonomic units) picking based on a 97% sequence similarity threshold may miss subtle and real biological sequence variation[48], several novel methods such as DADA2[49] and Deblur[50] were developed to resolve sequence data into single-sequence variants. Here, the DADA2 was employed to perform quality control, dereplicate, chimeras remove on Qiime2 platform[51] with default settings except for truncating sequence length to 250bp. Finally, an amplicon sequence variant (ASV) table, equivalent to OTU table, was generated and then spitted into gut ASV table (2348 ASVs) and throat ASV table (4050 ASVs). The taxonomic classification of ASV representative sequences was conducted by using the RDP Naive Bayesian Classifier algorithm[52] based on the Ribosomal Database project (RDP) 16S rRNA training set (v16) database[53]. To eliminate sequencing bias across all samples, both the gut ASV table and throat ASV table were subsampled at an even depth of 4700 and 3000 sequences per sample, respectively. The ASV coverage of 82.6% (gut) and 77.2% (throat) were sufficient to capture microbial diversity of both sites.

Identification and characterization of microbial community types

Dirichlet multinomial mixtures (DMM)[54] is an algorithm that can efficiently cluster samples based on microbial composition, its sensitivity, reliability and accuracy had been confirmed in many microbiome studies[55-57]. DMM clustering were conducted with bacterial genus abundance from throat and gut microbiota using the command "get.communitytype" introduced by v1.44.1 of mothur[58]. The appropriate microbial community type numbers (DMM clusters) were determined based on the lowest Laplace approximation index. According to sample counts per cluster, the fisher exact test was applied to discover significant associations between each cluster and host conditions (such as healthy controls, COVID-19 patients, and Non-COVID-19 patients) under P values that are below 0.05 adjusted by the False Discovery Rate (FDR). Conjugated with the Analysis of Similarities (ANOSIM), the reliability of DMM clustering was further validated and then visualized by the Non-metric multidimensional scaling (NMDS) based on the Bray-Curtis distance under bacterial genus level. "The ANOSIM statistic "R" compares the mean of ranked dissimilarities between groups to the mean of ranked dissimilarities within groups. An R value close to "1.0" indicates dissimilarity between groups, whereas an R value close to "0" indicates an even distribution of high and low ranks within and between groups". The ANOSIM statistic R always ranges between −1 to 1. The positive R values closer to 1 suggest more similarity within sites than between sites, and that close to 0 represent no difference between sites or within sites[59]. ANOSIM p values that are lower than 0.05 imply a higher

similarity within sites. Richness (Observed OTUs/ASVs) and Pielou's~~ or Species~~ evenness for each community type were calculated for estimating the difference of alpha-diversity. The analyses of alpha-diversity, NMDS and ANOSIM were performed using R package “vegan” v2.5-6. Dynamic change of community types was showed according to collected dates of specimens with ~~R package~~ ‘pheatmap’ package in R~~P heatmap’~~. Furthermore, to compensate for the effects of sample size, the Margalef's index was calculated by dividing the number of species in a sample by the natural log of the number of organisms collected[15]. For association between community types and potential confounding factors such as sex, age, virus existence and antibiotic use, the fisher exact test based on sample count was performed and the association with FDR-corrected p value <0.05 was considered significant.

Indicator analysis in throat and gut community types

According to the definition given by the United Nations Environment Programme (1996), the indicator species are a group of species whose status provides information on the overall condition of the ecosystem and of other species in that ecosystem, reflecting the quality and changes in environmental conditions as well as aspects of community composition. To obtain the reliable indicator genus that is specific to each community type, we performed the Indicator Species Analysis using the indicspecies package (ver.1.7.8) [60] in R platform with top 30 genus contributing to DMM clustering in both throat (accounting for 66% cumulative difference) and gut (68% cumulative difference). Dynamic changes of indicator genera corresponding to each throat community type were showed in all COVID-19 patients using the pheatmap package in R and only gut indicator genera with indicator values that were above 0.05 were presented in the patients.

Co-occurrence network analysis of a crosstalk between throat and gut microbiota

Based on microbial genus abundances normalized by the centered log ratio transformation of both throat and gut samples collected from 13 COVID-19 patients at the same time point, we calculated the Pearson Correlation Coefficient (Pearson’s r) among the throat & gut microbial genera. The Pearson’s r with *P* values that were below 0.05 after the FDR adjustment were considered significant correlations. Co-occurrence network of significantly correlated microbial genus pairs was visualized using Cytoscape v3.8.0[61].

Data availability

The raw data of 16S rRNA gene sequences are available at NCBI Sequence Read Archive (SRA) (https://www.ncbi.nlm.nih.gov/sra/) at BioProject ID PRJNA639286.

Supplemental information

Supplemental information includes supplemental Experimental Procedures, six figures, and three tables and can be found with this article.

Acknowledgements

We thank Ms Yingying Ma at Shanghai Public Health Clinical Center, Fudan University, and Mr. Kai Liu and Ms Xiuming Wu at Institut Pasteur of Shanghai, Chinese Academy of Sciences for their technical support. This work was supported by grants from the National Key Research and Development Program of China (2017ZX10103009-002, 2019YFC1200603 and 2018YFC2000500), Special fund for COVID-19 diagnosis and treatment of Nantong Science and Technology Bureau (SFCDT3-2), the Second Tibetan Plateau Scientific Expedition and Research (STEP) program (2019QZKK0503), the Key Research Program of the Chinese Academy of Sciences (FZDSW-219), and the Chinese National Natural Science Foundation (31970571).

Author contributions

C.Z. conceived the study idea. C.Z. and Z.Z. designed and supervised the study. R.L., W.C. and X.H. collected clinical samples and data. R.X. and R.L. performed the experiments. T.Z. and Q.W. processed and analyzed the raw sequencing data. R.X., R.L. and Z.W. analyzed the clinical data. Z.Z. and R.X. generated the figures. C.Z., Z.Z. and X.J. interpreted the data. C.Z., Z.Z., R.X., and T.Z. wrote the first draft of the manuscript. X.J. contributed to data interpretation and critical revision. All authors contributed to the final manuscript.

Competing interests: The authors have not conflict of interests.

Reference

1 Zhu, N. *et al.* A novel coronavirus from patients with pneumonia in China, 2019. *New Engl J Med* **382**,

727-733 (2020).

2 Zhou, P. *et al.* A pneumonia outbreak associated with a new coronavirus of probable bat origin. *Nature* **579**, 270-273 (2020).

3 Wu, Z. Y. & McGoogan, J. M. Characteristics of and important lessons from the Coronavirus Disease 2019 (COVID-19) outbreak in China smmary of a report of 72 314 cases from the Chinese Center for Disease Control and Prevention. *Jama-J Am Med Assoc* **323**, 1239-1242 (2020).

4 Guan, W. *et al.* Clinical characteristics of Coronavirus Disease 2019 in China. *New Engl J Med* **382**, 1708-1720 (2020).

5 Honda, K. & Littman, D. R. The microbiota in adaptive immune homeostasis and disease. *Nature* **535**, 75-84 (2016).

6 Dubourg, G., Edouard, S. & Raoult, D. Relationship between nasopharyngeal microbiota and patient's susceptibility to viral infection. *Expert Rev Anti-Infe* **17**, 437-447 (2019).

7 Man, W. H., Piters, W. A. A. D. & Bogaert, D. The microbiota of the respiratory tract: gatekeeper to respiratory health. *Nat Rev Microbiol* **15**, 259-270 (2017).

8 Wen, Z. *et al.* Distinct Nasopharyngeal and Oropharyngeal Microbiota of Children with Influenza A Virus Compared with Healthy Children. *Biomed Res Int* **2018**, 6362716 (2018).

9 Allen, E. K. *et al.* Characterization of the nasopharyngeal microbiota in health and during rhinovirus challenge. *Microbiome* **2**, 22 (2014).

10 de Steenhuijsen Piters, W. A. *et al.* Nasopharyngeal microbiota, host transcriptome, and disease severity in children with respiratory syncytial virus infection. *Am J Respir Crit Care Med* **194**, 1104-1115 (2016).

11 Xu, Y. *et al.* Characteristics of pediatric SARS-CoV-2 infection and potential evidence for persistent fecal viral shedding. *Nat. Med.*, 502-505 (2020).

12 Wu, Y. J. *et al.* Prolonged presence of SARS-CoV-2 viral RNA in faecal samples. *Lancet Gastroenterol. Hepatol.* **5**, 434-435 (2020).

13 Zuo, T. *et al.* Alterations in gut microbiota of patients with COVID-19 during time of hospitalization. *Gastroenterology* **S0016-5085**, 34701-34706 (2020).

14 Gu, S. *et al.* Alterations of the gut microbiota in patients with COVID-19 or H1N1 influenza. *Clin Infect Dis*, ciaa709 (2020).

15 Clifford, H. T. & Stephenson, W. in *An Introduction to Numerical Classification* (eds H. T. Clifford & W. Stephenson) 158-168 (Academic Press, 1975).

16 Burgener, E. B. *et al.* Filamentous bacteriophages are associated with chronic *Pseudomonas* lung infections and antibiotic resistance in cystic fibrosis. *Sci Transl Med* **11**, eaau9748 (2019).

17 Curran, C. S., Bolig, T. & Torabi-Parizi, P. Mechanisms and targeted therapies for *Pseudomonas aeruginosa* lung infection. *Am J Resp Crit Care* **197**, 708-727 (2018).

18 Abidi, M. Z., Ledeboer, N., Banerjee, A. & Hari, P. Morbidity and mortality attributable to Rothia bacteremia in neutropenic and nonneutropenic patients. *Diagn Micr Infec Dis* **85**, 116-120 (2016).

19 Lee, J. D. S., Chowdhury, N., Roberts, J. S. & Yilmaz, O. Host surface ectonucleotidase-CD73 and the opportunistic pathogen, Porphyromonas gingivalis, cross-modulation underlies a new homeostatic mechanism for chronic bacterial survival in human epithelial cells. *Virulence* **11**, 414-429 (2020).

20 Donati, C. *et al.* Uncovering oral Neisseria tropism and persistence using metagenomic sequencing. *Nat Microbiol* **1**, 16070 (2016).

21 Brennan, C. A. & Garrett, W. S. *Fusobacterium nucleatum* - symbiont, opportunist and oncobacterium. *Nat Rev Microbiol* **17**, 156-166 (2019).

22 Ramanan, P., Barreto, J. N., Osmon, D. R. & Tosh, P. K. Rothia Bacteremia: a 10-Year Experience at Mayo

Clinic, Rochester, Minnesota. *J Clin Microbiol* **52**, 3184-3189 (2014).

23 Tanaka, C. *et al.* A Lytic Bacteriophage for Controlling Pseudomonas lactis in Raw Cow's Milk. *Appl Environ Microbiol* **84** (2018).

24 La Rosa, S. L. *et al.* The human gut *Firmicute Roseburia* intestinalis is a primary degrader of dietary beta-mannans. *Nat Commun* **10**, 905 (2019).

25 Ozato, N. *et al.* Blautia genus associated with visceral fat accumulation in adults 20-76 years of age. *Npj Biofilms Microme* **5**, 28 (2019).

26 Valles-Colomer, M. *et al.* The neuroactive potential of the human gut microbiota in quality of life and depression. *Nat Microbiol* **4**, 623-632 (2019).

27 Lopez-Siles, M., Duncan, S. H., Garcia-Gil, L. J. & Martinez-Medina, M. Faecalibacterium prausnitzii: from microbiology to diagnostics and prognostics. *Isme J* **11**, 841-852 (2017).

28 Neumann, A., Bjorck, L. & Frick, I. M. *Finegoldia magna*, an anaerobic Gram-positive bacterium of the normal human microbiota, induces inflammation by activating neutrophils. *Front Microbiol* **11**, 65 (2020).

29 Patterson, A. M. *et al.* Human gut symbiont *Roseburia hominis* promotes and regulates innate immunity. *Front Immunol* **8**, 1166 (2017).

30 Hess, E. *et al. Capnocytophaga canimorsus* capsular serovar and disease severity, Helsinki Hospital District, Finland, 2000-2017. *Emerg Infect Dis* **24**, 2195-2201 (2018).

31 Kononen, E. & Wade, W. G. Actinomyces and Related Organisms in Human Infections. *Clin Microbiol Rev* **28**, 419-442 (2015).

32 Esaiassen, E. *et al. Bifidobacterium Bacteremia*: clinical characteristics and a genomic approach to assess pathogenicity. *J Clin Microbiol* **55**, 2234-2248 (2017).

33 Perlot, T. & Penninger, J. M. ACE2-from the renin-angiotensin system to gut microbiota and malnutrition. *Microbes Infect.* **15**, 866-873 (2013).

34 Huang, C. *et al.* Clinical features of patients infected with 2019 novel coronavirus in Wuhan, China. *Lancet* **395**, 497-506 (2020).

35 Dhar, D. & Mohanty, A. Gut microbiota and Covid-19- possible link and implications. *Virus Res* **285**, 198018 (2020).

36 Wypych, T. P., Wickramasinghe, L. C. & Marsland, B. J. The influence of the microbiome on respiratory health. *Nat Immunol* **20**, 1279-1290 (2019).

37 Dang, A. T. & Marsland, B. J. Microbes, metabolites, and the gut-lung axis. *Mucosal Immunol* **12**, 843-850 (2019).

38 Cao, W. & Li, T. S. COVID-19: towards understanding of pathogenesis. *Cell Res* **30**, 367-369 (2020).

39 Rooks, M. G. & Garrett, W. S. Gut microbiota, metabolites and host immunity. *Nat. Rev. Immunol.* **16**, 341-352 (2016).

40 Seifert, H. S. Location, Location, Location-Commensalism, Damage and Evolution of the Pathogenic Neisseria. *J Mol Biol* **431**, 3010-3014 (2019).

41 Zhang, D. & Frenette, P. S. Cross talk between neutrophils and the microbiota. *Blood* **133**, 2168-2177 (2019).

42 Ai, J. W., Li, Y., Zhou, X. & Zhang, W. H. COVID-19: treating and managing severe cases. *Cell Res* **30**, 370-371 (2020).

43 Lu, R. *et al.* Epidemiological and clinical characteristics of COVID-19 patients in Nantong, China. *J Infect Dev Ctries* **14**, 440-446 (2020).

44 D'Amore, R. *et al.* A comprehensive benchmarking study of protocols and sequencing platforms for 16S rRNA community profiling. *Bmc Genomics* **17** (2016).

45 de Muinck, E. J., Trosvik, P., Gilfillan, G. D., Hov, J. R. & Sundaram, A. Y. M. A novel ultra high-throughput 16S rRNA gene amplicon sequencing library preparation method for the Illumina HiSeq platform. *Microbiome* **5** (2017).

46 Edgar, R. C. UPARSE: highly accurate OTU sequences from microbial amplicon reads. *Nature Methods* **10**, 996-998 (2013).

47 Hannon, G. J. FASTX-Toolkit. *http://hannonlab.cshl.edu/fastx_toolkit* (2010).

48 Knight, R. *et al.* Best practices for analysing microbiomes. *Nature Reviews Microbiology* **16**, 410-422 (2018).

49 Callahan, B. J. *et al.* DADA2: High-resolution sample inference from Illumina amplicon data. *Nature Methods* **13**, 581-583 (2016).

50 Amir, A. Deblur rapidly resolves single-nucleotide community sequence patterns. *mSystems.* **2** (2017).

51 Caporaso, J. G. *et al.* QIIME allows analysis of high-throughput community sequencing data. *Nature Methods* **7**, 335-336 (2010).

52 Wang, Q., Garrity, G. M., Tiedje, J. M. & Cole, J. R. Naïve Bayesian Classifier for Rapid Assignment of rRNA Sequences into the New Bacterial Taxonomy. *Applied and Environmental Microbiology* **73**, 5261 (2007).

53 Cole, J. R. *et al.* Ribosomal Database Project: data and tools for high throughput rRNA analysis. *Nucleic Acids Research* **42**, D633-D642 (2013).

54 Holmes, I., Harris, K. & Quince, C. Dirichlet Multinomial Mixtures: Generative Models for Microbial Metagenomics. *PLOS ONE* **7**, e30126 (2012).

55 Stewart, C. J. *et al.* Temporal development of the gut microbiome in early childhood from the TEDDY study. *Nature* **562**, 583-588 (2018).

56 Ding, T. & Schloss, P. D. Dynamics and associations of microbial community types across the human body. *Nature* **509**, 357-360 (2014).

57 Fujimura, K. E. *et al.* Neonatal gut microbiota associates with childhood multisensitized atopy and T cell differentiation. *Nature Medicine* **22**, 1187-1191 (2016).

58 Schloss, P. D. *et al.* Introducing mothur: open-source, platform-independent, community-supported software for describing and comparing microbial communities. *Appl Environ Microbiol* **75**, 7537-7541 (2009).

59 Clarke, K. R. Non-parametric multivariate analyses of changes in community structure. *Australian Journal of Ecology* **18**, 117-143 (1993).

60 De Cáceres, M., Legendre, P. & Moretti, M. Improving indicator species analysis by combining groups of sites. *Oikos* **119**, 1674-1684 (2010).

61 Otasek, D., Morris, J. H., Boucas, J., Pico, A. R. & Demchak, B. Cytoscape Automation: empowering workflow-based network analysis. *Genome Biol* **20**, 185 (2019).

Figure legends

Figure 1. DMM clustering of 16S rRNA gene sequencing data of throat microbiota (*N* = 112). Dirichlet multinomial mixtures (DMM) modelling was applied to 16S rRNA gene sequencing. The entire dataset formed six distinct clusters based on lowest Laplace approximation. Bacterial taxa marked by the stars represent unclassified bacteria genera.

a. Heat map showing the relative abundance of the 30 most dominant bacterial genera per DMM cluster. The stars represent unclassified genera. NP, enriched in Non-COVID-19 patients. H, enriched in Healthy individuals. I-IV enriched in COVID-19 patients.

b. Nonmetric multidimensional scaling (NMDS) visualization of DMM clusters using Bray-Curtis distance of throat bacterial genera. The ANOSIM statistic R closer to 1 with < 0.05 P value suggest significant separation of microbial community structures. The stress value that was lower than 0.2 provides a good representation in reduced dimensions.

c. Box plots showing the alpha-diversity (richness and evenness) per each DMM cluster.

d. Indicators of airway microbial community types (DMM clusters) identified from top 30 genus contributing to throat microbial community typing (DMM clustering) in a. * P <0.05, ** P <0.01, and *** P < 0.001.

e. Dynamic shift of four throat microbial community types (DMM clusters) in different COVID-19 stages. Empty boxes indicate samples were unavailable in COVID-19 patients. Ages (years) were shown in parenthesis. NA, unavailable.

Figure 2. DMM clustering of 16S rRNA gene sequencing data of gut microbiota (*N* = 45). Dirichlet multinomial mixtures (DMM) modelling was applied to 16S rRNA gene sequencing. The entire dataset formed three distinct clusters based on lowest Laplace approximation. All samples were collected from COVID-19 patients. Bacterial taxa marked by the stars represent unclassified bacteria genera.

a. Heat map showing the relative abundance of the 30 most dominant bacterial genera per DMM cluster.

b. Nonmetric multidimensional scaling (NMDS) visualization of DMM clusters using Bray-Curtis distance of gut bacterial genera. The ANOSIM statistic R closer to 1 with < 0.05 P value suggest significant separation of microbial community structures. The stress value that was lower than 0.2

provides a good representation in reduced dimensions.

c. Box plots showing the alpha-diversity (richness and evenness) per each DMM cluster.

d. Indicators of gut microbial community types (DMM clusters) identified from top 30 genus contributing to gut microbial community typing (DMM clustering) in a. * P <0.05, ** P <0.01, and *** P < 0.001.

e. Dynamic shift of gut microbial community types (DMM clusters) in different COVID-19 stages. Empty boxes indicate samples were unavailable in COVID-19 patients. Ages (years) were shown in parenthesis.

Figure 3. Dynamic change of bacterial community types (DMM clusters) in respiratory tract and gut of patients with mild COVID-19.

Co-variation dynamics of throat and gut microbial communities of 13 COVID-19 patients. Filled circles indicate the presence of microbial community types. Positive or Negative detections of SARS--CoV-2 in gut or throat are implicated by + or - symbols, respectively. Age (months) of each COVID-19 adult was shown in brackets.

Figure 4. Dynamic change of 12 key taxa in respiratory tract and gut of patients with mild COVID-19.

Key taxa of DMM clusters and several core functional gut bacteria were shown in nine mild COVID-19 adults with at least two time points of sampling. Linked to Fig.1a, Fig.2a, and Supplementary Figs. S4 and S7.

Figure 5. Co-occurrence networks of gut and throat microbiota within 13 COVID-19 patients.

Pearson correlation was employed to calculate correlation coefficient (r) between bacterial genus pairs based on their relative abundances. Co-occurred pairs with r > 0.7 under FDR-adjusted P < 0.05 were shown and visualized by Cytoscape version 3.8.0. Edges were sized based on r values. The bigger squares or circles were indicators in Figs. 1d and 2d.

Supplementary materials

Supplementary table S1. Throat and gut microbial abundances (phyla and genera).

Supplementary table S2. The LPS level of the serum of patients.

Supplementary table S3. Clinical index of COVID-19 patients in this study.

Supplementary table S4. Dynamic changes of clinical parameters of 13 COVID-19 patients.

Supplementary figure S1. COIVD-19 patient admission and discharge time as well as the point of detection of SARS-CoV-2. a. the hospitalization of p13 was 40 days. b. the information of these patients was unavailable. DAY 1 was the day of symptom onset. Some COVID-19 patients were initially found/confirmed elsewhere and their samples were unavailable for this study. A lack of positive samples in some patients (e.g. P05, P11 and P25) were due to the unavailability of early positive samples.

Supplementary figure S2. Group distribution characteristics of each community cluster. Total number is shown in parentheses.

Supplementary figure S3. Margalef index of each community cluster. Margalef's index of clusters showed similar trends with richness as other analyses of Richness in both throat and anal samples. Margalef's index for each sample is calculated with the number of species (n) in sample minus 1 divided by the natural logarithm of the total sample count of cluster (N) in which the sample was included. Margalef's index = (n-1) / ln(N).

Supplementary figure S4. Time-scale changes of indicators of throat microbial community types. Color sectors represent relative abundance of indicators in different COVID-19 stages. Linked to **Figure 1**.

Supplementary figure S5. Dynamic changes of alpha-diversity since appearance of symptoms

in COVID-19 patients.

Supplementary figure S6. Dynamic changes of microbiome diversity over time during COVID-19 in eight patients. a) Richness, b) Peilou evenness.

Supplementary Figure S7. Time-scale changes of indicators of gut microbial community types. Color sectors represent relative abundance of indicators in different COVID-19 stages. Linked to **Figure 2**.

Supplementary Figure S8. Enrichment analysis of impact factors on throat microbial community typing. a) Sex, b) Age, c) Virus detection, and d) Antibiotic uses. Enrichment analysis was performed by using the Fisher's exact test under FDR-adjusted P < 0.05. Sample numbers were shown on the bar. Only COVID-19 patients were used for this analysis.

Supplementary Figure S9. Enrichment analysis of impact factors on gut microbial community typing. a) Sex, b) Age, c) Virus detection, and d) Antibiotic uses. Enrichment analysis was performed by using the Fisher's exact test under FDR-adjusted P < 0.05. No significant enrichment was observed. Sample numbers were shown on the bar.

Supplementary figure S10. Correlation of microbiome diversity with clinical parameters. a) Correlation between Anal swab microbiota diversity index and clinical parameters (Correlation coefficient (r) were marked in cells for correlation pairs with p<0.05). b) Correlation between Throat swab microbiota diversity index and clinical parameters (Correlation coefficient (r) were marked in cells for correlation pairs with p<0.05).

Supplementary figure S11. Correlation between microbiome diversity and LPS levels. a) Richness, b) Peilou evenness.

Supplementary Figure S12. Comparisons of indicator genera between throat and gut microbial clusters. Shared indicator genera are highlighted by red arrows.

Supplementary Figure S13. Putative restoration model of the respiratory and gut microbiomes over time in adults with mild COVID-19. SARS-CoV-2 infection resulted in a fast dysbiosis in both the respiratory tract and gut at the very early phase of the disease. A fast restoration of both the respiratory and gut microbiomes from early dysbiosis towards late more diverse status was observed in most adults with mild COVID-19 albeit they seemed to have a relatively slow clinical recovery. This model reflects the major microbiome dynamic change in most adults with mild COVID-19 but not all features in all patients, especially in those with severe disease.

REVIEWERS' COMMENTS:

Reviewer #1 (Remarks to the Author):

The manuscript from Xu and colleagues has improved dramatically and most of my comments have been addressed. Please find below some suggestions that I believe will help to improve the clarity of the paper:

Please add that all confirmed COVID-19 cases are hospitalized in China, even if they have no symptoms. This is not a common procedure in most countries, and being able to sample patients at the beginning of the infection is a massive advantage of this study.

Abstract (L38-39): the results of this study do not 'suggest that modulations of regional microbiota might help to improve the recovery of COVID-19 patients', as microbiome modulation experiments have not been performed here. I suggest rephrasing this sentence.

L46: This sentence indicates that only the elderly and people with comorbidities have severe COVID-19 symptoms, but we now know several cases of healthy individuals with severe reactions to the virus. Please rephrase.

L189: 'who experienced a worsening gut microbiome' – in terms of diversity? Or how do you define a worsening microbiome?

L200-201: The LPS was a good addition but needs to be explained better. Please explain the association between LPS and your conclusions from these analysis.

L217: I don't think you can be so confident here. Maybe rephrase to 'these types possibly reflect different…'

L219: The conclusion that 'COVID-19 are mainly characterized by community type II,' comes out of the blue here. It also gives the impression that community type II is the same in the gut and the respiratory microbiome. Maybe rephrase with more qualitative indications of what are the predominant features found in most COVID-19 patients.

L354: The paragraph first says 35 COVID patients, and this line says 38. Please clarify.

Lines 389 and 401. Please indicate how many samples remained in the analyses (in each group) after removing samples with low sequencing depth.

L 458-459: Please update this sentence. No 'supplemental experimental procedures' have been provided, and the number of figures and tables do not match.

L649: Is the age given in months (as stated here in the caption) or in years (as indicated in the figure).

L713: The 'anal swab' does not have a microbiota. Consider rephrasing it here (and elsewhere) to rectum microbiota.

Please embed the supplementary figure captions in the supplementary figures.

In order to make this study reproducible, please provide the analyses scripts/code as supplementary material. Very little information is given for the CLR transformation for example (e.g. what number was added (offset) to avoid log transformation with zeros?). These details can change the results, and therefore would ideally be provided with the R and/or Shell scripts.

Reviewer #2 (Remarks to the Author):

I would like to thank the authors for meticulously addressing all of my questions and concerns. Although I still see important limitations of the present work, these are now more explicitly discussed and the authors have invested a lot of effort in solving the problems at hand with the best possible methodological approach. I support a publication of the manuscript in the present form.

Reviewer #3 (Remarks to the Author):

I thank the authors for their time spent reviewing this manuscript and listening to suggestions, and apologise for my late response. I believe this manuscript has been significantly improved by acknowledging the limitations of their study more clearly and limiting the strength of their conclusions to better fit the data.

All line references are on "tracked changes" version.
Major Comments:
-BLAST scores (fig R2 and R4; lines 102-114; line 287) are used to identify select species of bacteria based on the samples with highest sequence similarity, yet the results do not reflect any level of selectivity in the results. E.g. in figure R4, the authors identify P. lactis as the species with highest sequence similarity, yet all other species listed also had 100% sequence identity with equal coverage and E value. It is not appropriate to claim a definitive identification based on these results, and the authors should rephrase the document to address the ambiguity. 16S sequencing does not have the sample level of specificity as metagenomics and results should be interpreted with more generality if the specificity is not available for a given taxa.
-Lines 150-157: the authors describe that 8/10 patients experienced a shift to higher diversity, but then list 2 patients who maintained stable communities and one who had an opposite shift. This is a total of 11 patients – please check these numbers. Similarly, in Lines 160-167, the authors describe 7/8 patients having synchronous changes in communities, but then describe 6 which had similar changes, and 2 which did not. Please check these numbers
-Line 319-321: Please make the language less definitive. While the use of biomarkers is possible, the data presented here is not sufficient to suggest it. I suggest changing "can be" to "may potentially be". In fact, the whole concluding paragraph should be cautious not to overstate the therapeutic benefits given the limitations of this study as outlined in the rest of the discussion.

Minor Comments:
-Line 52: Change "affects" to "affect"
-Line 58: change "has" to "have"
-Line 145: change "bacteria" to "genera"
-line 205: change "trended" to "tended"

Response to reviewers' comments

Reviewer #1:

Remarks to the Author:

The manuscript from Xu and colleagues has improved dramatically and most of my comments have been addressed. Please find below some suggestions that I believe will help to improve the clarity of the paper:

Author: Thank you for your valuable suggestions to improve our manuscript.

Please add that all confirmed COVID-19 cases are hospitalized in China, even if they have no symptoms. This is not a common procedure in most countries, and being able to sample patients at the beginning of the infection is a massive advantage of this study.

Author: We added this sentence (L69-70).

Abstract (L38-39): the results of this study do not 'suggest that modulations of regional microbiota might help to improve the recovery of COVID-19 patients', as microbiome modulation experiments have not been performed here. I suggest rephrasing this sentence.

Author: We rephrased this sentence. It is now "which may provide valuable information for other therapeutic alternatives such as modulations of regional microbiota".

L46: This sentence indicates that only the elderly and people with comorbidities have severe COVID-19 symptoms, but we now know several cases of healthy individuals with severe reactions to the virus. Please rephrase.

Author: We rephrased this sentence. It is now "COVID-19 more likely develops into severe pneumonia and cause death in elderly over 60 years of age or those having comorbidities".

L189: 'who experienced a worsening gut microbiome' – in terms of diversity? Or how do you define a worsening microbiome?

Author: Yes, it is based on diversity. We rephrased this sentence. It is now "… patient (p07) accompanied by a decreasing bacterial diversity (Fig. 4 and Supplementary Fig. S6)".

L200-201: The LPS was a good addition but needs to be explained better. Please explain the association between LPS and your conclusions from these analysis.

Author: We added the description in L200-203: "High serum LPS is due to microbial

translocation, and was often associated with virus infection. High serum LPS levels were also detected in some COVID-19 patients (Supplementary Table S2), suggesting that bacteria translocation might play a role in the cross-talk between the respiratory and gut microbiomes."

L217: I don't think you can be so confident here. Maybe rephrase to 'these types possibly reflect different…'
Author: We changed the sentence as you suggested.

L219: The conclusion that 'COVID-19 are mainly characterized by community type II,' comes out of the blue here. It also gives the impression that community type II is the same in the gut and the respiratory microbiome. Maybe rephrase with more qualitative indications of what are the predominant features found in most COVID-19 patients.
Author: We changed the sentence to "Second, the microbiome community types with lower alpha-diversity more likely appears in the early phase of COVID-19, and upper respiratory and gut microbiomes altered by COVID-19 are mainly characterized by community type II with predominance of *Bacteroidales*, *Fusobacterium*, *Porphyromonas*, *Prevotella*, *Neisseria* and some opportunistic pathogens in the former, and *Neisseria* in the latter."

Lines 389 and 401. Please indicate how many samples remained in the analyses (in each group) after removing samples with low sequencing depth.
Author: We added the number.

L354: The paragraph first says 35 COVID patients, and this line says 38. Please clarify.
L 458-459: Please update this sentence. No 'supplemental experimental procedures' have been provided, and the number of figures and tables do not match.
L649: Is the age given in months (as stated here in the caption) or in years (as indicated in the figure).
L713: The 'anal swab' does not have a microbiota. Consider rephrasing it here (and elsewhere) to rectum microbiota.
Author: We corrected these errors.

Please embed the supplementary figure captions in the supplementary figures.
Author: We did.

In order to make this study reproducible, please provide the analyses scripts/code as

supplementary material. Very little information is given for the CLR transformation for example (e.g. what number was added (offset) to avoid log transformation with zeros?). These details can change the results, and therefore would ideally be provided with the R and/or Shell scripts.

Author: As suggested, we provided the analyses scripts/code of the correlation analysis R package as supplemental material. We used the clr function in the R package composions, without changing the value in the data for the central log transformation.

Reviewer #2:

Remarks to the Author:

I would like to thank the authors for meticulously addressing all of my questions and concerns. Although I still see important limitations of the present work, these are now more explicitly discussed and the authors have invested a lot of effort in solving the problems at hand with the best possible methodological approach. I support a publication of the manuscript in the present form.

Author: Thank you for your positive comments.

Reviewer #3:

Remarks to the Author:

I thank the authors for their time spent reviewing this manuscript and listening to suggestions, and apologise for my late response. I believe this manuscript has been significantly improved by acknowledging the limitations of their study more clearly and limiting the strength of their conclusions to better fit the data.

Author: Thank you for the positive comments.

All line references are on “tracked changes” version.

Author: We did.

Major Comments:

-BLAST scores (fig R2 and R4; lines 102-114; line 287) are used to identify select species of bacteria based on the samples with highest sequence similarity, yet the results do not reflect any level of selectivity in the results. E.g. in figure R4, the authors identify P. lactis as the species with highest sequence similarity, yet all other species listed also had 100% sequence identity with equal coverage and E value. It is

not appropriate to claim a definitive identification based on these results, and the authors should rephrase the document to address the ambiguity. 16S sequencing does not have the sample level of specificity as metagenomics and results should be interpreted with more generality if the specificity is not available for a given taxa.

Author: We agree with the reviewer' comments. Indeed, our representative sequences of *Pseudomonas* genus have the highest sequence similarity (100%) with multiple known non-pathogenic species such as *P. lactis*, *P. paralactis*, *P. canadensis*, *P. tolaasii*, and *P. fluorescens* and so on. These blast results suggested that it is very difficult to achieve the identification of species level of *Pseudomonas* genus using 16S rRNA sequences from this study. Even so, we believe, compared with the community type H, a decreased alpha-diversity with high abundance of opportunistic pathogenic and environmental bacteria (*Pseudomonas spp.*) in community types II-IV might imply unfavorable or abnormal microbiomes in the respiratory tract. Accordingly, we rephased related descriptions.

Sequences producing significant alignments Download Manage columns Show 100

select all *100 sequences selected* GenBank Graphics Distance tree of results

Description	Max Score	Total Score	Query Cover	E value	Per. Ident	Accession
Pseudomonas lactis strain DSM 29167 16S ribosomal RNA, partial sequence	462	462	100%	3e-130	100.00%	NR_156986.1
Pseudomonas paralactis strain DSM 29164 16S ribosomal RNA, partial sequence	462	462	100%	3e-130	100.00%	NR_156987.1
Pseudomonas canadensis strain 2-92 16S ribosomal RNA, partial sequence	462	462	100%	3e-130	100.00%	NR_156852.1
Pseudomonas tolaasii strain ATCC 33618 16S ribosomal RNA, partial sequence	462	462	100%	3e-130	100.00%	NR_114481.1
Pseudomonas tolaasii strain NBRC 103163 16S ribosomal RNA, partial sequence	462	462	100%	3e-130	100.00%	NR_114227.1
Pseudomonas fluorescens strain NBRC 14160 16S ribosomal RNA, partial sequence	462	462	100%	3e-130	100.00%	NR_113647.1
Pseudomonas azotoformans strain NBRC 12693 16S ribosomal RNA, partial sequence	462	462	100%	3e-130	100.00%	NR_113600.1
Pseudomonas lurida strain P 513/18 16S ribosomal RNA, partial sequence	462	462	100%	3e-130	100.00%	NR_042199.1
Pseudomonas tolaasii 16S ribosomal RNA, partial sequence	462	462	100%	3e-130	100.00%	NR_117823.1
Pseudomonas trivialis strain P 513/19 16S ribosomal RNA, partial sequence	462	462	100%	3e-130	100.00%	NR_028987.1
Pseudomonas poae strain P 527/13 16S ribosomal RNA, partial sequence	462	462	100%	3e-130	100.00%	NR_028986.1
Pseudomonas fluorescens strain CCM 2115 16S ribosomal RNA, partial sequence	462	462	100%	3e-130	100.00%	NR_115715.1
Pseudomonas simiae strain OLi 16S ribosomal RNA, partial sequence	462	462	100%	3e-130	100.00%	NR_042392.1
Pseudomonas tolaasii NCPPB 2192 16S ribosomal RNA, partial sequence	462	462	100%	3e-130	100.00%	NR_114595.1
Pseudomonas meridiana strain CMS 38 16S ribosomal RNA, partial sequence	462	462	100%	3e-130	100.00%	NR_025587.1
Pseudomonas antarctica strain CMS 35 16S ribosomal RNA, partial sequence	462	462	100%	3e-130	100.00%	NR_025586.1
Pseudomonas fluorescens strain ATCC 13525 16S ribosomal RNA, partial sequence	462	462	100%	3e-130	100.00%	NR_114476.1
Pseudomonas costantinii strain CFBP 5705 16S ribosomal RNA, partial sequence	462	462	100%	3e-130	100.00%	NR_025164.1
Pseudomonas extremorientalis strain KMM 3447 16S ribosomal RNA, partial sequence	462	462	100%	3e-130	100.00%	NR_025174.1
Pseudomonas tolaasii strain LMG 2342 16S ribosomal RNA, partial sequence	462	462	100%	3e-130	100.00%	NR_041799.1

-Lines 150-157: the authors describe that 8/10 patients experienced a shift to higher diversity, but then list 2 patients who maintained stable communities and one who had an opposite shift. This is a total of 11 patients – please check these numbers. Similarly, in Lines 160-167, the authors describe 7/8 patients having synchronous changes in communities, but then describe 6 which had similar changes, and 2 which did not. Please check these numbers.

Author: We corrected the error.

-Line 319-321: Please make the language less definitive. While the use of biomarkers is possible, the data presented here is not sufficient to suggest it. I suggest changing

“can be” to “may potentially be”. In fact, the whole concluding paragraph should be cautious not to overstate the therapeutic benefits given the limitations of this study as outlined in the rest of the discussion.

Author: We did.

Minor Comments:

-Line 52: Change “affects” to “affect”

-Line 58: change “has” to “have”

-Line 145: change “bacteria” to “genera”

-line 205: change “trended” to “tended”

Author: We did all changes.
